# A molecular cell atlas of mouse lemur, an emerging model primate

**DOI:** 10.1038/s41586-025-09113-9

**Published:** 2025-07-30

**Authors:** The Tabula Microcebus Consortium, Antoine de Morree, Iwijn De Vlaminck, Liza Shapiro, Andriamahery Razafindrakoto, Hajanirina Noëline Ravelonjanahary, Patricia Wright, Anne D. Yoder, Cathy V. Williams, Robert Schopler, Ute Radespiel, Jean-Michel Verdier, Corinne Lautier, E. Christopher Kirk, Rebecca Lewis, Astrid Gillich, Zicheng Zhao, Elias Godoy, Jérémy Terrien, Jacques Epelbaum, Dita Gratzinger, Katherine Lucot, Thomas Montine, Jessica D’Addabbo, Isaac Bakerman, Patricia Nguyen, Aaron Kershner, Karim Mrouj, Philip Beachy, Thomas H. Ambrosi, Malachia Hoover, Alina Alam, Charles Chan, SoRi Jang, Avin Veerakumar, Peng Li, Andrea R. Yung, Connor V. Duffy, Song-Lin Ding, Ed S. Lein, Silvana Konermann, Liqun Luo, Trygve E. Bakken, Justus M. Kebschull, Rebecca D. Hodge, Taichi Isobe, Michael F. Clarke, Biter Bilen, Jean Farup, Andoni Urtasun, Jengmin Kang, Ming Chen, BaoXiang Li, Varun Ramanan Subramaniam, Shravani Mukherjee, Aditi Swarup, Lily Kim, Bronwyn Scott, Ahmad Al-Moujahed, Albert Y. Wu, Douglas Vollrath, Nicholas Schaum, Amanda L. Wiggenhorn, Tony Wyss-Coray, Jonathan Z. Long, Yin Liu, Ahmad Nabhan, Gabriel Loeb, Shengda Lin, Honor Paine, Deviana Burhan, Aris Taychameekiatchai, Bruce Wang, F. Hernán Espinoza, Christin Kuo, Ross Metzger, Zhen Qi, Rebecca Culver, Kerwyn C. Huang, Patrick Neuhöfer, Charles A. Chang, Yan Hang, Seung K. Kim, Hannah N. W. Weinstein, Paul Allegakoen, Franklin W. Huang, Sivakamasundari, Song Eun Lee, Hannah K. Frank, Scott D. Boyd, Wan-Jin Lu, Ankit Baghel, William Kong, Carly Israel, Ashley Maynard, Michelle Tan, Youcef Ouadah, Jalal Baruni, Timothy Ting-Hsuan Wu, Robert C. Jones, Spyros Darmanis, Sheela Crasta, Jia Yan, Aditi Agrawal, Shelly Huynh, Brian Yu, James Webber, Weilun Tan, Saba Nafees, Zhengda Li, Michael F. Z. Wang, Roozbeh Dehghannasiri, Julia Olivieri, Julia Salzman, Lisbeth A. Guethlein, Peter Parham, Qiuyu Jing, Jane Antony, Geoff Stanley, Jinxurong Yang, Winston Koh, Sheng Wang, Snigdha Agarwal, Kyle Awayan, Erin McGeever, Venkata N. P. Vemuri, Pranav V. Lalgudi, Camille Ezran, Shixuan Liu, Stephen Chang, Jingsi Ming, Olga Botvinnik, Lolita Penland, Alexander Tarashansky, Kyle J. Travaglini, Jia Zhao, Gefei Wang, Kazuteru Hasegawa, Hosu Sin, Rene Sit, Jennifer Okamoto, Rahul Sinha, Yue Zhang, Caitlin J. Karanewsky, Jozeph L. Pendleton, Maurizio Morri, Martine Perret, Fabienne Aujard, Lubert Stryer, Steven Artandi, Margaret T. Fuller, Irving L. Weissman, Thomas A. Rando, James E. Ferrell, Bo Wang, Can Yang, Kerriann M. Casey, Megan A. Albertelli, Angela Oliveira Pisco, Jim Karkanias, Norma Neff, Angela Ruohao Wu, Stephen R. Quake, Mark A. Krasnow, Camille Ezran, Shixuan Liu, Stephen Chang, Jingsi Ming, Olga Botvinnik, Lolita Penland, Alexander Tarashansky, Antoine de Morree, Kyle J. Travaglini, Jia Zhao, Gefei Wang, Kazuteru Hasegawa, Hosu Sin, Rene Sit, Jennifer Okamoto, Rahul Sinha, Yue Zhang, Caitlin J. Karanewsky, Jozeph L. Pendleton, Maurizio Morri, Martine Perret, Fabienne Aujard, Lubert Stryer, Steven Artandi, Margaret T. Fuller, Irving L. Weissman, Thomas A. Rando, James E. Ferrell, Bo Wang, Iwijn De Vlaminck, Can Yang, Kerriann M. Casey, Megan A. Albertelli, Angela Oliveira Pisco, Jim Karkanias, Norma Neff, Angela Ruohao Wu, Stephen R. Quake, Mark A. Krasnow

**Affiliations:** 1 Department of Biochemistry, Stanford University School of Medicine, Stanford, CA USA; 2 Howard Hughes Medical Institute, Stanford University School of Medicine, Stanford, CA USA; 3 Department of Chemical and Systems Biology, Stanford University School of Medicine, Stanford, CA USA; 4 Division of Cardiovascular Medicine, Stanford University School of Medicine, Stanford, CA USA; 5 KLATASDS-MOE, School of Statistics and Academy of Statistics and Interdisciplinary Sciences, East China Normal University, Shanghai, China; 6 Chan Zuckerberg Biohub, San Francisco, CA USA; 7 Department of Bioengineering, Stanford University, Stanford, CA USA; 8 Department of Neurology and Neurological Sciences, Stanford University School of Medicine, Stanford, CA USA; 9 Department of Biomedicine, Aarhus University, Aarhus, Denmark; 10 Department of Mathematics, Hong Kong University of Science and Technology, Hong Kong SAR, China; 11 Stanford Cancer Institute, Stanford University School of Medicine, Stanford, CA USA; 12 Department of Medicine, Stanford University School of Medicine, Stanford, CA USA; 13 Department of Developmental Biology, Stanford University School of Medicine, Stanford, CA USA; 14 Institute for Stem Cell Biology and Regenerative Medicine, Stanford University School of Medicine, Stanford, CA USA; 15 Department of Biology, Stanford University, Stanford, CA USA; 16 Adaptive Mechanisms and Evolution (MECADEV), UMR 7179, National Center for Scientific Research, National Museum of Natural History, Brunoy, France; 17 Department of Neurobiology, Stanford University School of Medicine, Stanford, CA USA; 18 Department of Genetics, Stanford University School of Medicine, Stanford, CA USA; 19 Nancy E. and Peter C. Meinig School of Biomedical Engineering, Cornell University, Ithaca, NY USA; 20 Department of Comparative Medicine, Stanford University School of Medicine, Stanford, CA USA; 21 Division of Life Science, Hong Kong University of Science and Technology, Hong Kong SAR, China; 22 Department of Chemical and Biological Engineering, Hong Kong University of Science and Technology, Hong Kong SAR, China; 23 Center for Aging Science, Hong Kong University of Science and Technology, Hong Kong SAR, China; 24 Department of Applied Physics, Stanford University, Stanford, CA USA; 25 Department of Anthropology, University of Texas at Austin, Austin, TX USA; 26 Department of Animal Biology, Faculty of Science, University of Antananarivo, Antananarivo, Madagascar; 27 Department of Anthropology, Stony Brook University, Stony Brook, NY USA; 28 Department of Biology, Duke University, Durham, NC USA; 29 Duke Lemur Center, Durham, NC USA; 30 Institute of Zoology, University of Veterinary Medicine Hannover, Hannover, Germany; 31 MMDN, University of Montpellier, EPHE-PSL, INSERM, Montpellier, France; 32 Unité Mixte de Recherche en Santé 894 INSERM, Centre de Psychiatrie et Neurosciences, Université Paris Descartes Sorbonne, Paris, France; 33 Department of Pathology, Stanford University School of Medicine, Stanford, CA USA; 34 Stanford Cardiovascular Institute, Stanford, CA USA; 35 Department of Urology, Stanford University School of Medicine, Stanford, CA USA; 36 Human Cell Types Department, Allen Institute for Brain Science, Seattle, WA USA; 37 Department of Biomedical Engineering, Johns Hopkins School of Medicine, Baltimore, MD USA; 38 Department of Oncology and Social Medicine, Kyushu University, Fukuoka, Japan; 39 Department of Ophthalmology, Stanford University School of Medicine, Stanford, CA USA; 40 Department of Chemistry, Stanford University, Stanford, CA USA; 41 Wu Tsai Neurosciences Institute, Stanford, CA USA; 42 Sarafan ChEM-H, Stanford, CA USA; 43 Division of Nephrology, Department of Medicine, University of California San Francisco, San Francisco, CA USA; 44 Zhejiang Provincial Key Laboratory for Cancer Molecular Cell Biology, Life Sciences Institute, Zhejiang University, Hangzhou, China; 45 Department of Medicine and Liver Center, University of California San Francisco, San Francisco, CA USA; 46 Department of Pediatrics, Stanford University School of Medicine, Stanford, CA USA; 47 Department of Microbiology and Immunology, Stanford University School of Medicine, Stanford, CA USA; 48 Stanford Diabetes Research Center, Stanford, CA USA; 49 JDRF Center of Excellence, Stanford, CA USA; 50 Division of Hematology/Oncology, Department of Medicine, University of California San Francisco, San Francisco, CA USA; 51 Helen Diller Family Comprehensive Cancer Center, University of California San Francisco, San Francisco, CA USA; 52 Bakar Computational Health Sciences Institute, University of California San Francisco, San Francisco, CA USA; 53 Department of Ecology and Evolutionary Biology, Tulane University, New Orleans, LA USA; 54 Department of Anesthesiology, Perioperative and Pain Medicine, Stanford University School of Medicine, Stanford, CA USA; 55 Department of Computational Biology, Cornell University, Ithaca, NY USA; 56 Department of Biomedical Data Science, Stanford University, Stanford, CA USA; 57 Institute for Computational and Mathematical Engineering, Stanford University, Stanford, CA USA; 58 Department of Structural Biology, Stanford University School of Medicine, Stanford, CA USA; 59 Institute of Bioengineering and Bioimaging, Agency of Science Technology and Research, Singapore, Singapore; 60 Bioinformatics Institute, Agency of Science Technology and Research, Singapore, Singapore; 61 Paul G. Allen School of Computer Science and Engineering, University of Washington, Seattle, WA USA

**Keywords:** Transcriptomics, Cell biology

## Abstract

Mouse lemurs are the smallest and fastest reproducing primates, as well as one of the most abundant, and they are emerging as a model organism for primate biology, behaviour, health and conservation. Although much has been learnt about their ecology and phylogeny in Madagascar and their physiology, little is known about their cellular and molecular biology. Here we used droplet-based and plate-based single-cell RNA sequencing to create Tabula Microcebus, a transcriptomic atlas of 226,000 cells from 27 mouse lemur organs opportunistically obtained from four donors clinically and histologically characterized. Using computational cell clustering, integration and expert cell annotation, we define and biologically organize more than 750 lemur molecular cell types and their full gene expression profiles. This includes cognates of most classical human cell types, including stem and progenitor cells, and differentiating cells along the developmental trajectories of spermatogenesis, haematopoiesis and other adult tissues. We also describe dozens of previously unidentified or sparsely characterized cell types. We globally compare expression profiles to define the molecular relationships of cell types across the body, and explore primate cell and gene expression evolution by comparing lemur transcriptomes to those of human, mouse and macaque. This reveals cell-type-specific patterns of primate specialization and many cell types and genes for which the mouse lemur provides a better human model than mouse^1^. The atlas provides a cellular and molecular foundation for studying this model primate and establishes a general approach for characterizing other emerging model organisms.

## Abstract (web summary)

Together with a companion paper, the generation of a transcriptomic atlas for the mouse lemur and analyses of example cell types establish this animal as a molecularly tractable primate model organism.

## Main

Systematic genetic and genomic studies of a handful of diverse organisms over the past half century have transformed our understanding of biology. But many important aspects of primate biology, behaviour, disease and conservation are absent or poorly modelled in mice or other established genetic model organisms^2,3^. Mouse lemurs (*Microcebus* spp.) are the smallest (about 50 g) and fastest reproducing primates (2 months of gestation, 8 months generation time, 1–4 offspring per pregnancy), as well as one of the most abundant (millions to tens of millions)^4^, and are an emerging primate model organism^5^. Although much has been learnt from laboratory studies of their physiology and ageing^6,7^ and from field studies in Madagascar of their ecology, behaviour and phylogeny^8,9^, little is known about their genetics or cellular and molecular biology.

To establish a new genetic model organism, the first step has traditionally been to characterize the wild type then to screen for phenotypes and map the underlying mutations, or to create and characterize targeted mutations, as is standard for mouse. Systematic screens are underway for mouse lemurs, leveraging their standing genetic diversity and the large pool of naturally occurring mutations. The next step is to create a genetic map or reference genome sequence, which is already available for the mouse lemur^10^ and has become increasingly affordable, accurate and complete with new sequencing techniques^11^. With the accompanying development of single-cell RNA sequencing (scRNA-seq) technologies, we reasoned that a reference transcriptomic cell atlas of wild-type lemurs would provide a molecular foundation that would aid the characterization of organ, cell and gene function. Moreover, such a resource would enable new types of cellular and molecular analysis, accelerate genetic mapping, and provide high resolution into primate evolution.

**Fig. 1:**
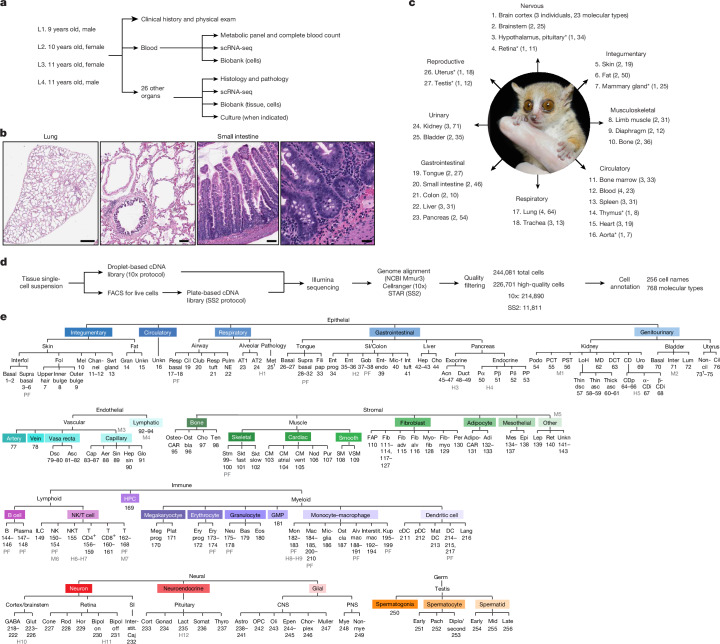
Construction of the mouse lemur cell atlas. **a**, Age and sex of the lemurs (L1–L4) profiled, the metadata collected and uses of the obtained tissues. **b**, Sections of lung (from lemur L1) and small intestine (from lemur L3), with close-ups on the right, stained with haematoxylin and eosin. *n* = 4. Scale bars (left to right), 1,000, 10, 100 and 25 µm. A histological atlas of all tissues analysed is available online (Tabula Microcebus portal), and histopathology is described in a separate study^15^. **c**, Summary of the tissues collected, showing for each the number of biological replicates (individuals) and the number of identified molecular cell types. Asterisks indicate technically challenging tissues to obtain with only one replicate. **d**, Scheme for obtaining and processing scRNA-seq data. **e**, Dendrogram of the 256 assigned designations for the 768 molecular cell types across the atlas, arranged by compartment (epithelial, endothelial, stromal, immune, neural, germ) then ordered by organ system (epithelial compartment) or biological relatedness (other compartments). Designation numbers are provided below the abbreviations. Closely related molecular types are grouped (shown separately in Supplementary Figs. 1 and 2). H1–12, hybrid types with symbols between types for which the hybrid shares expression signatures; M1–M7, mixed clusters of distinct cell types too few to assign separately; PF, proliferative state. Dagger indicates tumour cells^1^. See Extended Data Fig. 1 for the annotation pipeline. 1–2, interfollicular basal (Interfol); 3–6, interfollicular suprabasal; 7, upper hair follicle (Fol); 8, inner bulge; 9, outer bulge; 10, melanocyte (Mel); 11–12, channel; 13, sweat (Swt) gland; 14, granulosa (Gran); 15, unknown (Unkn) epithelial (CRISP3^+^); 16, unknown epithelial (PGAP1^+^); 17–18, respiratory (Resp) basal; 19, ciliated (Cil); 20, club; 21, respiratory tuft; 22, pulmonary neuroendocrine (Pulm NE); 23, alveolar epithelial type 1 (AT1); 24, alveolar epithelial type 2 (AT2); 25, uterine metastasis (Met); 26–27, basal; 28–32, suprabasal (Supra) basal; 33, filiform papilla (Fili pap); 34, enterocyte progenitor (Ent prog), small intestine (SI); 35–36, enterocyte; 37–38, goblet (Gob); 39, enteroendocrine (Ent-endo); 40, microfold (Mic-f); 41, intestinal (Int) tuft; 42–43, hepatocyte (Hep); 44, cholangiocyte (Cho); 45–47, acinar (Acn); 48–49, ductal (Duct); 50, pancreatic α (Pα); 51, pancreatic β (Pβ); 52, pancreatic δ (Pδ); 53, pancreatic polypeptide (PP); 54, podocyte (Podo); 55, proximal convoluted tubule (PCT); 56, proximal straight tubule (PST); 57, loop of Henle (LoH) thin descending (dsc) limb; 58–59, LoH thin ascending (asc) limb; 60–61, LoH thick ascending limb; 62, macula densa (MD); 63, distal convoluted tubule (DCT); 64–66, collecting duct principal (CDp); 67, α-intercalated (α-CDi); 68, β-intercalated (β-CDi); 69, urothelial (Uro); 70, basal urothelial; 71, intermediate (Inter) urothelial; 72, luminal (Lum) urothelial; 73–75, non-ciliated (Non-cil) epithelial of uterus; 76, ciliated (Cil) epithelial of uterus; 77, artery; 78, vein; 79–80, vasa recta descending limb; 81–82, vasa recta ascending limb; 83–87, capillary (Cap); 88, capillary aerocyte (Aer); 89, sinusoid (MAFB^+^) (Sin); 90, hepatic sinusoid; 91, glomerular endothelial (Glo); 92–94, lymphatic; 95, osteo-CAR; 96, osteoblast (Ostbla); 97, chondrocyte (Cho); 98, tendon (Ten); 99–100, skeletal muscle satellite stem (Stm); 101, skeletal fast muscle (Skt fast); 102, skeletal slow muscle (Skt slow); 103, cardiomyocyte (CM); 104, atrial cardiomyocyte; 105, ventricular (vent) cardiomyocyte; 106, nodal (Nod); 107, Purkinje (Pur); 108, smooth muscle (SM); 109, vascular-associated smooth muscle (VSM); 110, fibroadipogenic progenitor (FAP); 111–114 and 117–127, fibroblast (Fib); 115, adventitial fibroblast (Fib adv); 116, alveolar fibroblast (Fib alv); 128, myofibroblast (Myofib); 129, fibromyocyte (Fibmyo); 130, pericyte (Per); 131, adipo-CAR; 132–133, adipocyte (Adi); 134–137, mesothelial (Mes); 138, epicardial (Epi); 139, leptomeningeal (Lep); 140, reticular (Ret); 141, unknown stromal (NGFR^+^TNNT2^+^); 142, unknown stromal (COL15A1^+^PTGDS^+^); 143, unknown stromal (ST6GAL2^+^); 144–146, B cell; 147–148, plasma cell; 149, innate lymphoid cell (ILC); 150–154, NK cell; 155, NKT cell; 156–168, T cell; 169, haematopoietic precursor (HPC); 170, megakaryocyte progenitor (Meg prog); 171, platelet (Plat); 172, erythroid progenitor (Ery prog); 173–174, erythroid lineage (Ery); 175–178, neutrophil (Neu); 179, basophil (Bas); 180, eosinophil (Eos); 181, granulocyte–monocyte progenitor (GMP); 182–183, monocyte (Mon); 184–185 and 200–210, macrophage (Mac); 186, microglial (Mic-glia); 187, osteoclast (Ostcla); 188–191, alveolar macrophage (Alv mac); 192–194, interstitial macrophage (Interstit. mac); 195–199, Kupffer (Kup); 211, conventional DC (cDC); 212, plasmacytoid DC (pDC); 213, mature DC (Mat DC); 214–215 and 217, DC; 216, Langerhans (Lang); 218–222, GABAergic neuron (GABA); 223–226, glutamatergic neuron (Glut); 227, cone; 228, rod; 229, horizontal (Hor); 230, on-bipolar (Bipol on); 231, off-bipolar (Bipol off); 232, interstitial of Cajal (Interstit. Caj); 233, corticotroph (Cort); 234, gonadotroph (Gonad); 235, lactotroph (Lact); 236, somatotroph (Somat); 237, thyrotroph (Thyro); 238–241, astrocyte (Astro); 242, oligodendrocyte precursor (OPC); 243, oligodendrocyte (Oli); 244–245, ependymal (Epen); 246, choroid plexus (Chor-plex); 247, Muller; 248, myelinating Schwann (Mye); 249, non-myelinating Schwann (Non-mye); 250, spermatogonium; 251, early spermatocyte (Early); 252, pachytene spermatocyte (Pach); 253, diplotene/secondary spermatocyte (Diplo/Second); 254, early spermatid; 255, mid spermatid (Mid); 256, late spermatid (Late).

**Extended Data Fig. 1:**
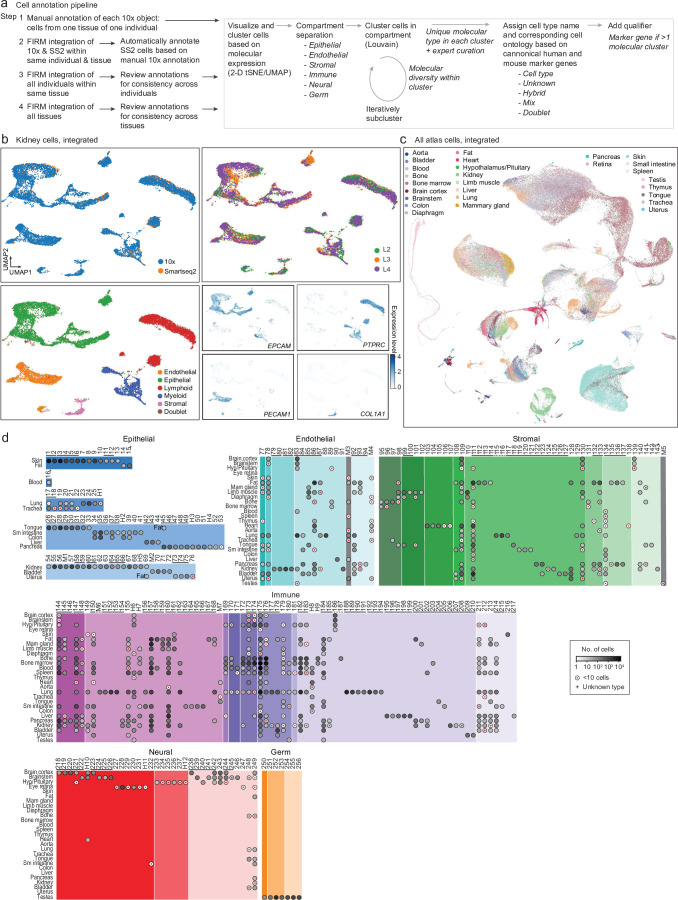
Taxonomy of identified mouse lemur molecular cell types. **a**. Scheme for multi-step scRNA-seq dataset integration, iterative cell clustering of cells with related transcriptomic profiles, and cell type annotation. **b**. Example tissue UMAP showing scRNA-seq profiles of all cells (dots) from an organ (kidney), integrated using FIRM algorithm across 10x and SS2 datasets (top left) and three individuals (top right). Compartment identities of the cell clusters are shown (bottom left) along with heat maps of expression levels (ln(UP10K+1) for 10x data, ln(CP10K+1) for SS2 data) of the indicated compartment marker genes (bottom right; *EPCAM*, epithelial; *PTPRC*, immune lymphoid/myeloid; *PECAM*, endothelial; *COL1A1*, stromal). **c**. UMAP of all 244,081 cells in the atlas integrated by FIRM across the 27 tissues analyzed from four individuals. **d**. Dot plot showing number of profiled cells (dot intensity shown by heat map scale, central red dot indicates <10 cells) for each of the 768 identified molecular cell types (including 38 hybrid types) plus 24 mixed clusters, isolated from the tissues indicated at left. Molecular cell types in each tissue (rows) are arranged (columns) by cell type designation number and separated by compartment as in Fig. 1e. Horizontal bars, closely related molecular types. +, unknown molecular type.

Here we set out to create a transcriptomic cell atlas of adult grey mouse lemur *Microcebus murinus* using a similar strategy to the one we previously applied to construct atlases of other organs and organisms^12–14^ (Fig. 1a–d and Extended Data Fig. 1a). We adapted the strategy to address several challenges for a new model organism. First, because there was no classical histological atlas, little molecular information and few cell-type markers, we relied on the extensive knowledge of human and mouse markers (Supplementary Table 1). Second, unlike classical model organisms but similar to human studies, donors were of different genetic backgrounds, ages and diseases. Hence, we collected extensive clinical data and histopathology on every donor and organ^15^ and, as in the previous Tabula Muris mouse atlases, we obtained multiple organ samples from each donor and processed them in parallel (Fig. 1a–c and Supplementary Table 2). This strategy helped us control for the many technical and biological variables, at least among cell profiles from the same donor. Finally, because this is a primate study, our strategy was opportunistic and designed to maximize information from each donor. To achieve our goal, we brought together experts from diverse fields, including mouse lemur biologists, veterinarians, pathologists, tissue experts, single-cell genomic specialists and computational leaders, to establish the Tabula Microcebus Consortium, which comprises a team of more than 150 collaborating scientists from over 50 laboratories at 15 institutions worldwide.

## Single-cell transcriptomics of 27 organs

Figure 1a–d outlines the approach we used to create a molecular cell atlas of the mouse lemur. Two male and two female aged laboratory mouse lemurs were euthanized for humane reasons owing to clinical conditions that failed to respond to therapy^15^. At euthanasia, blood was drawn and fresh tissues were rapidly isolated and divided into samples that were fixed for pathology or dissociated into cell suspensions for transcriptomic profiling using protocols optimized for each organ (Fig. 1a and Supplementary Methods). Full veterinary evaluation, clinical pathology and histopathological analyses are provided in a separate publication^15^, and metadata for each individual, organ and cell profiled are provided in the Supplementary Methods. This process created a classical histological atlas of the mouse lemur (Fig. 1b and online at the Tabula Microcebus portal).

From each individual, 3–24 organs were profiled by scRNA-seq, totalling 27 different organs, including the 19 analysed in mice for Tabula Muris^12,13^ (Fig. 1c and Supplementary Table 2). All organs were profiled in at least two animals, except seven sex-specific or technically challenging tissues that were profiled in only one individual. Single-cell suspensions were processed into RNA-seq libraries using the droplet-based 10x Chromium (10x) protocol and sequenced to saturation. For most organs, aliquots were also sorted by flow cytometry, and sequencing libraries were prepared robotically using the plate-based Smart-seq2 (SS2) protocol. The high throughput and lower cost of 10x enabled the profiling of more cells, whereas SS2 provided increased transcriptomic coverage that aided the detection of genes expressed at low levels, the discovery of unannotated genes and the characterization of gene structures^1^. Sequencing reads were aligned to the *M.* *murinus* reference genome^10^, and aligned reads were counted (as unique molecular identifiers (UMIs) for 10x and as reads for SS2) and then scaled using Seurat (v.2)^16^ to determine the expression level of each gene in each cell. After removing cells with low transcript expression, those compromised by index switching^17^ and putative cell doublets, we obtained 226,701 high-quality single-cell transcriptomic profiles: 214,890 from 10x (16,682–88,910 per individual) and 11,811 from SS2 (394–6,723 per individual) (Supplementary Table 2).

For cell annotation, profiles obtained using the 10x protocol were analysed separately for each organ and each individual through dimensionality reduction by principal component analysis (PCA), visualization in two-dimensional projections with t-stochastic neighbour embedding (t-SNE) and uniform manifold approximation and projection (UMAP) and clustering using the Louvain method in Seurat (v.2). For each obtained cluster of cells with similar profiles, the tissue compartment (epithelial, endothelial, stromal, immune, neural and germ) was assigned on the basis of the expression of lemur orthologues of compartment-specific mouse and human markers^14^ (Supplementary Table 1). Cells from each compartment were then separately and iteratively clustered until the differentially expressed genes (DEGs) that distinguished the resultant clusters were deemed not to be biologically relevant (for example, stress or ribosomal genes) (Extended Data Fig. 1a). Each cluster was assigned a cell-type designation, as detailed below.

We next integrated the SS2 data with the 10x data from the same organ and same individual using the integration algorithm FIRM^18^ (Extended Data Fig. 1a,b). The cell-type designation of each SS2 cell was automatically assigned on the basis of the designation of the neighbouring 10x cells and manually curated. We then used FIRM to integrate the combined 10x and SS2 datasets across all individuals profiled for the same organ and finally across all 27 organs into a single embedded space (Extended Data Fig. 1c). Cell-type designations were manually verified at each integration step to ensure consistency of nomenclature throughout the atlas. This approach identified 768 molecularly distinct cell populations (‘molecular cell types’) across the 27 profiled organs, with 28 ± 17 (mean ± s.d.) cell populations per organ and 294 ± 1,007 cells in each population, which were given 256 different cell-type designations (Fig. 1d,e, Extended Data Fig. 1d and Supplementary Table 2).

## Cell types and their expression profiles

To assign provisional identities and names to the 768 molecular cell types, we compiled a list of canonical marker genes for mouse and human cell types in each compartment of the 27 profiled organs and found the orthologous lemur genes (Methods and Supplementary Table 1). We then searched each organ for cell clusters enriched in the expression of each set of cell-type markers and assigned the clusters to the corresponding human and/or mouse cell-type name and cell ontology^19^. Note that for cell types with small numbers, we used expert, biologically guided manual curation. This approach enabled us to name almost all cell populations, although 34 classical cell types had multiple corresponding cell clusters, which we distinguished as molecular types by adding one or more DEGs to the cell designation. Supplementary Table 3 shows the DEGs enriched in each cell type relative to the entire atlas, to other cell types of the same tissue and to the same compartment of that tissue.

**Fig. 2:**
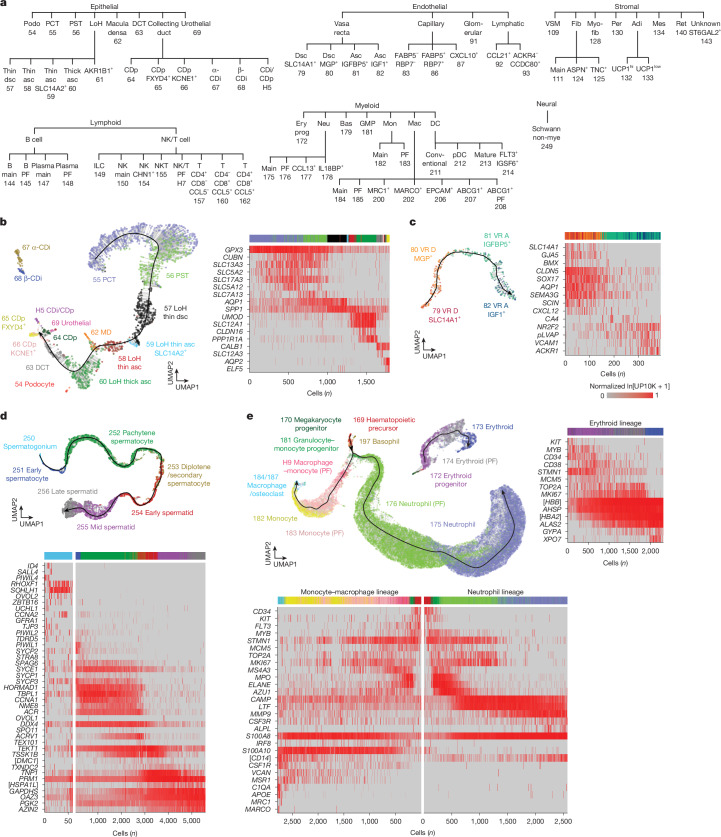
Organ cell types and gradients. **a**, Dendrogram of 71 identified kidney molecular types. **b**, Left, UMAP of kidney epithelial cells (L4, 10x). The trajectory (black line, cell density ridge) corresponds to the known cell spatial continuum along the nephron, beginning with the proximal convoluted tubule (55) and ending with the principal cells of the collecting duct (64–66). Note that macula densa cells (62) cluster between thin (58–59) and thick (60) ascending loop of Henle cell types and urothelial cells (69) near the principal cells of the collecting duct. Intercalated cells of the collecting duct (67–68) and podocytes (54) lie off the trajectory. Dots, individual cells coloured by molecular type; thin grey lines, cell alignments to the trajectory. Right, heatmap showing the relative expression of nephron markers along the trajectory ((ln[UP10K + 1] normalized for each gene to its maximal value (99.5 percentile) along the trajectory). Coloured bar, cell-type designations (as for the UMAP). **c**, Left, UMAP of vasa recta endothelial cells (L4 kidney, 10x). The trajectory reflects the spatial cell continuum along the vasa recta descending limb (VR D, 79–80) and the ascending limb (VR A, 81–82). Right, heatmap showing the relative expression of vasa recta markers along the trajectory. Note the marker transition from artery–arteriole (GJA5^+^) to capillary (CA4^+^) to vein (ACKR1^+^). **d**, Top, UMAP of male germ cells (L4 testis, 10x) corresponding to the spermatogenesis trajectory from stem cells (spermatogonia, 250) to late spermatids (256). Bottom, heatmap showing the relative expression of spermatogenesis markers along the trajectory (spermatogonium panel enlarged for resolution). **e**, Top left, UMAP of myeloid cells (L2 bone, bone marrow, 10x) corresponding to two haematopoiesis trajectories. One begins with haematopoietic precursor cells (169) and bifurcates at granulocyte–monocyte progenitors (181) into the neutrophil lineage (175–176) and the monocyte–macrophage lineage (182–184 and 187). The other connects erythroid progenitor and lineage cells (172–174) with a fraction of megakaryocyte progenitors nearby (170). Top right and bottom, heatmaps showing the relative expression of haematopoiesis markers along trajectories (neutrophils uniformly subsampled). Symbols in brackets indicate the description of genes identified by NCBI as loci: [*DMC1*], *LOC105858542*; [*HSPA1L*], *LOC105858168*; [*CD14*], *LOC105862649*; [*HBB*], *LOC105883507*; and [*HBA2*], *LOC105856254*. See also Extended Data Fig. 2. Source Data

The identified cell types in each organ are shown in Fig. 1e and Supplementary Fig. 1 along with organ-specific dendrograms (Fig. 2a and Supplementary Fig. 2). For example, the 31 cell types in limb muscle are distributed across endothelial (7 types), stromal (10 types) and immune (14 types) compartments. Among the stromal cells, in addition to fast and slow myocytes and tendon cells, we found adipocytes. Fatty infiltrates are rarely seen in murine skeletal muscle^20^ but are common in aged human muscle^21^, which suggests that lemurs could model fatty infiltration of muscle during human ageing. We also identified putative stem and progenitor cell populations: *MYF5*-expressing and *PAX7*-expressing muscle stem cells, and *PDGFRA*-expressing and *THY1*-expressing fibroadipogenic progenitors. In a companion paper^22^, we used selectively expressed markers to purify and functionally characterize these putative stem and progenitor populations, and we showed that they exhibit many characteristics that are more similar to their human stem cell counterparts than those of mice.

From blood, we identified lemur cognates of all major human and mouse immune cell types. In the lymphoid lineage, these included B cells, plasma cells, CD4^+^ and CD8^+^ T cells, natural killer (NK) cells, natural killer T (NKT) cells and innate lymphoid cells. In the myeloid lineage, these included erythrocytes, platelets, monocytes, macrophages, conventional and plasmacytoid dendritic cells (DCs), neutrophils, basophils and even the rare and fragile eosinophils (Fig. 1e). From bone, bone marrow and other haematopoietic and lymphoid tissues, we identified presumptive progenitors, including haematopoietic precursors, and progenitors of erythrocytes, megakaryocytes and granulocyte–monocytes as well as putative adipogenic and osteogenic progenitors. However, certain immune cell subtypes in humans and/or mice were not identified in lemurs. For example, despite large numbers (>9,000) captured in multiple tissues, lemur monocytes formed a single cluster in most tissues that could not be resolved into classical and non-classical monocytes based on markers used to distinguish the two cell types in humans (CD14 and CD16) or mice (CCR2, CX3CR1, LY6C1 and LY6C2, which has no primate orthologue)^23^. These markers were either not annotated in the lemur genome or not differentially expressed (Supplementary Table 1), which may be due to limitations in annotations of the current genome or to unique lemur biology. Similarly, conventional DCs could not be divided into type 1 and type 2 subtypes characteristic of humans and mice. Conversely, other DC molecular types were found (for example, FLT3^+^IGSF6^+^ DCs) that had no apparent human or mouse cognates. The broad spectrum of developing and mature immune cells across the body enabled us to reconstruct the early stages of haematopoietic development (see below), and the dispersal of immune cells throughout the body in health and disease (described in an accompanying paper^1^).

## Molecular gradients of cell identity

The majority of profiled lemur cells could be computationally separated into discrete clusters of cells with similar expression profiles. However, we found many examples in which cells formed a continuous gradient of gene expression profiles, which indicated a gradual transition from one molecular identity to another. Some of these transitions reflected a spatial gradient in a tissue, whereas others corresponded to a temporal gradient of a developmental process or induction of a physiological or pathological cell state.

**Extended Data Fig. 2:**
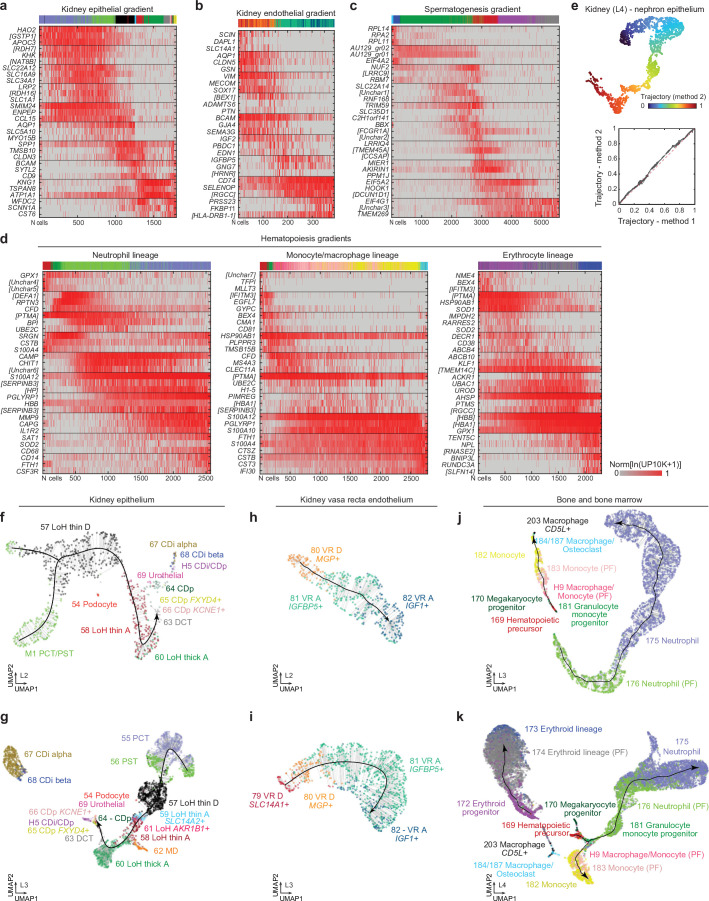
Heatmaps of DEGs along molecular gradients and UMAPs of cell trajectories for additional individuals. **a-d**. Heat maps showing relative expression of top DEGs along each trajectory in Fig. 2. Expression is normalized to the maximal value (99.5 percentile) for each gene across all cells in the trajectory. Genes shown are top three DEGs from each of the detected trajectory-dependent expression patterns described in Methods. Cells are ordered left to right by their trajectory coordinates (Ncells), and their cell type designations are indicated by colors in top bar (as in UMAPs of Fig. 2). **e**. (Top) UMAP of kidney epithelial cells as in Fig. 2b color-coded by molecular trajectory coordinates calculated using algorithm Slingshot algorithm. (Bottom) Comparison of cell trajectory coordinates assigned by two independent methods: Method 1, in-house algorithm, and Method 2, Slingshot. Red dashed line, values for perfect 1-1 correlation. **f-k**. UMAPs and detected molecular trajectories of cells from the indicated tissues and compartments as in Fig. 2, but from other lemurs as indicated at bottom left of each UMAP. “thin D, thin descending; thin A, thin ascending; thick A, thick ascending”. [], description of genes identified by NCBI as a loci: *[BEX1], LOC105884179; [CCSAP], LOC105877478; [DCUN1D1], LOC105862715; [DEFA1], LOC105881500; [FCGR1A], LOC105865511; [GSTP1], LOC105867419; [HBA1], LOC105856255; [HBB], LOC105883507; [HP], LOC105859005; [HLA-DRB1-1], LOC105872012; [HRNR], LOC105859819; [IFITM3], LOC105874071; [LRRC9], LOC105882927; [NAT8B], LOC105884612; [PTMA], LOC105880511; [SERPINB3], LOC105883741; [SERPINB3], LOC105876721; [TMEM45A], LOC105859377; [TMEM14C], LOC105865212; [RGCC], LOC105871594; [RDH7], LOC105865610; [RDH16], LOC105865617; [RNASE2], LOC105864771; [uncharacterized 1], LOC105876678; [uncharacterized 2], LOC105873147; [uncharacterized 3], LOC105862290; [uncharacterized 4], LOC105858108; [uncharacterized 5], LOC105880776; [uncharacterized 6], LOC105881161; [uncharacterized 7], LOC105871650*. Source Data

The kidney provided a marked example of cell-type gradients that corresponded to the spatial organization of the nephron. Most notable among the approximately 14,800 profiled kidney cells were the many epithelial cells that formed a long continuous gradient of molecular identity (Fig. 2b and Extended Data Fig. 2a,e–g). Through the use of canonical renal markers, we identified that these epithelial cells corresponded to the spatial distribution of the cell types along the lemur nephron, starting from proximal convoluted tubule cells, through the loop of Henle and ending with principal cells of the collecting duct. We also found a distinct gradient of endothelial cells with arterial markers (GJA5^+^ and BMX^+^) expressed at one end and venous markers (ACKR1^+^ and VCAM1^+^) at the other, with capillary markers (CA4^+^) in between (Fig. 2c and Extended Data Fig. 2b,h,i). This gradient probably comprises the vasa recta, a network of blood vessels intermingled with the loop of Henle, because it expressed specific vasa recta markers (for example, AQP1 and SLC14A1 for vasa recta descending arterioles) and was molecularly distinct from glomerular endothelial cells (EDH3^+^), other capillary endothelial cells (possibly peritubular) and lymphatic endothelial cells (CCL21^+^). This deep molecular map of the lemur nephron revealed region-specific functions such as hormonal signalling^24^.

Other observed gradients represented cell lineages, such as the development of haematopoietic progenitors in the bone marrow (Fig. 2e and Extended Data Fig. 2d,j,k). One gradient showed the bifurcation of granulocyte–monocyte progenitors into the monocyte–macrophage and neutrophil lineages, whereas the other represented the maturation of the erythroid lineage. Some common but more subtle gradients marked the differentiation of basal epithelial cells into their corresponding mature epithelial cell types in the skin, tongue, small intestine, colon and bladder along the suprabasal–luminal axis of each organ.

The most notable developmental gradient was in the male gonad. Among the approximately 6,500 testis cells profiled, all the germ cells formed a single continuous gradient (Fig. 2d and Extended Data Fig. 2c). The gradually changing expression levels of genes across the continuum enabled us to reconstruct the full gene expression program of lemur spermatogenesis. We assigned seven canonical stages from stem cells (spermatogonia) to mature spermatids using orthologues of stage-specific markers from humans and mice (Fig. 2d). In addition to the essential role of male germ cell differentiation in reproduction, the lemur spermatogenesis program is of particular interest because such programs are rapidly evolving during primate speciation (see below)^25^.

## Previously unknown cell types

**Fig. 3:**
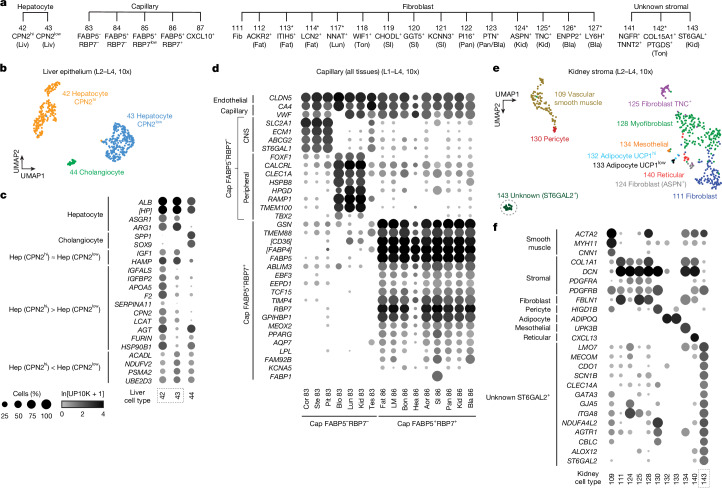
Previously unknown and understudied molecular cell types. **a**–**f**, Cell-type dendrogram (**a**), UMAPs (**b**,**e**) and gene expression dot plots (**c**,**d**,**f**) of examples of previously unknown molecular types identified in the atlas, including two hepatocyte types (**b**,**c**), FABP5^+^RBP7^+^ capillary cells (**d**) and an unknown kidney stromal cell (designation 143; unknown ST6GAL2^+^), which may be mesangial cells (**e**,**f**). In **a**, asterisks indicate cell types profiled in only one individual. Labels in parentheses indicate the tissue abbreviation for molecular types found in ≤3 tissues. Dot plots (**c**,**d**,**f**) show mean expression (ln(UMI_*g*_/UMI_total_ × 1 × 10^4^ + 1), abbreviated as ln(UP10K + 1) in dot heatmaps) and the percentage of cells expressing (dot size) the indicated cell-type markers and selected DEGs. In **d**, note that FABP5^+^RBP7^+^ capillary cells seem to be specialized for energy storage because they were found in high-energy-demand tissues (for example, heart, limb muscle and kidney) and enriched in genes for fatty acid uptake and binding (for example, *RBP7*, *FABP1* and *FABP5*), as well as the transcription factors *MEOX2* and *TCF15*, as in humans and mice^30,43^. Note also DEGs distinguishing FABP5^–^RBP7^–^ capillary cells from the CNS (cortex, brainstem and pituitary) versus peripheral tissues (blood, lung and kidney), which indicates tissue specialization of capillaries^24^. Cell types are indicated by tissue and designation number. Bla, Bladder; Blo, blood; Bon, bone; BM, bone marrow; Cap, capillary; Col, colon; Cor, brain cortex; Hep, hepatocyte; Pit, pituitary; Kid, kidney; Liv, liver; Lun, lung; MG, mammary gland; Pan, pancreas; SI, small intestine; Ski, skin; Spl, spleen; Ste, brainstem; Ton, tongue; Tra, trachea; Ute, uterus. Symbols in brackets indicate the description of genes identified by NCBI as loci: [*HP*], *LOC105859005*; [*FABP4-like*], *LOC105857591*; and [*CD36*], *LOC105879342*. See also Extended Data Figs. 3–4 and Supplementary Fig. 3. Source Data

We were able to assign provisional identities to most of the cell populations on the basis of expression of orthologues of canonical cell markers from humans and mice. However, there were dozens of cases in which more than one cluster in a tissue expressed markers of the same cell type, and their separation could not be attributed to technical differences (Fig. 3a and Supplementary Fig. 1). In some cases, these are probably different states of the same cell type because the DEGs included proliferation markers (for example, MKI67 and TOP2A), which indicated a proliferative state, or differentiation markers, which indicated a developmental state. In most cases, however, the additional clusters seemed to represent previously unknown or undercharacterized cell types or subtypes. Such clusters were identified in all compartments, except the germline, and in most profiled organs (Supplementary Fig. 1).

Fibroblast subtypes were particularly diverse, with multiple molecular types identified in many organs (Fig. 3a and Supplementary Fig. 1). Most seemed to be organ-specific, with little co-clustering across organs and without known parallels in humans or mice. Similarly, macrophages formed multiple distinct clusters in many tissues^1^ (Supplementary Fig. 1), and T cells and NK cells also exhibited diversity not readily harmonized with classical T cell subtypes of humans and mice^26^.

**Extended Data Fig. 3:**
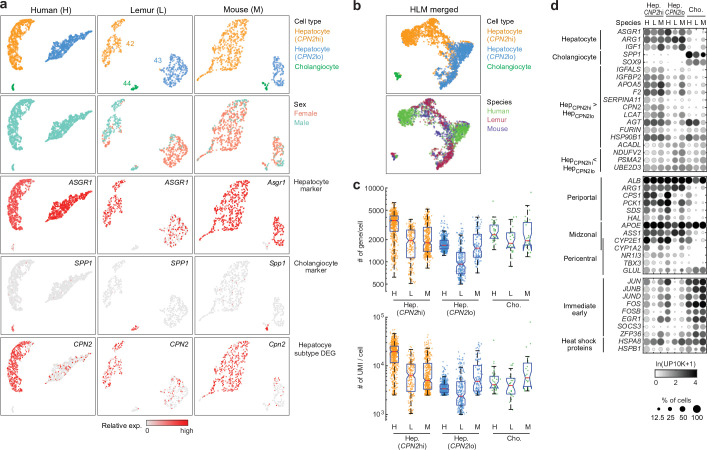
Hepatocyte molecular subtypes across human, lemur, and mouse. **a**. UMAP of liver hepatocytes and cholangiocytes, separately for human (left), lemur (middle), and mouse (right). Top to bottom: cells colored by cell type annotation, by sex of the animal, and by heatmap showing relative expression of a hepatocyte marker (*ASGR1*), a cholangiocyte marker (*SPP1*), and a hepatocyte subtype DEG (*CPN2*). Note that in the lemur atlas *CPN2*^hi^ and *CPN2*^low^ hepatocytes are given the designations hepatocyte (*APOB*+) and hepatocyte (*PHYH*+), respectively. **b**. Species-integrated UMAP of liver hepatocytes and cholangiocytes, with cells colored by cell type annotation (top) and species (bottom). **c**. Box and whisker plots of the number of genes (top) and UMIs (bottom) detected per cell for each cell type indicated. H, Human; L, lemur; M, mouse. **d**. Dot plot comparing mean expression (ln(UP10K+1), dot heamap) and percent of cells (dot size) expressing indicated genes in the two hepatocyte molecular subtypes and cholangiocytes across human, lemur and mouse. Genes shown are (top to bottom): one-to-one orthologues of hepatocyte and cholangiocyte markers, DEGs between the two hepatocyte subtypes, liver zonation markers^27^, and cell stress markers including immediate-early genes and heat shock proteins (labeled with the respective human gene symbol). Note that in all three species, hepatocyte molecular subtypes do not differ significantly in expression of zonation markers or cell stress markers. Source Data

The epithelial diversity detected included unexpected molecular subtypes of pancreatic acinar and ductal cells, kidney collecting duct principal cells, intestinal enterocytes (Supplementary Fig. 1) and hepatocytes that may serve specialized functions (Fig. 3a–c and Extended Data Fig. 3a–d). For example, CPN2^hi^ hepatocytes expressed high levels of genes that encode many classical liver-secreted proteins (for example, *IGFALS* and *APOA5*) and their processing enzymes (for example, *FURIN*) as well as additional hormones and receptors^24^. By contrast, CPN2^low^ hepatocytes expressed high levels of several mitochondrial metabolic genes (for example, *ACADL* and *NDUFV2*) and genes involved in proteasome-mediated protein degradation (for example, *UBE2D3* and *PSMA2*) (Fig. 3b,c and Extended Data Fig. 3d). The two types do not correspond to the known zonal heterogeneity of human and mouse hepatocytes^27^, but CPN2^hi^ hepatocytes expressed more transcripts and genes than CPN2^low^ hepatocytes (Extended Data Fig. 3c-d). Therefore, CPN2^hi^ cells could correspond to the larger, polyploid hepatocytes and CPN2^low^ cells to the smaller, diploid hepatocytes^28^. We identified a similar molecular distinction among hepatocytes in mouse^13^ and human^29^ liver scRNA-seq datasets (Extended Data Fig. 3a–d), which suggests that these subtypes are evolutionarily conserved. Likewise, we found potentially significant molecular diversity among endothelial cells, such as the FABP5^+^RBP7^+^ capillary subtype, which is apparently specialized for energy storage^30^. We also identified a CXCL10^+^ capillary subtype in an interferon-activated (for example, *GBP1* and *IFIT3*) inflammatory state^1,31^ and lymphatic subtypes (CCL21^+^ and CCDC80^+^) (Fig. 3a,d and Supplementary Fig. 1), which perhaps represent different lymphatic cell types in peripheral vessels and in lining lymph node sinuses^32^.

**Extended Data Fig. 4:**
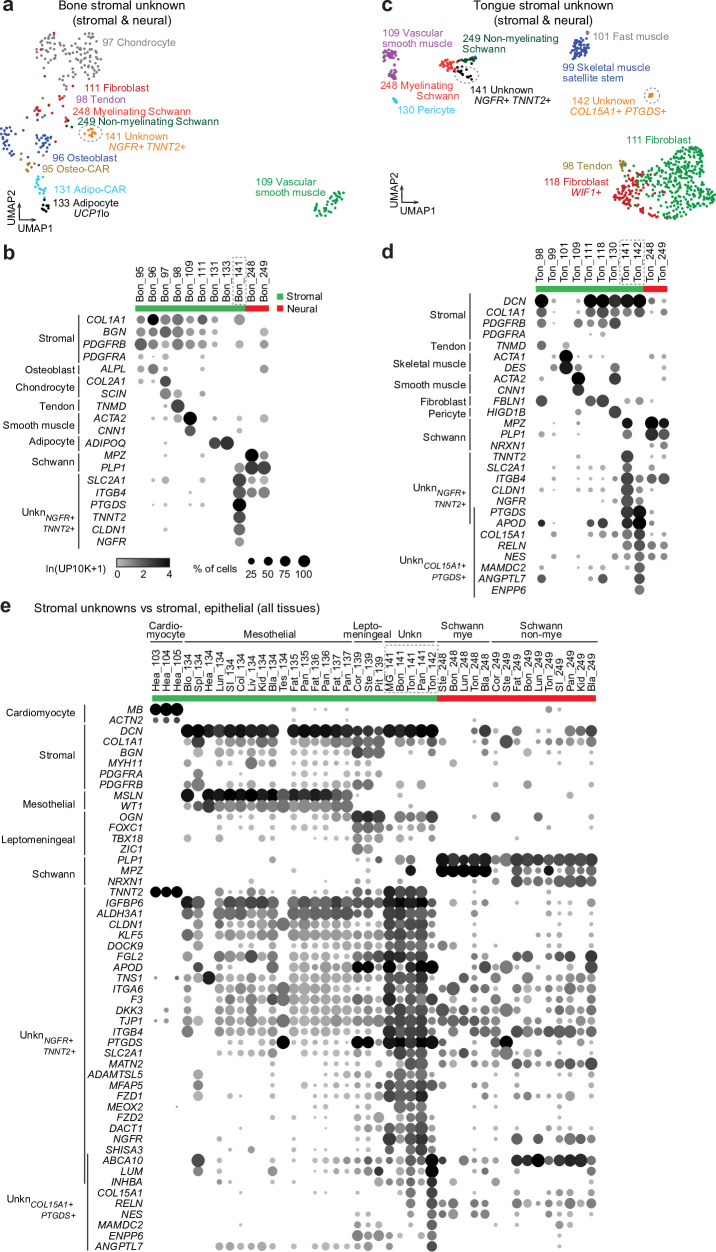
UMAP and differential expressed genes of previously unknown cell types. UMAPs (**a, c**) of indicated tissue compartments (integrated across individuals by FIRM, colored by molecular cell types) with unknown molecular type(s) highlighted (dashed circles), and corresponding dot plots (**b, d**) of mean expression (ln(UP10K + 1), dot heatmap; percent of cells expressing, dot size) of selected compartment and cell type marker genes as well as DEGs in the unknown cell type (dashed box) vs. other cell types in the dot plot. Cell types in dot plots are indicated by tissue_cell type designation number (for example, Bon_95) and compartment is indicated by color of bar beneath it. **a,b**. Bone stromal and neural cells (L2 and L4, 10x) with unknown stromal cell type (*NGFR*+*TNNT2*+, #141). **c,d**. Tongue stromal and neural cells (L2 and L4, 10x) with unknown stromal cell types (*NGFR*+*TNNT2*+, #141) and (*COL15A1*+*PTGDS*+, #142). **e**. Combined dot plot as above (b,d) showing expression of indicated marker genes and DEGs in the unknown stromal populations above (#141, #142) and the related unknown stromal population (*NGFR*+*TNNT2*+, #141) present in mammary gland and pancreas. Also plotted are cardiomyocytes, mesothelial, leptomeningeal, and Schwann cells from all tissues (L1-L4, 10x), which express high levels of the DEGs of #141 and 142. See also Supplementary Fig. 3. Source Data

For 8 (around 1%) out of the 768 molecular cell types, we were unable to assign a specific identity (Extended Data Fig. 4 and Supplementary Figs. 1 and 3). These cells included stromal types in tongue (cell-type designation 142; Extended Data Fig. 4c–e) and kidney (143; Fig. 3a,e,f and Supplementary Fig. 3a,b), and epithelial types in fat (15; Supplementary Fig. 3c–e) and blood (16; Supplementary Fig. 3f–h). The remaining four stromal types, from bone, mammary gland, pancreas and tongue, were given the same designation (141, ‘unknown stromal NGFR^+^TNNT2^+^’) because they shared similar transcriptomic profiles, including a notably high expression of *TNNT2*, which encodes cardiac troponin T, a contractile component and specific cardiac myocyte marker and clinical marker of myocardial infarction^33^ (Extended Data Fig. 4a–e). Some of the unknown cell types (15, 141 and 142) resembled mesothelial cells, with many of their DEGs enriched in mesothelial cells (Extended Data Fig. 4e). However, some of the DEGs were also characteristic of other cell types (for example, leptomeningeal and Schwann cells for designations 141 and 142, respectively, and urothelial cells for 15), and in the global cell-type comparison (see below), they did not closely localize with any of these. The unknown kidney stromal type (143) might be mesangial cells because they expressed at high levels two genes (*LMO7* and *ITGA8*) enriched in mouse mesangial cells^34^ (Fig. 3e,f and Supplementary Fig. 3a,b). The most perplexing unknown was the epithelial population from blood (16) of one individual (lemur L2). Its distinct gene signature included genes expressed by brain ependymal cells, astrocytes and oligodendrocytes (for example, *SOX2* and *UBR2*); however, this population did not express canonical markers of these cell types (Supplementary Fig. 3f–h).

Some of the cell types and subtypes described above may represent previously unrecognized or sparsely characterized cell states, including pathological states^1^. Others may be newly described cell types or subtypes, including ones unique to lemur or primates. It will be important to characterize for each of these cell types their functions and evolutionary conservation.

## Global comparison of cell types

**Fig. 4:**
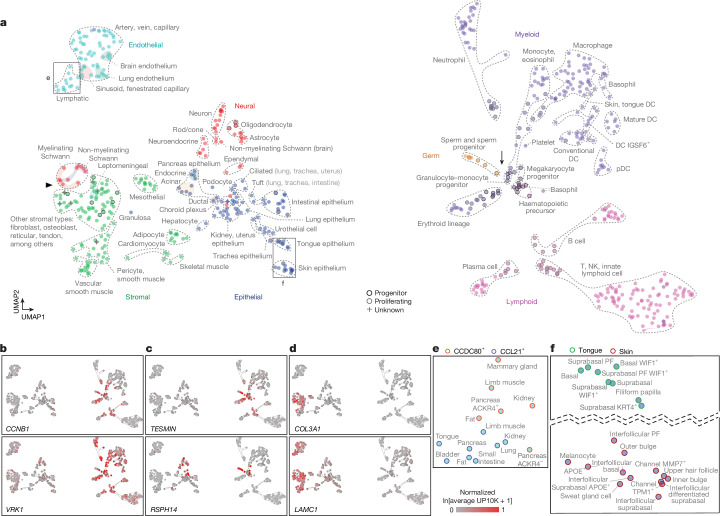
Relationships of molecular cell types across the lemur atlas. **a**, UMAP of molecular cell types (dots) in the atlas based on their mean transcriptomic profiles (L1–L4, 10x). Dot fill colour, tissue compartment; black or grey outline, progenitor or proliferating cell types, respectively. Dashed contours, biologically related cell types. Arrow and arrowhead, unexpected molecular convergence highlighted in **b–d**; boxed regions are shown in **e** and **f**. **b**–**d**, UMAPs as in **a** showing relative expression level heatmaps of the indicated DEGs for the following comparisons: immune progenitor and proliferating cells and germ cells versus cell types in other compartments (**b**; additional genes in Extended Data Fig. 5j); immune progenitor and proliferating cells and germ cells versus proliferating cells in other compartments (**c**; additional genes in Extended Data Fig. 5j); and Schwann and stromal cells versus non-Schwann cell types in the neural compartment (**d**; additional DEGs include *SOCS3*, *COL1A2*, *COL5A2*, *ID3*, *ID1*, *CDC42EP5*, *MMP2*, *TGFBR2*, *CCN1* and *TBX3*). **e**,**f**, Close-up of the boxed regions in **a** showing segregation of two types of lymphatic cell independent of tissue of origin (**e**), versus tissue-specific segregation of skin and tongue epithelial cell types despite their relatively similar functions in both tissues (for example, basal cells) (**f**). Source Data

**Extended Data Fig. 5:**
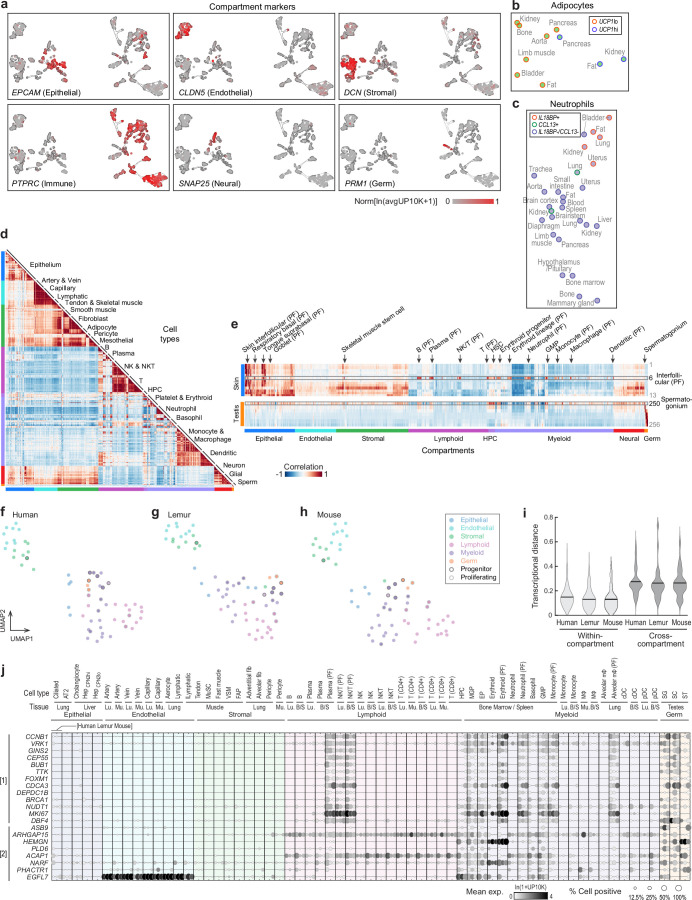
Relationships of molecular cell types within and across compartments in human, lemur, and mouse. **a**. Cell type UMAP as in Fig. 4a overlaid with relative expression level (as heat maps) of example tissue compartment markers indicated. **b,c**. Close up of portions of UMAP in Fig. 4a showing segregation of two types of adipocytes (**b**) and three types of neutrophils (**c**) independent of their tissue of origin. **d**. Heat maps of pairwise Pearson’s correlation coefficients between the transcriptomic profiles of each of the 749 molecular cell types in atlas (10x and SS2 datasets, excluding cardiac cells), calculated from principal component values of FIRM-integrated UMAP (Extended Data Fig. 1c) averaged across all cells of each type. Cell types ordered by compartment, then cell type designation/number, and then tissue. An interactive version of this map is available at https://tabula-microcebus.ds.czbiohub.org/heatmaps. **e**. Close up of heat map from panel d showing pairwise correlations between skin epithelial cells and all other cell types (top), and between testis germ cells and all other cell types (bottom). Note proliferating skin interfollicular suprabasal cells show high correlation with other proliferating and progenitor cell types across all compartments in atlas. Spermatogonia also show high correlation with proliferating cell types in the atlas, and especially with hematopoietic progenitor cells. **f-h**. UMAPs of the 63 orthologous cell types (see evolutionary comparisons) clustered separately for human (**f**), lemur (**g**), and mouse (**h**). Dot color, cell types compartment. Note the close clustering of germline progenitors with immune progenitors, as shown for lemur in Fig. 4a, is consistent across all three species. **i**. Violin plot showing distribution of transcriptomic distances between pairs of cell types (see Methods) within the same compartment and across different compartments for the 63 orthologous cell types, separated by species. **j**. Dot plot showing mean expression across the 63 human, lemur and mouse orthologous cell types of DEGs detected by comparing the lemur immune progenitor/proliferating cells and germ cells vs. cell types in other compartments [1], or vs. proliferating cells in other compartments [2], as in Fig. 4b,c. Figure format as in Fig. 5i. See also Supplementary Fig. 4. Source Data

We examined the molecular relationships of all cell types in the atlas using simplified UMAPs, in which each molecular type was condensed into a single data point representing the mean expression value of all cells of that type (pseudo-bulk expression profile) (Fig. 4a). We also calculated pairwise correlation coefficients of all cell-type pseudo-bulk expression profiles and displayed them in a large (around 750 × 750) matrix (Extended Data Fig. 5d,e). Both approaches revealed global patterns of similarity as well as unexpected molecular convergence of seemingly distantly related cell types.

Molecular cell types in the same tissue compartment generally showed the most closely related expression profiles, even those from different organs (Extended Data Fig. 5a). This was also found for human and mouse data (Extended Data Fig. 5f–i). Endothelial cell types across the body formed the most coherent compartment. Next was the neural compartment, including CNS glial cells, which were surprisingly similar to neurons. Immune cell types were the most divergent, particularly between lymphoid and myeloid populations. However, the global analyses also identified a few exceptions: cell types that were more closely related to cell types in another compartment than to those in their own compartment (Fig. 4a–d). Some of these cross-compartment similarities were predictable. For example, neuroepithelial cells of the airway (neuroendocrine cells) and gut (enteroendocrine cells) were more closely related to neurons and pituitary neuroendocrine cells than to most other epithelial cell types.

Other identified cross-compartment similarities were unexpected. The most notable was that male germ cells (spermatogonia) were more closely related to immune progenitor cells than to any other cells in the atlas, including progenitors and proliferating cells of other compartments (Fig. 4a and Extended Data Fig. 5e). Similar convergence of spermatogonia and immune progenitors was observed for human and mouse data (Extended Data Fig. 5f–j). Spermatogonia and haematopoietic progenitors shared enriched expression of specific cell cycle genes, particularly M phase genes (for example, *CCNB1* and *VRK1*) (Fig. 4b and Extended Data Fig. 5j), which indicated similarity in their mitotic machinery. Compared with progenitors and proliferating cells of other compartments, spermatogonia and immune progenitors also shared selective expression of non-cell-cycle genes (for example, *TESMIN* and *RSPH14*), many that are similarly expressed in humans and mice (Fig. 4c and Extended Data Fig. 5j). This result suggests that these genes have a common and potentially conserved role in the regulation of stemness of immune and sperm progenitors.

Another example of cross-compartment similarity was myelinating and non-myelinating Schwann cells (peripheral glia), which segregated with stromal cells and away from central glia (for example, oligodendrocytes) (Fig. 4a). Differential expression analysis identified genes enriched in stromal and Schwann cells but not in other neural compartment cells (for example, *COL3A1*, *LAMC1* and *SOCS3*) (Fig. 4d) and a complementary set enriched in neurons and central glia but not Schwann cells (for example, *OMG*, *GPR137C* and *TCEAL3*). Many of the genes expressed in both Schwann and stromal cells were components or regulators of the extracellular matrix, which suggests that peripheral glia function with surrounding stromal cells in matrix remodelling.

Within a compartment, cells of the same designated type or subtype were generally closely clustered despite their different tissue origins (Fig. 4e and Extended Data Fig. 5b,c). The exception was epithelial cell types, which were highly tissue-specific and generally clustered with other epithelial cells from the same organ (for example, skin basal and suprabasal cell types clustered separately from those of tongue) (Fig. 4f). An example of an initially perplexing cross-organ similarity cluster was a distinctive population of epithelial cells from the lung of one individual (lemur L2), which clustered closely with uterine epithelial cells. However, these were subsequently found to be lung metastases of a uterine endometrial cancer^1^.

## Gene expression evolution in primates

**Fig. 5:**
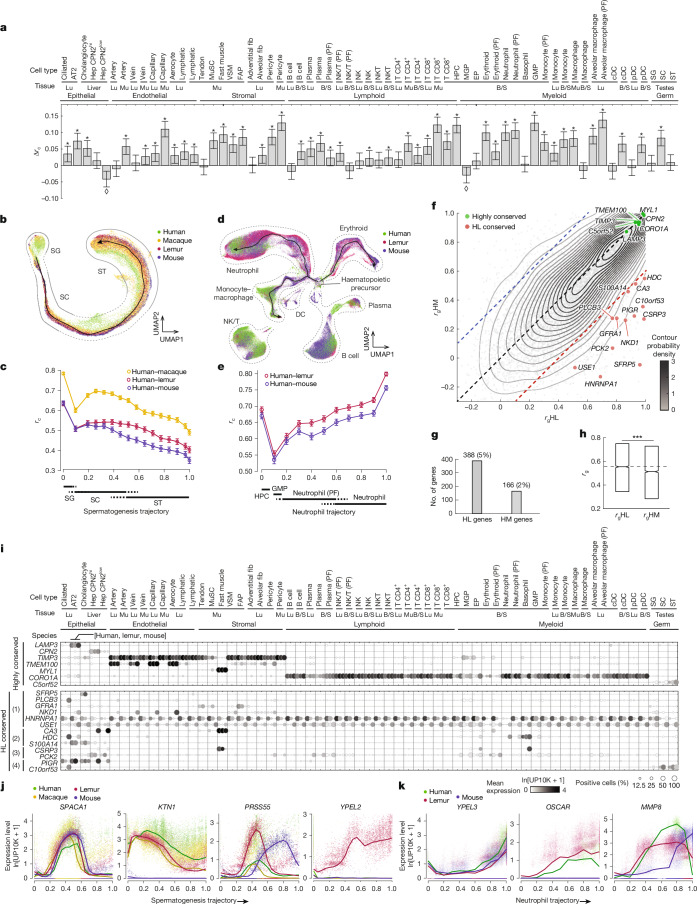
Evolutionary comparison of cell types and gene expression patterns. **a**, Bar plot showing the lemur-over-mouse advantage for modelling the human cell-type transcriptome for 63 cell types. Δ*r*_c_ = *r*_c_HL – *r*_c_HM, with *r*_c_ (transcriptomic correlation coefficient, one-to-one-to-one orthologous genes), *r*_c_HL (human-to-lemur), *r*_c_HM (human-to-mouse). *P* < 0.05, asterisks indicate right-tailed *t*-test, diamonds indicate left-tailed *t*-tests (exact values are provided in the source data). Error bar, 95% confidence interval. **b**,**c**, Species-integrated UMAP (coloured by species) of spermatogenesis (**b**; black curve, maturation trajectory; contours, related cell types) and transcriptomic correlation coefficients to the human profile (**c**; yellow, human–macaque (*r*_c_HMa); red, human–lemur (*r*_c_HL); blue, human–mouse (*r*_c_HM)). Error bar, 95% confidence interval. Human–macaque similarity may be overestimated because transcriptomic data are from the same study using drop-seq^35^, whereas lemur (this study) and mouse^36^ data are from independent studies using the 10x method. **d**,**e**, Species-integrated UMAP (**d**) and cell correlation coefficients (**e**) as in **b**,**c** but for haematopoiesis trajectories (bone marrow and spleen immune cells). Neutrophil trajectory (**e**), other immune lineages (Extended Data Fig. 8e,f). **f**–**h**, Comparison (correlation coefficients, *r*_g_, one-to-one orthologous genes) of gene expression patterns across 63 cell types (**a**) between human–lemur (*r*_g_HL) versus human–mouse (*r*_g_HM), shown as a scatter plot (**f**), bar plot (**g**) and boxplot (**h**). **f**, Grey dots, orthologous genes; green and red dots, highlighted highly conserved (green, *r*_g_HL and *r*_g_HM > 0.8) and human–lemur conserved (red) genes. Contours, probability density. Dashed lines, black, *r*_g_HL = *r*_g_HM; red, Δ*r*_g_,(*r*_g_HL – *r*_g_HM) threshold (Δ*r*_g_ > 0.4) for designated human–lemur-conserved and mouse–divergent genes (HL genes); blue, threshold (Δ*r*_g_ < −0.4) for designated human–mouse-conserved and lemur-divergent genes (HM genes)). **g**, Quantification of HL and HM genes. **h**, Comparison of *r*_g_HL and *r*_g_HM distributions. Median (central line), 25th (bottom) and 75th (top) percentiles; dashed line, *r*_g_HL median. *n* = 7,787. ***P = 3 × 10^–76^ (paired two-tailed *t*-test). **i**, Dot plots showing expression of example genes with highly conserved (top) and HL-conserved (bottom) patterns for the 63 cell types in **a**. Rows, orthologous genes (human symbol). Columns, cell-type expression displayed as human–lemur–mouse trios. Note different patterns of evolutionary expression rewiring among HL genes: (1) simple gain or loss of expression in primates versus mouse; (2) conserved expression in some cell types but expression expansion or contraction in other cell types in primates; (3) expression switches from one or more mouse cell types to different cell type (or types) in primates; and (4) complex expression rewiring (combinations of above). **j**,**k**, Scatter plots of expression along spermatogenesis (**j**) and neutrophil (**k**) trajectories of genes with highly conserved (*SPACA1* and *YPEL3*); primate-conserved and mouse-divergent (*KTN1* and *OSCAR*, mice lack expression; *PRSS55* and *MMP8*, mouse heterochronic expression), and lemur-specific (*YPEL2*) patterns. Points, individual cells, coloured by species; curves, moving average of expression along trajectory. B/S, bone marrow/spleen; EP, erythroid progenitor; Lu, lung; MGP, megakaryocyte progenitor; Mu, skeletal muscle; MuSC, skeletal muscle stem cell; SC, spermatocyte; SG, spermatogonium; ST, spermatid; VSM, vascular smooth muscle. See also Extended Data Figs. 6–10 and Supplementary Fig. 4. Source Data

**Extended Data Fig. 6:**
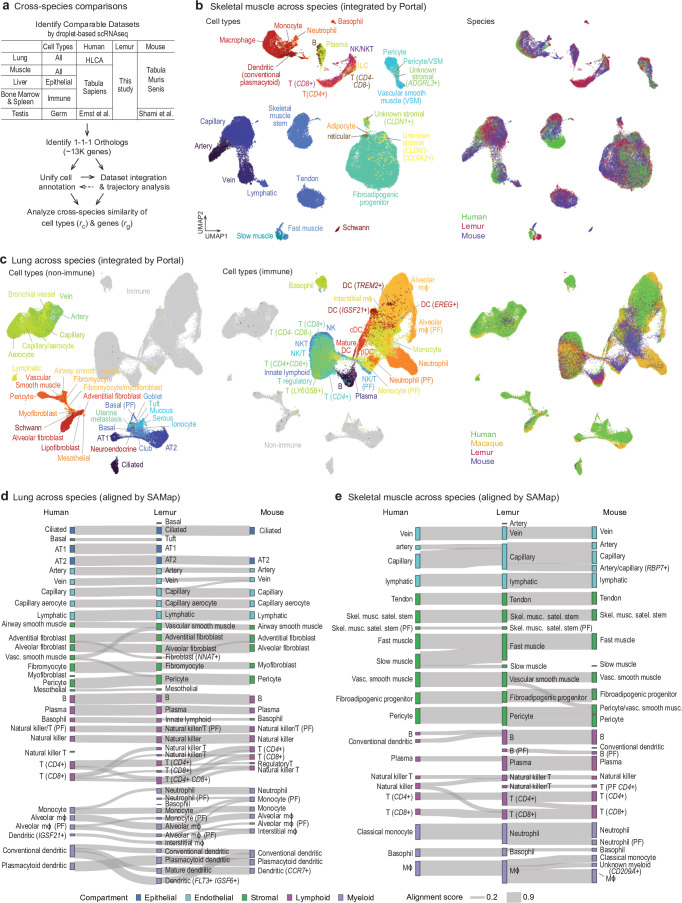
Molecular relationships of lung and skeletal muscle cell types across species. **a**. Overview of methodology for evolutionary cell type and gene comparison analysis using the indicated datasets. HLCA, Human lung cell atlas^14^. **b,c**. UMAPs of skeletal muscle (**b**) and lung (**c**) cells integrated across species by Portal based on the one-to-one gene orthologues, colored by cell type (**b**, left; **c**, non-immune cell types on left and immune in middle) and by species (**b** and **c**, right). **d,e**. Sankey plots showing the molecular relationship between human, lemur, and mouse cell types for lung (**d**) and skeletal muscle (**e**) as determined by SAMap algorithm^39^ (see Methods). Each cell type in lemur is connected (gray line) to the cell type(s) it maps to in human and mouse datasets; line thickness indicates molecular similarity score (0-1) between connected cell types. A cell type with no connecting lines indicates it did not map with similarity score > 0.1 to any cell type in the other species. Note that cell types of the same designation show higher similarity scores across species compared to with other cell types.

**Extended Data Fig. 7:**
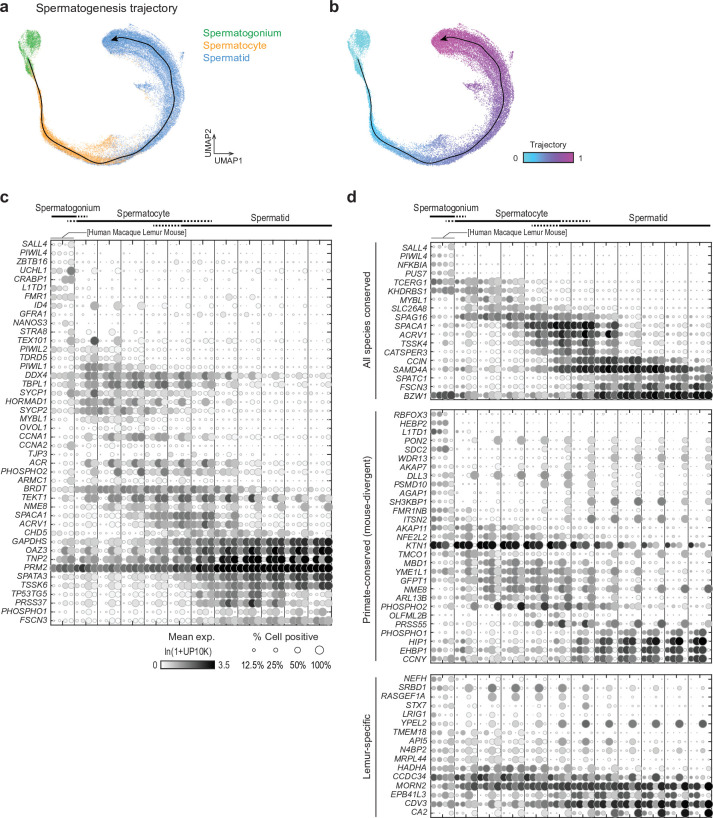
Evolutionary comparisons of spermatogenesis. **a,b**. UMAPs of male germ cells integrated across species as in Fig. 5b with cells colored by cell type/developmental stage (**a**) and pseudotime along the spermatogenesis trajectory (**b**). **c,d**. Dot plot showing mean expression along spermatogenesis trajectory for known spermatogenesis markers genes (**c**) and evolutionary conserved/divergent genes (**d**: top, all species-conserved; middle, primate-conserved and mouse divergent; bottom, lemur-specific). Rows are orthologous genes (indicated by the human gene symbol). Columns are cell types along trajectory, displayed as groups of four dots showing respective expression in the corresponding cell type of human, macaque, lemur, and mouse. Source Data

**Extended Data Fig. 8:**
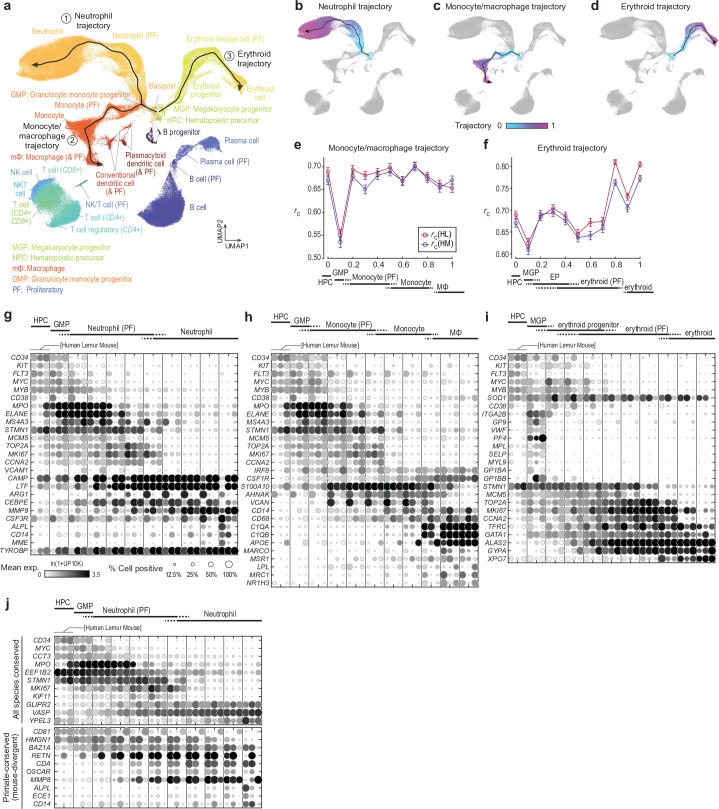
Evolutionary comparisons of hematopoiesis. **a-d**. UMAP of bone marrow and spleen immune cells integrated across species as in Fig. 5d, with cells colored by cell type (**a**) and pseudotime along the hematopoietic trajectories (**b**, neutrophil; **c**, monocyte/macrophage; **d**, erythroid). **e,f**. Correlation coefficients of human progenitor and mature immune cell transcriptomic profiles to those of lemur (*r*_c_HL) and mouse (*r*_c_HM) as shown in Fig. 5e but for monocyte/macrophage (**e**) and erythroid (**f**) trajectories. Note that *r*_c_HL is almost always greater than *r*_c_HM throughout the trajectories with the exception of the end of the monocyte/macrophage trajectory, likely confounded by different fractions of macrophages in each dataset. **g-j**. Dot plot showing mean expression along neutrophil (**g, j**), monocyte/macrophage (**h**), and erythroid (**i**) trajectories, for known markers genes (**g-i**) and evolutionary conserved/divergent genes (**j**: top, all species conserved; bottom, primate-conserved and mouse divergent). Rows are orthologous genes, indicated by their human gene symbols. Columns are cell types, displayed as trios of dots showing expression in the corresponding human, lemur, and mouse cell type. Source Data

To investigate how gene expression has changed during primate evolution, we compared the transcriptomic profiles of lemur cell types from six organs (lung, skeletal muscle, liver, testis, bone marrow and spleen) to the corresponding cell types of human, mouse and, where available, macaque, using mouse as the non-primate outgroup (Extended Data Fig. 6a and Supplementary Table 4). To ensure comparisons were made across truly orthologous cell types and to minimize technical artefacts, we focused on our own human^14,29^ and mouse datasets^13^ processed using the same scRNA-seq protocols by the same tissue-expert laboratories, but included additional testis data from mouse, human and rhesus macaque (*Macaca mulatta*)^35,36^ and lung data from crab-eating macaque (*Macaca fascicularis*)^37^. We re-clustered and re-annotated cells from these datasets using the same pipeline and marker genes used for the lemur. Orthologous cell-type assignments were then refined and verified by demonstrating co-clustering of corresponding cell types using the integration algorithm Portal^38^ (Fig. 5b,d and Extended Data Figs. 6b,c, 7a,b and 8a–d) and the cross-species data alignment algorithm SAMap^39^ (Extended Data Fig. 6d,e). We restricted analysis at the gene level to approximately 13,000 one-to-one-to-one gene orthologues across human, lemur and mouse (or about 12,000 when including macaque), which we curated by combining homology assignments from Ensembl and the National Center for Biotechnology Information (NCBI) (Supplementary Table 5). The above analysis identified and validated 63 orthologous cell types across human, lemur and mouse (18 with macaque), and continuous trajectories of developing haematopoietic and male germ cells.

**Extended Data Fig. 9:**
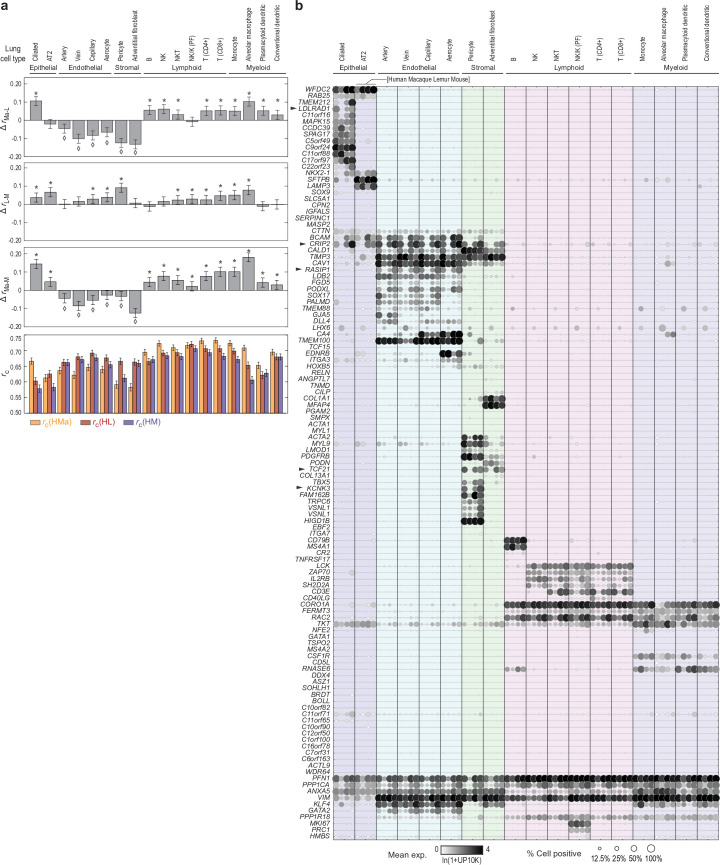
Evolutionary comparison of lung cell types across human, lemur, mouse, and macaque. **a**. Bar plots comparing differences (Δ*r*_c_) between human-to-macaque (*r*_c_HMa), human-to-lemur (*r*_c_HL) and human-to-mouse (*r*_c_HM) transcriptomic correlation coefficients (top to bottom: Δ*r*_c_Ma-L = *r*_c_HMa-*r*_c_HL, Δ*r*_c_L-M = *r*_c_HL-*r*_c_HM, Δ*r*_c_Ma-M = *r*_c_HMa-*r*_c_HM)) for each of the 18 orthologous lung cell types indicated. *p* < 0.05, * (right-tailed t-test), ◊(left-tailed). Bottom panel, correlation coefficient between each species. **b**. Dot plot showing mean expression of genes highly-conserved between human, lemur, and mouse (as in Fig. 5i and Supplementary Fig. 4a) in the 18 orthologous lung cell types across human, macaque, lemur, and mouse. Genes without an orthologue in macaque were excluded. Note that macaque cell types generally showed similar expression patterns to cognate cell types in the other three species, but with exceptions (notable examples indicated by arrowheads). Format as in Fig. 5i. Source Data

**Extended Data Fig. 10:**
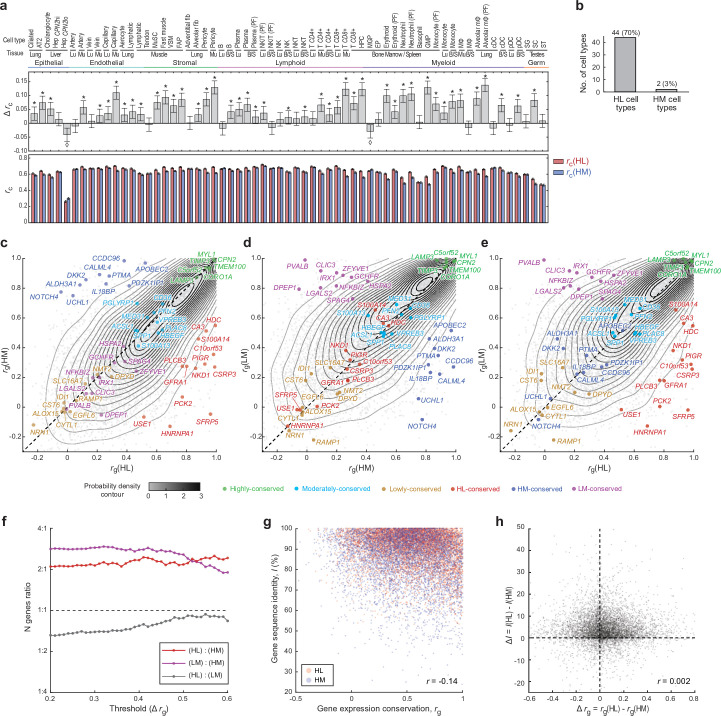
Evolutionary comparison of gene expression and sequence conservation. **a**. Expanded version of Fig. 5a including (bottom panel) comparison of transcriptomic correlation coefficient score between human and lemur (*r*_c_HL) and between human and mouse (*r*_c_HM) for each of 63 orthologous cell types. **b**. Bar graph quantifying the number of cell types in panel a that are more similar between human and lemur compared to human and mouse (Δ*r*_c_ > 0, where Δ*r*_c_ = *r*_c_HL-*r*_c_HM) and vice versa). **c-e**. Scatter plots comparing gene expression conservation patterns (correlation coefficients) between human and lemur (*r*_g_HL), human and mouse (*r*_g_HM), and lemur and mouse (*r*_g_LM) for each one-to-one orthologous gene, formatted as Fig. 5f and dot colors as in legend. Expression of the highlighted genes are shown in Fig. 5i and Supplementary Fig. 4. **f**. Ratio of HL, HM, and LM conserved genes detected at different Δ*r*_g_ thresholds. The number of HL and LM genes were consistently higher than (more than doubling) that of HM genes, whereas the number of HL and LM genes were more comparable, supporting the lemur as genetic intermediate between human and mouse. **g,h**. Scatter plots comparing gene expression conservation (*r*_g_) vs. gene sequence identity (*I*) between HL and HM one-to-one orthologues (**g**) and HL-HM differences in expression conservation (Δ*r*_g_) vs. gene sequence identity (Δ*I*) (**h**). Note the lack of positive correlation between measurements (Pearson’s *r* in g = −0.14 and in h = 0.002). Source Data

Comparison of the transcription profiles of the 63 orthologous cell types showed that transcriptional similarity (*r*_c_) across species ranged from 0.26–0.72 (0.63 ± 0.06, mean ± s.d.) (Extended Data Fig. 10a). Transcriptomic similarity of orthologous human and lemur cell types and trajectories was almost always greater than that of the corresponding human and mouse cell types and trajectories, as expected from the closer evolutionary relationship between humans and lemurs (Fig. 5a and Extended Data Fig. 10a,b). However, the magnitude of cell-type transcriptional differences across species, and the expected advantage of lemurs over mice in modelling the corresponding human cell type, differed by cell type and varied along the trajectories. This finding indicated that there were cell-type-specific rates of evolutionary diversification in their expression programs. For example, developing male germ cells showed decreasing cross-species similarity (*r*_c_) and increasing human–lemur to human–mouse differences (Δ*r*_c_) along the developmental trajectory (Fig. 5c), which implied that there was more rapid molecular evolution of the late stages of spermatogenesis among the species. By contrast, neutrophils showed increasing *r*_c_ and generally increasing Δ*r*_c_ as progenitors matured (Fig. 5e and Extended Data Fig. 8e,f). Comparisons with available transcriptomes of orthologous macaque cell types showed similar trends, with macaque cell types generally displaying greater transcriptional similarity to those of human than the other species (Extended Data Fig. 9a). However, there were exceptions. For several cell types, notably in the lung endothelial and stromal compartments, the lemur cell type better mimicked the human orthologue transcriptome than did the macaque (Extended Data Fig. 9a). This result suggested that there was a unique evolutionary adaptation in gene expression for these cell types in the macaque lineage. Thus, although the transcriptional similarity of orthologous cell types is generally consistent with expectations from phylogenetic relationships, individual cell types have transcriptionally diversified at different rates during primate evolution, some so much that they violate phylogenetic expectations.

To provide molecular insight into these cell-type specializations in primate evolution, we identified for each cell type the cell-type-selective genes for which expression was conserved across all species analysed, defining mammalian cell-type core gene expression programs. We also identified genes for which expression was conserved in primates but not mouse; these may contribute to primate-selective cell properties (Supplementary Table 6). Each cell type had dozens to hundreds (range 18–595, 174 ± 95 mean ± s.d.) of genes with a primate-selective expression pattern (*P* < 1 × 10^–5^ and >5-fold enriched or depleted in the primate lineage).

We also analysed the conservation of the global pattern of expression for each gene across the analysed cell types in human, lemur and mouse data (Fig. 5f–i, Extended Data Fig. 10c–f and Supplementary Table 7). Notably, only a small fraction of genes exhibited highly conserved expression patterns across all three species (11% at a *r*_g_ threshold of 0.8). These highly conserved genes were enriched for compartment-specific or cell-type-specific genes (for example, *LAMP3* in alveolar epithelial type 2 cells and *TIMP3* in endothelial and stromal compartments) and structural and regulatory genes of the cytoskeleton, cilia and extracellular matrix (for example, *ACTA1* and *MYL1* in the skeletal muscle) (Fig. 5f,i, Supplementary Table 8 and Supplementary Fig. 4a). Of note, multiple uncharacterized genes (for example, *C5orf52* and *C11orf65*) exhibited conserved expression in ciliated cells and/or male germ cells, which suggests that they may regulate specialized cytoskeletal features. Orthologous macaque genes were similarly expressed, with exceptions such as *KCNK3* (which encodes a potassium channel), a human pulmonary hypertension gene^40^, which was selectively expressed in lung pericytes (but not muscle pericytes) of humans, lemurs and mice, but not of macaques (Extended Data Fig. 9b and Supplementary Fig. 4a).

Most genes (89%) displayed evolutionarily divergent expression patterns (*r*_g_ < 0.8) (Fig. 5f,i and Supplementary Fig. 4b–e). Although expression pattern conservation was overall greater between humans and lemurs than between humans and mice (Fig. 5f–h and Supplementary Fig. 4b), there was a wide range in expression plasticity of individual genes, including 7% that showed extreme plasticity (*r*_g_ < 0.3) (Extended Data Fig. 10c–e and Supplementary Fig. 4f). Expression conservation did not correlate with coding sequence conservation (Extended Data Fig. 10g,h), which implied the presence of separate evolutionary diversification mechanisms or selective pressures for the expression control sequences and protein-coding sequences at each gene.

Figure 5f highlights genes (in red) for which the expression pattern was selectively conserved in the primates (human–lemur; HL genes) and therefore may contribute to primate-specific traits. Some showed simple gain (or loss) of expression in the primate lineage and involved a single cell type (for example, *SFRP5* and *PLCB3*), several types (for example, *GFRA1* and *NKD1*) or nearly all analysed types (for example, *HNRNPA1* and *USE1*) (Fig. 5i and Supplementary Fig. 4b). Others showed expression expansion (or contraction) into more (or fewer) primate cell types than in mouse (for example, *CA3* and *S100A14*), whereas some switched expression from one cell type to another between primates and mice (for example, *CSRP3* and *PCK2*). However, many HL genes displayed combinations of these types of differences from mice, which indicated the occurrence of complex evolutionary rewiring of their expression patterns (for example, *PIGR* and *C10orf53*). In many cases, such expression rewirings were to cell types in a different tissue compartment or organ (for example, *HDC*, *FGFR3*, *FIBIN* and *EFHD1*) (Fig. 5i, Supplementary Fig. 4b). Many HL genes (26%) are linked to human diseases (Supplementary Table 7). In a similar way, we identified lemur–mouse (LM) genes and human–mouse (HM) genes that may contribute to human-selective and lemur-selective biology, respectively (Extended Data Fig. 10c–e and Supplementary Fig. 4c,d).

We carried out a similar analysis of primate gene expression conservation along the spermatogenesis trajectory. Many genes that mark canonical spermatogenesis stages were similarly expressed in all four species (Fig. 5j and Extended Data Fig. 7c,d), which defined a conserved core program of mammalian spermatogenesis. However, we also identified primate-specific features of the program, including genes expressed only in primate spermatogenesis (for example, *KTN1* and *L1TD1*) (Fig. 5j and Extended Data Fig. 7d), along with dozens of primate-selective genes (orthologues identified in humans and lemurs but missing in mice) that showed enriched expression during spermatogenesis^1^. We also found genes expressed in all species during spermatogenesis but for which expression peaked at different times in primates and mice, which indicated heterochronic rewiring of the spermatogenesis program during primate evolution (for example, *PRSS55* and *PHOSPHO2*), in addition to genes for which spermatogenesis expression patterns were lemur-specific (for example, *YPEL2* and *CA2*) (Fig. 5j and Extended Data Fig. 7d). Such genes are of particular interest because of their potential role in the remarkable radiation of the Lemuroidea clade (>100 species, constituting nearly one-quarter of all primate species) and because several notable specializations have already been recognized for mouse lemurs including seasonal regulation of testes and the role of sperm competition in reproduction^25^. A similar comparison of haematopoietic trajectories showed generally conserved expression patterns across known marker genes and identified genes similarly expressed in the primates (human and lemur) but showed no expression or heterochronic changes in mouse haematopoiesis (for example, *Mmp8* and *Oscar* in the neutrophil lineage)^1^ (Fig. 5k and Extended Data Fig. 8g–j).

Extending such comparisons to all orthologous cell types and more broadly across phylogeny will provide insight into the selection pressures and molecular changes that underlie the evolutionary specializations of primate cell types. Even at this stage, the comparisons performed here suggest interesting biological hypotheses and identify many cell types and genes for which lemurs could provide a human modelling advantage over mice.

## Discussion

Our scRNA-seq and analytical pipeline defined over 750 mouse lemur molecular cell types and their expression profiles, including cognates of nearly all canonical human and mouse cell types in the 27 tissues and organs profiled. We also analysed stem and progenitor cells and their developmental programs for spermatogenesis, haematopoiesis and other adult tissues. Expert curation also uncovered dozens of previously unidentified or undercharacterized cell types, some of which seem to be conserved (for example, two types of hepatocytes), whereas others may be primate innovations.

By organizing cell types by organ, compartment and function, and then globally comparing their expression profiles, we defined the molecular relationships of cell types across the body. This analysis revealed global features such as high molecular similarity of cell types in some compartments (for example, endothelial) but marked divergence of cell types in others (for example, immune). Unexpected similarity of a few cell types across compartments, such as peripheral glia to stromal populations, were also revealed.

The atlas provides a broad cellular and molecular foundation for studying this model primate. Beyond defining and organizing lemur cell types, the atlas aids elucidation of their functions and enables molecular comparisons of lemur cell types to their homologues in other organisms, facilitating exploration of primate biology and evolution at cellular resolution. Our analyses revealed the many cell types (nearly all analysed) and expressed genes (many implicated in human diseases) for which the mouse lemur provides a human modelling advantage over mice and some even over macaque, as well as cases such as sperm with a multitude of primate and lemur innovations. The atlas also provides a new way of detecting unannotated genes, defining their structures and splicing, and elucidating organism-wide processes such as hormonal signalling, immune cell activation, and primate-specific physiology and diseases^1,24^.

Although the first steps in establishing a new model organism have traditionally been screens of mutants and the generation of a genetic map or reference genome, with technological advances and falling costs of scRNA-seq, the creation of a reference transcriptomic cell atlas can now also be prioritized. The strategy used here to create the mouse lemur atlas (opportunistic donor and systematic tissue collection; extensive clinical and histopathological metadata; broad and deep scRNA-seq, iterative cell clustering, integration and expert annotation; biological organization of cell types and comparisons across and between organisms) can be adapted to other emerging model organisms. Application of the strategy to a wide range of organisms^41,42^ will rapidly expand our cellular, genetic and molecular understanding of biology and disease that has been dominated for a half century by a small number of non-primate models.

## Methods

### Animal husbandry

All four grey mouse lemurs (*M.* *murinus*) included in this atlas originated from the closed captive breeding colony at the Muséum National d’Histoire Naturelle in Brunoy, France, and were transferred together to the University of Texas (Austin) in 2009 and then to Stanford University in 2015 and maintained for non-invasive phenotyping and genetic research as approved by the Stanford University Administrative Panel on Laboratory Animal Care (APLAC number 27439) and in accordance with the Guide for the Care and Use of Laboratory Animals, as previously detailed^15^. In brief, mouse lemurs were housed indoors in modified marmoset cages with multiple PVC perches and nest boxes in a facility credited by The Association for Assessment and Accreditation of Laboratory Animal Care in a temperature (23.3–24.4 °C) and light-controlled environment (daily 14:10 h and 10:14 h light–dark, alternating every 6 months to synchronize seasonal breeding behaviour and metabolic changes) and were fed ad libitum with fresh fruits and vegetables, crushed primate chow (Teklad Global 20% Protein Primate Diet, 2050, Envigo) and live insect larvae as enrichment items. Animals were socially housed in single-sex groups or individually housed owing to behavioural incompatibility or health management requirements. Health and welfare were monitored daily and clinical care was provided by the Veterinary Service Center, Stanford University, including diagnosis and treatment of spontaneously occurring health conditions. Animals in declining health despite medical care were euthanized for humane reasons as determined by a veterinarian. Before euthanasia, it happened that all four lemurs were living in summer-like long days (14:10 h) for at least 3 months (range 3–6 months), and all showed standard activity patterns without signs of torpor. Given these individuals were housed at constant temperature conditions and fed a non-calorie-restrictive diet, spontaneous torpor was not observed in any of the analysed lemurs throughout their time in the Stanford colony.

### Tissue collection and processing

Animals in declining health who did not respond to standard therapy were euthanized by pentobarbital overdose under isoflurane anaesthesia as previously described^15^. Before euthanasia, a veterinary examination was performed, and animal body weight and electrocardiogram data were obtained (KardiaMobile 6L, AliveCor). Blood was immediately collected by cardiocentesis for serum chemistry, complete blood count, biobanking and scRNA-seq. In three animals (L2–L4), transcardial perfusion of the lungs with PBS was done to reduce circulating cells. Organs and tissues were sequentially removed and divided by a veterinary pathologist. One sample of each tissue was immediately placed in formalin fixative for histopathology^15^, and a second was embedded in optimal cutting temperature compound and then flash-frozen on dry ice and stored at −80 °C for biobanking. A third sample was placed directly in cold (4 °C) PBS pH 7.4 and immediately distributed to the tissue expert for cell dissociation and preparation for scRNA-seq as detailed below. Additional diagnostics, such as microbiological cultures, were performed where clinically indicated. The entire necropsy was completed within 1–2 h, with ischaemia-sensitive tissues prioritized as described in the Supplementary Methods.

### Histological and pathological analysis

Tissues were immersion-fixed in 10% neutral-buffered formalin for 72 h. Formalin-fixed tissues were processed routinely, embedded in paraffin, sectioned at 5 µm and stained with haematoxylin and eosin (H&E). The following tissues were analysed: heart, aorta, lungs, trachea, thyroid gland, parathyroid gland, kidneys, urinary bladder, male reproductive tract (testicle, epididymis, seminal vesicle, prostate and penile urethra), female reproductive tract (uterus, cervix, vagina and ovaries), salivary glands, tongue, epiglottis, oesophagus, stomach, small and large intestine, liver (with gallbladder), adrenal gland, spleen, lymph nodes, white adipose, brown adipose, bone, spinal cord, eyes and bone marrow. Selected tissues were stained with Von Kossa (for mineralization), Masson’s trichrome (for collagen), Congo Red (for amyloid) and Gram stain (for bacteria) as part of the pathological analysis. H&E-stained slides were scanned with a Leica Aperio AT2 high-volume digital whole-slide scanner (×40 objective), uploaded into Napari image viewer^44^, software adapted by CZB and posted on the Tabula Microcebus portal.

### Preparation of single-cell suspensions and FACS for scRNA-seq

Fresh tissue samples obtained as described above were placed on ice, delivered to tissue experts and immediately dissociated and processed into single-cell suspensions, except for samples from L3, which were kept cool overnight after necropsy and processed the next morning (Supplementary Methods). For each solid tissue, this process involved a standard combination of enzymatic digestion and mechanical disruption methods that were optimized for the specific tissue, many of which were adapted from procedures used for the corresponding mouse tissue^12,13^. For blood, immune cells were isolated using a high-density Ficoll gradient (Histoplaque-1119, Sigma-Aldrich) to include peripheral blood mononuclear cells and polymorphonuclear leukocytes^14^.

The specific protocols for each of the 27 tissues are detailed in the Supplementary Methods. The cell number and concentration for each single-cell suspension were determined by manual counting using a haemocytometer and then adjusted with 2% FBS in PBS to a target concentration of about 10^6^ cells per ml. Samples were then used for droplet-based 10x library preparation and/or flow sorted for single live cells (Sytox blue negative; ThermoFisher, S34857) for plate-based SS2 library preparation (Supplementary Fig. 5). To enrich for cardiomyocytes, the standard procedure for cardiac cell isolation was supplemented by hand-picking cardiomyocytes (Supplementary Methods). Residual cell suspensions were diluted 1:1 with serum-free Bambanker cell freezing medium (GC Lymphotec, BB01) and cryopreserved at −80 °C.

### scRNA-seq library preparation, quality control and sequencing

For 10x, single cells were profiled using the 10x Genomics scRNA-seq pipeline (Chromium Single Cell 3′ Library and Gel Bead v.2 Chemistry kit) and sequenced on a NovaSeq 6000 System as previously described^12–14^ and detailed in the Supplementary Methods. For SS2, single cells were sorted into 384-well or 96-well lysis plates, reverse transcribed to cDNA and amplified, as previously described^12,13^. cDNA libraries were prepared using a Nextera XT Library Sample Preparation kit (Illumina, FC-131-1096) or (for L4) an in-house protocol detailed in the Supplementary Methods; no significant differences between protocols were observed in library read depth or quality. Pooling of individual libraries and subsequent quality control and DNA sequencing were done as previously described^12–14^ with minor modifications (Supplementary Methods). Both 10x and SS2 libraries were sequenced to achieve saturation on an Illumina NovaSeq 6000 system (10x, 26 bp and 90 bp paired-end reads; SS2, 2 × 100 bp paired-end reads).

### Genome alignment of scRNA-seq reads and gene counts

The *M.* *murinus* genome assembly (Mmur 3.0, accession: GCF_000165445.2; annotation: NCBI Refseq annotation release 101) with NCBI annotation release 101 (date acquired, 21 September 2018) was used for downstream alignment and data analyses. A total of 31,509 genes were detected, including annotated genes and unannotated loci but excluding mitochondrial and Y chromosome genes (unannotated at our acquisition date).

For 10x samples, downstream data were processed using standard methods with Cell Ranger (v.2.2, 10x Genomics). Raw base call files directly generated by the NovaSeq instrument were demultiplexed and converted to fastq files and then aligned to the 10x genome index, with barcode and UMI counting performed to generate a gene counts table. Alignment files were outputted in standard BAM format.

For SS2 samples, demultiplexed fastq files were mapped to the genome using STAR aligner (v.2.6.1a). In brief, the genome FASTA file was augmented with ERCC sequences to create a STAR genome index with 99 bp overhangs (optimized for Illumina 2 × 100 bp paired-end reads). Two-pass mapping was executed, in which the first pass identified splice junctions that were added to the gene reference to improve second pass mapping, with specific STAR options and parameters detailed in the Supplementary Methods.

### Contamination filtering of 10x data

We performed stringent contamination filtering to resolve cross-sample contamination in an Illumina sequencing run caused by cell barcode hopping among multiplexed 10x samples^45,46^. Such cross-sample contamination can occur when low levels of ambient mRNA containing the 10x cell barcode in one sample gets added onto the transcript of other samples during Illumina sequencing amplification, which results in the incorrect assignment of a cell barcode to other samples. Hence, in subsequent analyses, a cell from one tissue could falsely appear as multiple cells from different tissues (or samples). To exclude such artefacts, for each sequencing run, we identified all cell barcodes that were assigned to multiple samples. Then for each such barcode identified, we compared the number of UMIs in each sample. If there was one dominant sample index (that is, the number of UMIs of the dominant sample was ten times or more greater than that of the second most abundant sample), then the cell with the dominant sample index was kept (but labelled in its metadata as ‘potentially contaminated’), whereas all other instances of that ‘cell’ were removed. If there was no dominant sample index, then all instances of the ‘cell’ with that barcode were removed from the dataset. Contamination was not an issue for SS2 samples because they were sequenced using dual unique indices for each cell.

### Cell clustering, annotation and cluster markers from scRNA-seq profiles

#### Cell clustering and annotation of each tissue processed by 10x

Transcriptomic profiles of cells from each tissue and from each individual lemur were clustered separately using Seurat software (v.2.3.0) for R studio (v.3.6.1). We included in this step all cells with >100 genes or >1,000 UMIs detected, a minimal threshold that was used to ensure the inclusion of all cell types, including ones in which the cells (or RNA) were unstable (see below for more stringent criteria used for final cell quality control). For each cell, expression of a gene *g* was normalized in 10x data as follows: ln(UMI_*g*_/UMI_total_ × 1 × 10^4^ + 1), abbreviated as ln(UP10K + 1); in SS2: ln(reads_*g*_/reads_total_ × 1 × 10^4^ + 1), abbreviated as ln(CP10K + 1). Next, data scaling, dimensionality reduction (PCA), clustering and visualization (t-SNE and UMAP) were performed following the standard Seurat pipeline as previously described^14^, with parameters including the numbers of principal components (PCs), perplexity and resolution manually adjusted for each iteration of cell clustering. Resultant cell clusters were manually assigned to a compartment (endothelial, epithelial, stromal, lymphoid, myeloid, megakaryocyte–erythroid, haematopoietic (for precursors), neural and germ) on the basis of the expression of the mouse lemur orthologues of canonical marker genes for each compartment in human and mouse (Supplementary Table 1). Clusters that expressed markers from more than one compartment were annotated as ‘doublets’. Cells in each assigned compartment were then separately subclustered, repeating the data processing steps above. To annotate (determine) the cell type of each cluster in a compartment, a list of canonical human and mouse gene markers for each cell type in each tissue was curated from the literature (Supplementary Table 1), including genes previously validated by in situ hybridization and/or immunohistochemistry as well as DEGs selected from recent scRNA-seq studies. The orthologous mouse lemur genes were identified and their expression visualized on the t-SNE plots. On the basis of the enriched expression of marker genes, each cluster of cells in a compartment was manually assigned a cell-type identity. Clusters that contained more than one cell type were further subclustered to better resolve the cell types. Cell types represented by only a small number of cells that did not form a separate cluster were manually curated, aided by the cellxgene gene expression visualization tool^47^ as detailed below.

Each cluster was assigned both a ‘cell ontology’ cell-type designation (name) using the standardized and structured nomenclature^19^ and a ‘free annotation’ that resolved biologically significant clusters not contained in the current cell ontology. Free annotations were assigned as follows. In cases when a smaller cluster stemmed off a larger (main) cluster in the t-SNE-embedded space, the smaller cluster was distinguished with one or more DEGs added to the cell-type name (for example, B cell (SOX5^+^) clustered near the main population of B cells in the pancreas). DEGs driving the subtype clustering were ascertained by Wilcoxon rank-sum tests. In cases when two approximately equal-sized clusters separated on the t-SNE plot, a marker gene was added to the cell-type name for both clusters. Clusters with a small number of cells that contained more than one cell type but could not be partitioned into separate clusters by subclustering with the Louvain algorithm or manually with cellxgene (see the section ‘Integration of datasets across individuals’) were labelled as a ‘mixed’ cell type (for example, the cluster labelled ‘endothelial cell’ in the uterus contains a mixture of artery, vein and capillary cells). Clusters with cells that expressed markers for more than one cell type and it was biologically plausible that they were not a technical artefact (for example, a doublet of two distinct cell types) were labelled as a ‘hybrid’ cell type (for example, the cluster labelled as ‘monocyte–macrophage’ in the trachea contains cells that expressed markers of both cell types and could not be further distinguished based on current molecular definitions of these cell types). After examining the human and mouse markers for all known cell types in a tissue, clusters that could not be assigned a cell type were labelled ‘unknown’, with the tissue, compartment and one or more DEGs added to the cell type name (for example, ‘unknown bone stromal G1 (NGFR^+^TNNT2^+^)’ are bone stromal cells that do not correspond to any extant stromal cell type reported for humans or mice). To detect the DEGs of an unknown cell type, we compared the unknown cell type to all other cells of the same compartment and tissue (Extended Data Fig. 4 and Supplementary Fig. 3). Clusters containing a majority of cells that expressed cell proliferation markers (for example, *TOP2A*, *MKI67* and *STMN1*) were appended the abbreviation ‘PF’. Clusters that separated from a main cluster but did not express any distinguishing markers (other than tRNAs, rRNAs and/or immediate-early genes) and differed only in parameters of technical quality (that is, fewer genes and counts detected per cell) were considered low quality and ‘LQ’ was appended to the cell-type name.

After annotations were assigned, the cut-off for the minimum number of genes per cell was increased from 100 to 500, and only the qualifying cells were further analysed. For most tissues, this more stringent cut-off value only resulted in removal of some erythrocytes and neutrophils. The only exception were cardiac cardiomyocytes, most of which expressed fewer than 500 genes per cell; therefore, separate filtering criteria were applied (Supplementary Methods).

#### Annotation of each tissue processed by SS2

Cells processed using the SS2 protocol with <500 genes or <5,000 reads were excluded from further analysis, and gene expression levels in the remaining cells were scaled and log transformed as described above for the 10x datasets. Cells from a particular tissue and individual were integrated with the 10x dataset of the same tissue and individual into the same UMAP-embedded space using the FIRM algorithm (detailed below). Cells from SS2 were automatically annotated with the same label as the nearest neighbouring 10x cell. Annotations were manually verified as described in the section ‘Integration of datasets across individuals’, aided by cellxgene gene expression visualization. SS2 datasets for which there were no corresponding 10x dataset from the same individual or tissue were manually annotated using the method described in the section ‘Cell clustering and annotation of each tissue processed by 10x’ for 10x datasets.

#### Integration of datasets across individuals

For each tissue, the combined 10x and SS2 datasets from each individual were further integrated into the same UMAP-embedded space using the FIRM algorithm^18^. This step resulted in 27 separate tissue UMAPs, each containing data from up to 4 individuals. To ensure consistency of cell-type labelling across all individuals, annotations were verified or manually adjusted using cellxgene, an interactive tool to visualize and annotate scRNA-seq data (https://chanzuckerberg.github.io/cellxgene/)^47^.

#### Integration of datasets across tissues

All 27 tissue-level objects were integrated into a single UMAP-embedded space using the FIRM algorithm. As described above, annotations were verified or manually adjusted in cellxgene to ensure consistency of cell designations across all tissues. In most instances, cells from the same cell type clustered together, irrespective of the tissue of origin, and the same designation was used across all tissues. Occasionally, similar cells types (for example, fibroblasts and macrophages) clustered separately by tissue of origin, which made it challenging to distinguish whether the separation was due to a tissue-level batch effect or because of true biological differences. In these cases, the original tissue-level annotation label was kept for each cluster. In total, 256 cell designations were assigned across the integrated atlas, which, when distinguished by organ of origin (for example, lung versus bladder artery cells), resulted in a total of 768 molecular cell types.

#### Detection of DEGs for each cell type

We calculated the top 300 DEGs (adjusted *P* < 0.05) for each cell type in the 10x dataset (represented by at least 5 individual cells after removing doublets, low-quality cells and mixed cell types) using two-tailed Wilcoxon rank-sum tests with Benjamini–Hochberg false discovery rate correction (Supplementary Table 3). We compared each cell type to the following: (1) all other cell types from the same tissue (for example, lung capillary cells compared with all other lung cells; ‘tissue-wide’ comparison); (2) all other cell types from the same compartment of that tissue (lung capillary cells compared with all other lung endothelial cells; ‘tissue-compartment-wide’ comparison); (3) all other cell types from the atlas (lung capillary cells with all other cells in the atlas; ‘atlas-wide’ comparison); and (4) all other cell types from the same compartment across the atlas (lung capillary cells with all other endothelial cells in the atlas; ‘atlas-compartment-wide’ comparison).

### FIRM integration

FIRM is a newly developed algorithm that integrates multiple scRNA-seq datasets^18^ (for example, from different sequencing platforms, tissue types and experimental batches). In brief, FIRM optimizes dataset integration by harmonizing differences in cell-type composition and computing the dataset-specific scale factors for gene-level normalization. Different datasets generally have varied cell-type compositions, which results in dataset disparity when scaling the gene expression levels to the unit variance for each dataset. Different from classical scaling procedures, FIRM computes the scale factors based on subsets of cells that have matched cell-type compositions between datasets. To construct these subsets, FIRM detects paired clusters between datasets based on similar overall gene expression levels and then samples the cells so that paired cell types have the same proportional representation in each dataset. The parameters used for integration are given in the Supplementary Methods. The integrated datasets generated using FIRM showed accurate mixing of shared cell-type identities and preserved the structure of the original datasets, as confirmed by expert manual inspection during cell annotation.

### Trajectory analysis

We used two independent methods to characterize spatial and developmental pseudotime cell trajectories: a custom in-house program in Matlab and Slingshot^48^. For the mouse lemur kidney nephron spatial trajectory, all kidney epithelial cells were included in the analysis, with the exception of podocytes, macula densa cells, intercalated cells and urothelial cells which clustered separately. For the vasa recta endothelium spatial trajectory, all four vasa recta cell types were used. For the spermatogenesis pseudotime trajectory, all seven sperm and sperm progenitor cell types were used. For the myeloid cell developmental pseudotime trajectory, haematopoietic precursor cells and all myeloid cell types except DCs (which did not form part of the continuum) were used. Analysis was performed independently for each trajectory using values from the 10x scRNA-seq profiles of the indicated cells (low-quality cells and technical doublets were excluded) that had been pre-processed (scaled: ln(UP10K + 1), normalized) as described above.

PCA with highly variable genes (dispersion > 0.5) was done with the PCA function of Matlab, and the high-quality PCs (not driven by extreme outlier data points or immediate-early genes) were selected from the top 20 PCs and used to generate a 2D UMAP using cell–cell Euclidean distances as input (https://www.mathworks.com/matlabcentral/fileexchange/71902). The trajectory of the cell continuum was detected as the probability density ridge of the data points in the UMAP, using automated image processing (Matlab Image Processing Toolbox). Any interruptions in the detected density ridge line were manually connected along the direction of the ridge line and guided by previous knowledge of the biological process. The direction of the trajectory was assigned on the basis of expression of marker genes. Individual cells were then aligned to the trajectory by the shortest connecting point to the trajectory; if the trajectory branched (for example, in myeloid cell development), cells were assigned to the closest branch. Individual cells that were too distant from the trajectory (adaptive thresholding along the trajectory) were deemed outliers and removed from further analysis.

To detect genes for which expression followed the trajectory, we calculated Spearman correlation coefficients and corresponding *P* values (Bonferroni corrected) between the expression level of each gene and 20 preassigned unimodal patterns that smoothly changed along the trajectory (with their single peaks uniformly distributed from the beginning of the trajectory to its end point). Expression patterns of the top ranking (top 1,000 with *P* < 0.01) and highly variable (dispersion > 0.5) genes were smoothed with a moving average filter and clustered by *k*-means clustering to detect the major trajectory-dependent expression patterns. The trajectory DEGs were then ranked by the associated cluster (ranked by trajectory location of peak expression), in the cluster by *P* value from the smallest to the largest, and with the same *P* value by mean expression level from the highest to the lowest.

For the myeloid cell analysis, four trajectories were independently detected: (1) from haematopoietic precursors to granulocyte–monocyte progenitors; (2) from granulocyte–monocyte progenitors to proliferating and then maturing neutrophils; (3) from granulocyte–monocyte progenitors to proliferating and then maturing monocytes and macrophages; and (4) from megakaryocyte and erythroid progenitors to proliferating and maturing erythroid lineage cells. On the UMAP, trajectory 1 branched into trajectories 2 and 3, so two longer trajectories were generated (1 + 2 and 1 + 3). Differential gene expression analysis was then independently performed for each of the constituent trajectories (1 + 2, neutrophil lineage; 1 + 3, monocyte–macrophage lineage; 4, erythrocyte lineage).

As an alternative method, we applied the Slingshot method^48^, which first computes the global lineage structure by constructing a cluster-based minimum spanning tree followed by pseudotime inference using simultaneous principal curves to fit smooth branching curves to these lineages. We used the annotated clusters and UMAP coordinates to first obtain a global lineage structure with getLineages, then constructed smooth curves, ordered cells along the trajectory and generated pseudotime values using getCurves. For each tissue, the longest trajectory that incorporated the most clusters was used. For the immune cell trajectories, the neutrophil cluster was subdivided into higher resolution clusters that were then combined to facilitate building of the minimum spanning tree. For each trajectory, coordinates were normalized by the maximal value for comparison with the other method.

### Comparison of expression profiles among mouse lemur cell types

#### UMAP of cell types

To visualize similarities among the mouse lemur cell-type expression profiles, we embedded the high-dimensional 10x scRNA-seq expression data (around 30,000 genes) into a 2D UMAP. Cell types that were low quality (labelled with LQ in free annotation) or represented by fewer than 4 individual cells were excluded, which resulted in a comparison of 681 molecular cell types. Cell types were treated as pseudo bulk, with expression levels calculated for each gene by averaging the expression level of all cells within the same cell type and then taking the natural log transform (ln(avg count per 10K UMIs + 1)). Expression levels were further normalized by the maximal value of each gene across all cell types so that all ranged from 0 to 1. The cell-type gene expression matrix was then projected onto a 2D space with cosine distances between pairs of cell types used in the UMAP function (https://www.mathworks.com/matlabcentral/fileexchange/71902). Wilcoxon rank-sum tests were used to identify DEGs that distinguished related molecular cell types identified in the cell-type UMAP (for example, mature and progenitor sperm cells plus progenitor and proliferating immune cells versus proliferating cells of other compartments) as described in the Supplementary Methods.

#### Heatmap of cell-type pairwise correlation scores

To compare the overall gene expression profiles of cell types, Pearson’s correlation scores were calculated for every pair of cell types. Given data were obtained from different sequencing platforms (10x and SS2), we used the FIRM-integrated dataset as described above, which contains FIRM-generated PC coefficients for each cell. Cell types were treated as pseudo-bulk, and the cell-type average PC coefficients were calculated and used to determine the correlation coefficients. The cell-type pairwise correlation scores were plotted as a heatmap matrix (Extended Data Fig. 5d). Interactive forms of the heatmap matrix are available online at the Tabula Microcebus portal.

### Evolutionary comparison of mouse lemur, human, macaque and mouse transcriptional profiles

#### Compiling comparable datasets

For the cross-species comparisons, we used published human, mouse and macaque scRNA-seq (and not single nucleus) datasets that were obtained using methods similar to those described above for the mouse lemur. These datasets included lung and muscle cells (from all compartments), epithelial cells of the liver, immune cells of the bone marrow and spleen, and germ cells of the testes (Supplementary Table 4). We manually re-annotated cells where necessary for consistency with the lemur annotations (see below). All lemur data were from the 10x data of this study with additional muscle data from L5 (ref. ^22^). Human data were from the 10x data of the Tabula Sapiens^29^, except for the lung, for which we used the 10x data from the Human Lung Cell Atlas^14^ and the testes, for which we used previously published drop-seq data^35^. Mouse data were from the 10x data of the Tabula Muris Senis^13^, except for the testes, for which we used previously published 10x data^36^. Given the limited data availability (either lack of the tissue or relevant cell types), we analysed only the lung and testes for the macaque data. Crab-eating macaque lung data were from previously published 10x data^37^ and rhesus macaque testis data were from previously published drop-seq data^35^. All datasets profiled adult animals; we excluded mouse postnatal developmental data from the analyses for consistency.

#### Orthology mapping across species

For orthology mapping, we merged the orthology databases from both NCBI and Ensembl (Supplementary Tables 5). We began by compiling all mouse lemur genes annotated in the NCBI (mouse lemur taxonomy ID: 30608), then merged the corresponding human and mouse orthologues from NCBI (gene_info.gz and gene_orthologs.gz from https://ftp.ncbi.nlm.nih.gov/gene/DATA/, February 2020). We next added Ensembl gene identifier (ID) numbers, gene names and human and mouse orthologue assignments from Ensembl Biomart (Ensembl Genes v.99, February 2020) using the Ensembl gene ID (variable ‘Gene_stable_ID’) for each NCBI gene ID (variable ‘NCBI_gene_ID’) in Ensembl Biomart. Mouse lemur genes that did not have an assigned human or mouse orthologue in either Ensembl or NCBI were removed, as were mouse lemur genes that had more than one human or mouse orthologue assigned, or that shared the same human or mouse orthologue with another mouse lemur gene. Note that unlike NCBI, Ensembl specifies the type of orthologue assignment (for example, ‘ortholog_one2one’ or ‘ortholog_one2many’); however, we did not use the Ensembl specification to filter one-to-one-to-one orthologues because, occasionally, a mouse lemur gene name was assigned by homology to multiple currently unnamed loci in Ensembl and because of this imperfect genome annotation, was labelled as sharing an ‘ortholog_one2many’ with human or mouse instead of ‘ortholog_one2one’. Finally, we appended the one-to-one orthologues between human and rhesus macaque and between human and crab-eating macaque, as assigned by Ensembl. A total of around 15,000 one-to-one gene orthologues were therefore uncovered across human, lemur and mouse genomes, around 14,000 across human, lemur, mouse and rhesus macaque genomes, and around 13,000 across human, lemur, mouse and crab-eating macaque genomes (Supplementary Table 5). Sequence identity was based on those reported in the Ensembl homology database.

#### Integrating cross-species datasets and unifying cell-type annotations

For the cross-species comparisons, we used the one-to-one gene orthologues that existed in all relevant datasets. Orthology mapping for the datasets was based on the NCBI or Ensembl gene ID if the original datasets provided the respective gene ID, and on the gene symbol if the gene ID was not provided. The choice of NCBI versus Ensembl depended on which version of the genome annotations the original dataset was aligned to. Some of the one-to-one orthologues were missing from one or more of the datasets; therefore, these were removed from the cross-species comparison. Together, we identified around 13,000 genes for the comparisons across human, lemur, and mouse genomes, and around 12,000 genes for the comparisons that also include either of the macaque species.

To unify cell-type annotations, human, mouse and macaque datasets were first re-annotated separately for each tissue and species using the same pipeline and marker genes as for the lemur data. For the male germ cells that formed a molecular gradient, we simplified the annotations into three discrete stages (spermatogonia, spermatocytes and spermatids) based on their original annotations and applied trajectory analysis (see below). Next, to ensure consistency of cell annotations across species, we applied Portal^38^ to integrate data from different species. Through adversarial learning of neural networks, Portal projects data into a space that minimizes species differences, from which an integrated UMAP is generated to visualize cell clustering from different species. Portal integration was performed separately for each tissue, except for bone marrow and spleen, which were jointly integrated. We manually inspected each integration UMAP and ensured that cells of the same designation showed reasonable cross-species co-clustering and separation from other cell types. We also made minor modifications to the cell annotations during this process to unify designations across species. For example, proliferating cells might co-cluster with the main non-proliferating population of the same cell type in the original dataset if the number of proliferating cells were too few (and they thus could not be distinguished by separate annotations), but they often formed a separate cluster with the proliferating populations of the other species in the integrated UMAP. In such a scenario, we re-annotated these cells as a proliferating subtype. We also merged cell types that had unclear cross-species correspondence and were almost indistinguishable in the species-integrated UMAP (for example, proliferating T, NK and NKT cells were grouped together and designated NK/T cells (PF)).

As additional validations of annotation consistency across species, we applied SAMap^39,49^, a self-assembling manifold algorithm and graph-based data integration method, to the lung and muscle datasets in order to identify orthologous (reciprocally connected) cell types on the basis of shared expression profiles across species. Cross-species cell-type similarity (visualized by the edge width in Extended Data Fig. 6d,e) is defined as the average number of cross-species neighbours of each cell relative to the maximum possible number of neighbours in the combined manifold. The default SAMap parameters were used in the analysis, and similarity scores less than 0.1 were removed.

#### Identifying species-unified trajectories

Trajectories were calculated for spermatogenesis across human, macaque, lemur and mouse datasets, as well as for three myeloid lineages (neutrophil, monocyte–macrophage and erythroid) of haematopoiesis across human, lemur and mouse datasets (macaque data not available). Trajectory detection and cell alignment was performed using the same custom in-house program as described above (in trajectory analysis), with the species-integrated UMAPs as input.

#### Calculating cross-species similarity scores for each cell type

Cell types with more than 15 cells in each of the species were used for the cross-species comparisons. This resulted in a total of 63 orthologous cell types for the comparisons across human, lemur and mouse data (63 × 3 = 189 total cell-type entries across all species), and 18 cell types for the comparisons of the lung cell types across human, macaque, lemur and mouse data (18 × 4 = 72 cell-type entries). Cell-type mean gene expression was calculated for each gene. Single-cell expression levels in the species-integrated dataset were normalized and log-transformed the same way as described above for the lemur-only dataset. That is, ln(UMI_*g*_/UMI_total_ × 1 × 10^4^ + 1). Note, however, that because there were fewer genes in the cross-species dataset (only one-to-one orthologues), the absolute expression levels were higher than that in the lemur-only dataset.

We used correlation coefficients as a proxy for cross-species similarity. To score similarity for individual cell types, we calculated Spearman rank-based correlation coefficients of cell-type mean expression levels between human and lemur, *r*_c_HL and between human and mouse, *r*_c_HM. The cell-type mean expression levels were thresholded at ≥0.4 to mitigate the effect of background noise. Cross-species similarity was similarly calculated for cells at different stages and lineages of spermatogenesis and haematopoiesis by applying a moving window along the respective trajectories.

#### Calculating cross-species similarity scores for each gene

Cross-species similarity was calculated separately for individual genes using the tissue and three species (human, lemur and mouse) integrated dataset across the 63 orthologous cell types. We first quantified the mean expression (*E*_max_) in the maximally expressed cell type in each species. Next, we filtered genes that were not expressed or expressed at low levels across the analysed cell types, requiring *E*_max_ > 0.5 in all three species, or *E*_max_ > 0.1 in all three species with *E*_max_ > 1.5 in at least one species. This resulted in a total of 7,787 genes for follow-up analysis. Mean cell-type expression levels across the 63 cell types were then normalized by *E*_max_ in each species and Pearson’s correlation coefficients between human and lemur (*r*_*g*_HL), human and mouse (*r*_*g*_HM) and lemur and mouse (*r*_*g*_LM) were calculated. We then calculated Δ*r*_g_ = *r*_g_HL – *r*_*g*_HM for each gene and tested the *P* value for Δ*r*_*g*_ being significantly higher (right-tailed) or lower (left-tailed) than 0 (see below). To identify genes with human–lemur-conserved but mouse-divergent expression patterns (that is, HL genes), we applied a threshold of Δ*r*_*g*_ > 0.4 and a right-tailed *P* value < 0.05. We also identified human–mouse-conserved and lemur-divergent (HM) genes and lemur–mouse-conserved and human-divergent (LM) genes using the same threshold levels. A similar analysis was also performed to detect genes that showed species-conserved or species-diverged expression patterns along the spermatogenesis trajectory and the neutrophil lineage of the haematopoiesis trajectory. We also identified genes that are highly conserved (*r*_*g*_ > 0.8), lowly conserved (*r*_*g*_ < 0.3) or moderately conserved (*r*_*g*_ > 0.3 and *r*_*g*_ < 0.8) in all three species. A full list of analysed genes and their statistics is provided in Supplementary Table 7. Expression patterns of example genes is visualized in Supplementary Fig. 4. Gene set enrichment analysis was performed using gProfiler^50^ for the highly conserved genes and HL genes, with all the analysed genes provided as a custom background gene set and otherwise default parameters.

To test whether one correlation coefficient (*r*) was significantly higher or lower than the other, we estimated the significance of their difference (Δ*r*) being larger or smaller than 0 through Fisher’s *Z*-transformation. In essence, correlation coefficients, which were bounded and not normally distributed, were Fisher’s *Z*-transformed to the unbounded and approximately normally distributed space using the inverse hyperbolic tangent function, and their difference and respective *P* value were calculated using standard one-tailed *t*-tests in this transformed space. For display purposes, the mean and 95% confidence intervals were then inverse transformed and displayed in Fig. 5a and Extended Data Figs. 9a and 10a. Note that this inverse transformed Δ*r*, which is bounded between −1 and 1, does not necessarily equal the initial Δ*r*, which is between −2 and 2.

#### Identification of genes with primate-selective expression for each cell type

Using the cross-species dataset across human, lemur and mouse, we performed two separate Wilcoxon rank-sum tests for each gene and for each of the 63 orthologous cell types. The first was a two-tailed test comparing expression in lemur versus human, lemur versus mouse and human versus mouse. The second was a one-tailed test comparing expression in a cell type versus the rest of the cell types in the dataset (independently of the species). We calculated the fold change in mean expression for the above comparisons. Next, for each cell type, we searched for three categories of genes. First, genes with significantly primate-enriched expression, which requires that (1) cell-type mean expression of the gene is above 0.5 in both humans and lemurs and (2) 5-fold greater expression and *P* < 1 × 10^–5^ in both species compared with the orthologous mouse cell type. Second, genes with significantly primate-depleted expression, which requires that (1) cell-type mean expression of the gene is above 0.5 in mouse and (2) 5-fold lower expression and *P* < 1 × 10^–5^ in human and lemur compared with the orthologous mouse cell type. Third, genes that are significantly enriched in a cell type, which requires that (1) cell-type mean expression of the gene is above 0.5 in all three species and (2) 5-fold greater expression and *P* < 1 × 10^–5^ when comparing this cell type versus other cell types. The full list of the identified genes is provided in Supplementary Table 6.

### Reporting summary

Further information on research design is available in the Nature Portfolio Reporting Summary linked to this article.

## Online content

Any methods, additional references, Nature Portfolio reporting summaries, source data, extended data, supplementary information, acknowledgements, peer review information; details of author contributions and competing interests; and statements of data and code availability are available at 10.1038/s41586-025-09113-9.

## Supplementary information

Supplementary Information Supplementary Notes, Supplementary Figures, Supplementary Table legends, Supplementary Methods and Supplementary References.

Reporting Summary

Supplementary Tables Supplementary Tables 1–8. See Supplementary Information for Supplementary Table 9.

Supplementary Data 1 Source data for Supplementary Fig. 3.

Supplementary Data 2 Source data for Supplementary Fig. 4.

## Source data

Source Data Fig. 2

Source Data Fig. 3

Source Data Fig. 4

Source Data Fig. 5

Source Data Extended Data Fig. 2

Source Data Extended Data Fig. 3

Source Data Extended Data Fig. 4

Source Data Extended Data Fig. 5

Source Data Extended Data Fig. 7

Source Data Extended Data Fig. 8

Source Data Extended Data Fig. 9

Source Data Extended Data Fig. 10

## Data Availability

Tabula Microcebus mouse lemur scRNA-seq gene expression counts and UMI tables, and cellular metadata used in this study are available from Figshare (https://figshare.com/projects/Tabula_Microcebus/112227)^51^ and can be explored interactively using the UCSC Cell Browser on the Tabula Microcebus portal (https://tabula-microcebus.ds.czbiohub.org/). A histological atlas of all tissues analysed is also available on the portal. Raw sequencing data (fastq files) are available from Globus (https://app.globus.org/file-manager?origin_id=c9fc0a15-54a0-4182-8d64-fd8afc12f1fc&origin_path=%2F). For sequence alignment, the *M.* *murinus* genome assembly (Mmur 3.0, NCBI accession: GCF_000165445.2) and the gene annotation file (NCBI Refseq Annotation Release 101) were obtained from NCBI’s FTP sites (https://www.ncbi.nlm.nih.gov/datasets/genome/GCF_000165445.2/; https://ftp.ncbi.nlm.nih.gov/genomes/all/annotation_releases/30608/101/). For cross-species analysis, human 10x data were from the Tabula Sapiens^29^ for the liver, spleen and bone marrow (https://figshare.com/projects/Tabula_Sapiens/100973) and the Human Lung Cell Atlas^14^ for the lung (https://www.synapse.org/#!Synapse:syn21041850/wiki/600865). Human and rhesus macaque testis drop-seq data were from a previous study^35^ (https://www.ncbi.nlm.nih.gov/geo/query/acc.cgi?acc=GSE142585). Crab-eating macaque lung 10x data were from a previous study^37^ (https://zenodo.org/record/5881495#.ZERMCnbMKUk). Mouse data were all from 10x data of the Tabula Muris Senis^13^ (https://figshare.com/articles/dataset/Processed_files_to_use_with_scanpy_/8273102/2), except for the testis, which was based on 10x data from a previous study^36^ (https://www.ebi.ac.uk/biostudies/arrayexpress/studies/E-MTAB-6946). For orthologous genes compilation, mouse lemur genes and corresponding human and mouse orthologues were obtained from the NCBI (gene_info.gz and gene_orthologs.gz from https://ftp.ncbi.nlm.nih.gov/gene/DATA/) and Ensembl Biomart (Ensembl Genes v.99). The list of human genes with associated genetic disorders was obtained from Online Mendelian Inheritance in Man (genemap2.txt from https://www.omim.org/downloads). Source data are provided with this paper.

## Code availability

Custom computer codes are available on Globus (https://app.globus.org/file-manager?origin_id=c9fc0a15-54a0-4182-8d64-fd8afc12f1fc&origin_path=%2F). Additional software and packages used are described below. Raw sequencing data were processed using Cell Ranger (v.2.2, 10x Genomics) for 10x data and STAR aligner (v.2.6.1a), skewer (v.0.2.2), RSEM (v.1.3.1) and HTSEQ (v.2.0) for SS2 data. Downstream analyses were performed using R (v.4.3.0), Python (v.3.6 and v.3.9) and Matlab (v.2020b). Seurat (R package, v.2.3.0), Scanpy (v.1.8) and cellxgene (v.1.0.1) were used for cell clustering and annotation. Cell gradients were generated using Slingshot (v.2.14.0) and a custom program developed in Matlab (Trajectory analysis: https://github.com/Shixuan1/scRNAseq_trajectory_analysis) using Matlab built-in functions (for example, pca), the Image Processing Toolbox (Matlab v.2020b) and a Matlab umap package (https://www.mathworks.com/matlabcentral/fileexchange/71902). scRNA-seq data integration used custom programs developed by co-authors, including FIRM (https://github.com/mingjingsi/FIRM) and Portal (https://github.com/YangLabHKUST/Portal). Gene set enrichment analysis used gprofiler2 in R (v.0.2.1). For data visualization, dot plots, sina plots, violin plots, line plots, bar plots, box plots, heatmaps, pie charts, error bars and contour figures were generated using the following packages and functions: Python packages pandas (v.1.1.5), numpy (v.1.19.3), anndata (v.0.7.4), scanpy (v.1.6.0), matplotlib (v.3.3.2), igraph (v.0.7.1), seaborn (v.0.9.0) and louvain (v.0.6.1); R packages ggplot2 (v.3.4.4), gplots (v.3.1.3), readr (v.2.1.4), dplyr (v.1.1.2), reshape2 (v.1.4.4), patchwork (v.1.1.3), RColorBrewer (v.1.1.3), ggrepel (v.0.9.4), aplot (v.0.1.10), ggdendro (v.0.1.23), Matrix (v.1.6.4), here (v.1.0.1), pheatmap (v.1.0.12), tidyr (v.1.3.0) and cowplot (v1.1.1); and the Matlab built-in functions plot, scatter, violinplot, imagesc, contour, bar, box, errorbar and pie.
