# Supplementary material for: A molecular cell atlas of mouse lemur, an emerging model primate
Source: Nature. 2025 Jul 30;644(8075):173–84. doi: 10.1038/s41586-025-09113-9 (PMC12328211; doi:10.1038/s41586-025-09113-9)
Supplement: Supplementary file 1 — Supplementary Notes, Supplementary Figures, Supplementary Table legends, Supplementary Methods and Supplementary References. [file 41586_2025_9113_MOESM1_ESM.pdf]

nature portfolio

https://doi.org/10.1038/s41586-025-09113-9

Supplementary information

A molecular cell atlas of mouse lemur, an emerging model primate

In the format provided by the authors and unedited

A molecular cell atlas of mouse lemur, an emerging model primate

The Tabula Microcebus Consortium*, Camille Ezran[1, 2‡], Shixuan Liu[1, 2, 3‡], Stephen Chang[1, 2, 4‡], Jingsi Ming[5], Olga Botvinnik[6], Lolita Penland[6], Alexander Tarashansky[6, 7], Antoine de Morree[8, 9], Kyle J. Travaglini[1, 2], Jia Zhao[10], Gefei Wang[10], Kazuteru Hasegawa[1, 11, 12], Hosu Sin[13], Rene Sit[6], Jennifer Okamoto[6], Rahul Sinha[14], Yue Zhang[2, 15], Caitlin J. Karanewsky[1, 2], Jozeph L. Pendleton[1, 2], Maurizio Morri[6], Martine Perret[16], Fabienne Aujard[16], Lubert Stryer[17], Steven Artandi[1, 11, 12], Margaret T. Fuller[13, 18], Irving L. Weissman[14], Thomas A. Rando[8], James E. Ferrell, Jr.[1, 3], Bo Wang[7], Iwijn De Vlaminck[19], Can Yang[10], Kerriann M. Casey[20], Megan A. Albertelli[20], Angela Oliveira Pisco[6], Jim Karkanias[6], Norma Neff[6], Angela Ruohao Wu[21, 22, 23], Stephen R. Quake[6, 7, 24]†, Mark A. Krasnow[1, 2]†

‡ Co-first authors
† Corresponding authors: krasnow@stanford.edu (M.A.K.), steve@quake-lab.org (S.R.Q.)

SUPPLEMENTARY INFORMATION

Table of contents

Supplementary Notes (pages 3-6)
Tabula Microcebus Consortium author contributions

Supplementary Figures (pages 7-19)
1. Full dendrogram of cell type designations in mouse lemur atlas
2. Dendrogram of cell types displayed across each organs separately
3. UMAP and differential expressed genes of additional previously unknown cell types
4. Expression patterns of example genes showing evolutionarily conserved or divergent expression patterns across human, lemur, and mouse
5. Fluorescent Activated Cell Sorting (FACS) gating strategy for SS2 library preparation

Supplementary Table Legends (pages 20-22)
1. Marker genes from human and/or mouse used to annotate the lemur cells from the atlas
2. Number of cells and cell types sequenced per individual and tissue
3. Differentially-expressed genes for each mouse lemur molecular cell type
4. Datasets and molecular cell types analyzed across species
5. One-to-one orthologous genes across human, lemur, mouse
6. Genes with a species conserved, primate-enriched, or primate-depleted expression for each molecular cell type across human, lemur, and mouse
7. Global patterns of gene expression conservation across human, lemur, and mouse
8. Gene set enrichment analysis of the genes showing a highly-conserved expression pattern
9. Complete blood count (CBC) and other parameters of lemurs at time of euthanasia

Supplementary Methods (pages 23-37)
Tissue procurement
Histopathological image repository
Preparation of single cell suspensions and FACS-sorting for scRNA-seq
Library preparation, quality control, and sequencing
Genome alignment
Cell filtering
FIRM integration
Comparison of expression profiles among lemur cell types: calculating differentially expressed genes
Tissue Dissociation Protocols

Supplementary References (page 38)

Supplementary Notes

The Tabula Microcebus Consortium authorship contributions

Lead authors

Camille Ezran[1, 2‡], Shixuan Liu[1, 2, 3‡], Stephen Chang[1, 2, 4‡], Jingsi Ming[5], Olga Botvinnik[6], Lolita Penland[6], Alexander Tarashansky[6, 7], Antoine de Morree[8, 9], Kyle J. Travaglini[1, 2], Jia Zhao[10], Gefei Wang[10], Kazuteru Hasegawa[1, 11, 12], Hosu Sin[13], Rene Sit[6], Jennifer Okamoto[6], Rahul Sinha[14], Yue Zhang[2, 15], Caitlin J. Karanewsky[1, 2], Jozeph L. Pendleton[1, 2], Maurizio Morri[6], Martine Perret[16], Fabienne Aujard[16], Lubert Stryer[17], Steven Artandi[1, 11, 12], Margaret T. Fuller[13, 18], Irving L. Weissman[14], Thomas A. Rando[8], James E. Ferrell, Jr.[1, 3], Bo Wang[7], Iwijn De Vlaminck[19], Can Yang[10], Kerriann M. Casey[20], Megan A. Albertelli[20], Angela Oliveira Pisco[6], Jim Karkanias[6], Norma Neff[6], Angela Ruohao Wu[21, 22, 23], Stephen R. Quake[6, 7, 24]†, Mark A. Krasnow[1, 2]†

‡ Co-first authors

† Corresponding authors: krasnow@stanford.edu (M.A.K.), steve@quake-lab.org (S.R.Q.)

Leadership and coordination

Trainees: Camille Ezran[1, 2], Shixuan Liu[1, 2, 3], Stephen Chang[1, 2, 4]

Principal investigators: Mark A. Krasnow[1, 2], Stephen R. Quake[6, 7, 24], Angela Ruohao Wu[21, 22, 23], Norma Neff[6], Jim Karkanias[6], Angela Oliveira Pisco[6]

Donor recruitment and animal husbandry

Megan A. Albertelli[20], Caitlin J. Karanewsky[1, 2], Jozeph L. Pendleton[1, 2], Fabienne Aujard[16], Martine Perret[16], Liza Shapiro[25], Andriamahery Razafindrakoto[26], Hajanirina Noëline Ravelonjanahary[26], Patricia Wright[27], Anne D. Yoder[28], Cathy V. Williams[29], Robert Schopler[29], Ute Radespiel[30], Jean-Michel Verdier[31], Corinne Lautier[31], E. Christopher Kirk[25], Rebecca Lewis[25]

Necropsy, sample collection, biobanking, histology, and pathology

Kerriann M. Casey[20], Megan A. Albertelli[20], Caitlin J. Karanewsky[1, 2], Jozeph L. Pendleton[1, 2], Camille Ezran[1, 2], Shixuan Liu[1, 2, 3], Kyle J. Travaglini[1, 2], Astrid Gillich[1, 2], Zicheng Zhao[2, 20], Stephen Chang[1, 2, 4], Elias Godoy[20], Jérémy Terrien[16], Jacques Epelbaum[16, 32], Dita Gratzinger[33], Katherine Lucot[33], Thomas Montine[33]

Tissue processing, annotation, and analysis [Principal investigators]

Aorta: Jessica D'Addabbo[4, 34], Isaac Bakerman[4], [Patricia Nguyen[4, 12, 34]]

Bladder: Aaron Kershner[1, 14], Karim Mrouj[14], [Philip Beachy[3, 13, 14, 35]]

Blood: Rahul Sinha[14], Camille Ezran[1, 2], Yue Zhang[2, 15], Shixuan Liu[1, 2, 3], Caitlin J. Karanewsky[1, 2], [Irving L. Weissman[14], Mark A. Krasnow[1, 2]]

Bone (limb, spine): Thomas H. Ambrosi[14], Malachia Hoover[14], Alina Alam[14], [Charles Chan[14]]

Bone marrow: Rahul Sinha[14], SoRi Jang[1, 2], Camille Ezran[1, 2], [Irving L. Weissman[14], Mark A. Krasnow[1, 2]]

Brain (cortex, brainstem, cerebellum): Avin Veerakumar[1, 2, 7], Peng Li[1, 2], Andrea R. Yung[1, 2], Connor V. Duffy[2, 18], Song-Lin Ding[36], [Ed S. Lein[36], Silvana Konermann[1,2], Liqun Luo[2, 15], Trygve E. Bakken[36], Justus M. Kebschull[37], Rebecca D. Hodge[36], Mark A. Krasnow[1, 2]]

Colon/Small intestine: Taichi Isobe[38], [Michael F. Clarke[14]]

Diaphragm/Limb muscle: Antoine de Morree[8, 9], Biter Bilen[8], Jean Farup[8, 9], Andoni Urtasun[8], Jengmin Kang[8], [Thomas A. Rando[8]]

Eye: Ming Chen[18], BaoXiang Li[39], Varun Ramanan Subramaniam[39], Shravani Mukherjee[39], Aditi Swarup[39], Lily Kim[18], Bronwyn Scott[39], Ahmad Al-Moujahed[39], [Albert Y. Wu[39], Douglas Vollrath[18], Lubert Stryer[17]]

Fat: Nicholas Schaum[8], Jingsi Ming[5], Shixuan Liu[1, 2, 3], Amanda L. Wiggenhorn[33, 40], [Tony Wyss-Coray[8, 41], Jonathan Z. Long[33, 42], Angela Ruohao Wu[21, 22, 23]]

Ganglion (vagus nerve): Yin Liu[1, 2], [Mark A. Krasnow[1, 2]]

Heart: Stephen Chang[1, 2, 4], [Mark A. Krasnow[1, 2]]

Hypothalamus & Pituitary: Andrea R. Yung[1, 2], Shixuan Liu[1, 2, 3], [Mark A. Krasnow[1, 2]]

Kidney: Shixuan Liu[1, 2, 3], Ahmad Nabhan[1, 2], Lolita Penland[6], Yue Zhang[2, 15], Andrea R. Yung[1, 2], [Gabriel Loeb[43], Mark A. Krasnow[1, 2]]

Liver: Shengda Lin[44], Shixuan Liu[1, 2, 3], Honor Paine[45], Deviana Burhan[45], Aris Taychameekiatchai[45], [Steven Artandi[1, 11, 12], Bruce Wang[45]]

Lung: Kyle J. Travaglini[1, 2], Ahmad Nabhan[1, 2], F. Hernán Espinoza[1, 2], Astrid Gillich[1, 2], [Christin Kuo[46], Ross Metzger[4, 46], Mark A. Krasnow[1, 2]]

Lymph node: SoRi Jang[1, 2], Lolita Penland[6], [Norma Neff[6], Mark A. Krasnow[1, 2]]

Mammary gland: Zhen Qi[14], [Michael F. Clarke[14]]

Microbiome: Rebecca Culver[18], [Kerwyn C. Huang[6, 7, 47]]

Pancreas: Patrick Neuhöfer[1, 11, 12], Charles A. Chang[13, 48], Yan Hang[13, 48], [Seung K. Kim[12, 13, 48, 49], Steven Artandi[1, 11, 12]]

Prostate: Hannah N. W. Weinstein[50, 51, 52], Paul Allegakoen[50], [Franklin W. Huang[50]]

Salivary gland: Sivakamasundari V[14], [Philip Beachy[3, 13, 14, 35]]

Skin: Song Eun Lee[8, 41], [Tony Wyss-Coray[8, 41]]

Spleen: Lolita Penland[6], Hannah K. Frank[33, 53], Dita Gratzinger[33], [Norma Neff[6], Scott D. Boyd[33]]

Testes: Kazuteru Hasegawa[1, 11, 12], Hosu Sin[13], Jingsi Ming[5], [Steven Artandi[1, 11, 12], Margaret T. Fuller[13, 18], Angela Ruohao Wu[21, 22, 23]]

Thymus: Isaac Bakerman[4], Jessica D'Addabbo[4, 34], [Patricia Nguyen[4, 12, 34]]

Tongue: Wan-Jin Lu[14], Ankit Baghel[15], [Philip Beachy[3, 13, 14, 35]]

Trachea/Thyroid/Parathyroid: William Kong[14], [Philip Beachy[3, 13, 14, 35]]

Uterus/Abdominal mass: Lolita Penland[6], [Norma Neff[6]]

Library processing, sorting, and sequencing

Stephen Chang[1, 2, 4], Lolita Penland[6], Carly Israel[6], Rene Sit[6], Jennifer Okamoto[6], Ashley Maynard[6], Michelle Tan[6], Yue Zhang[2, 15], Rahul Sinha[14], Shixuan Liu[1, 2, 3], Camille Ezran[1, 2], Kyle J. Travaglini[1, 2], Antoine de Morree[8, 9], Ahmad Nabhan[1, 2], Youcef Ouadah[1], Jalal Baruni[1, 2, 15, 54], Timothy Ting-Hsuan Wu[1, 2], Robert C. Jones[8], Norma Neff[6], Maurizio Morri[6], Spyros Darmanis[6], Sheela Crasta[6], Jia Yan[6], Aditi Agrawal[6], Shelly Huynh[6], Brian Yu[6], James Webber[6]

Global data analysis [Principal investigators]

General analysis: Camille Ezran[1, 2], Shixuan Liu[1, 2, 3], [Mark A. Krasnow[1, 2]]

Sequence alignment: Stephen Chang[1, 2, 4], Kyle J. Travaglini[1, 2], Rahul Sinha[14], Rene Sit[6], [Mark A. Krasnow[1, 2], Irving L. Weissman[14], Stephen R. Quake[6, 7, 24]]

Integration algorithms: Jingsi Ming[5], Jia Zhao[10], Gefei Wang[10], [Angela Ruohao Wu[21, 22, 23], Can Yang[10]]

Molecular trajectory: Shixuan Liu[1, 2, 3], Camille Ezran[1, 2], Timothy Ting-Hsuan Wu[1, 2], Weilun Tan[6], [Mark A. Krasnow[1, 2]]

Global cell type comparison: Shixuan Liu[1, 2, 3], [Mark A. Krasnow[1, 2]]

Cross-species comparison: Shixuan Liu[1, 2, 3], Camille Ezran[1, 2], Jia Zhao[10], Gefei Wang[10], Alexander Tarashansky[6, 7], Olga Botvinnik[6], Saba Nafees[6], Zhengda Li[3], Kyle J. Travaglini[1, 2], Antoine de Morree[8, 9], [Mark A. Krasnow[1, 2], Angela Ruohao Wu[21, 22, 23], Can Yang[10], James E. Ferrell, Jr.[1, 3], Jim Karkanias[6], Bo Wang[7], Stephen R. Quake[6, 7, 24], Thomas A. Rando[8]]

Spermatogenesis: Jingsi Ming[5], Hosu Sin[13], Kazuteru Hasegawa[1, 11, 12], Camille Ezran[1, 2], Shixuan Liu[1, 2, 3], [Angela Ruohao Wu[21, 22, 23], Margaret T. Fuller[13, 18], Steven Artandi[1, 11, 12], Can Yang[10], Mark A. Krasnow[1, 2]]

uTAR (accompanying paper): Michael F.Z. Wang[19, 55], [Iwijn De Vlaminck[19]]

SICILIAN (accompanying paper): Roozbeh Dehghannasiri[1, 56], Julia Olivieri[1, 57], [Julia Salzman[1, 56]]

SAMap: Alexander Tarashansky[6, 7], [Bo Wang[7]]

Major histocompatibility locus (accompanying paper): Lisbeth A. Guethlein[47, 58], Camille Ezran[1, 2], [Peter Parham[47, 58], Mark A. Krasnow[1, 2]]

B cell receptor locus (accompanying paper): Hannah K. Frank[33, 53], Camille Ezran[1, 2], [Scott D. Boyd[33], Mark A. Krasnow[1, 2]]

Cross-tissue neutrophil trajectory (accompanying paper): Camille Ezran[1, 2], Shixuan Liu[1, 2, 3], [Mark A. Krasnow[1, 2]]

Cross-tissue monocyte and macrophage comparison (accompanying paper): Qiuyu Jing[21], Camille Ezran[1, 2], Shixuan Liu[1, 2, 3], [Angela Ruohao Wu[21, 22, 23], Mark A. Krasnow[1, 2]]

Chemokine expression (accompanying paper): Camille Ezran[1, 2], [Mark A. Krasnow[1, 2]]

Metastatic tumor (accompanying paper): Jane Antony[14], Camille Ezran[1, 2], [Michael F. Clarke[14], Mark A. Krasnow[1, 2]]

Endothelium: Camille Ezran[1, 2], Astrid Gillich[1, 2], Geoff Stanley[7], [Ross Metzger[4, 46], Mark A. Krasnow[1, 2], Stephen R. Quake[6, 7, 24]]

Adipocytes (accompanying paper): Jinxurong Yang[21], Jingsi Ming[5], Camille Ezran[1, 2], Shixuan Liu[1, 2, 3], [Jonathan Z. Long[33, 42], Angela Ruohao Wu[21, 22, 23], Mark A. Krasnow[1, 2], Can Yang[10]]

Primate-specific/mouse-absent genes (accompanying paper): Winston Koh[59, 60], Camille Ezran[1, 2], Shixuan Liu[1, 2, 3], [Mark A. Krasnow[1, 2]]

Global hormone signaling (accompanying paper): Shixuan Liu[1, 2, 3], [James E. Ferrell, Jr.[1, 3], Mark A. Krasnow[1, 2]]

OnClass: [Sheng Wang[61]]

Data portal/Online histology atlas: Zicheng Zhao[2, 20], Snigdha Agarwal[6], Kyle Awayan[6], Erin McGeever[6], Venkata N. P. Vemuri[6], [Stephen R. Quake[6, 7, 24], Angela Oliveira Pisco[6]]

Natural mutations (accompanying paper): Shixuan Liu[1, 2, 3], Stephen Chang[1, 2, 4], Pranav V. Lalgudi[1, 18], [Mark A. Krasnow[1, 2]]

Principal investigators (A-Z)

Megan A. Albertelli[20], Steven Artandi[1, 11, 12], Fabienne Aujard[16], Trygve E. Bakken[36], Philip Beachy[3, 13, 14, 35], Scott D. Boyd[33], Kerriann M. Casey[20], Charles Chan[14], Michael F. Clarke[14], Jacques Epelbaum[16, 32], James E. Ferrell, Jr.[1, 3], Margaret T. Fuller[13, 18], Dita Gratzinger[33], Franklin W. Huang[50], Kerwyn C. Huang[6, 7, 47], Jim Karkanias[6], Justus M. Kebschull[37], Seung K. Kim[12, 13, 48, 49], Silvana Konermann[1,2], Mark A. Krasnow[1, 2], Christin Kuo[46], Ed S. Lein[36], Shengda Lin[44], Gabriel Loeb[43], Jonathan Z. Long[33, 42], Liqun Luo[2, 15], Ross Metzger[4, 46], Jingsi Ming[5], Thomas Montine[33], Norma Neff[6], Patricia Nguyen[4, 12, 34], Peter Parham[47, 58], Martine Perret[16], Angela Oliveira Pisco[6], Stephen R. Quake[6, 7, 24], Thomas A. Rando[8], Ute Radespiel[30], Julia Salzman[1, 56], Liza Shapiro[25], Lubert Stryer[17], Jérémy Terrien[16], Jean-Michel Verdier[31], Iwijn De Vlaminck[19], Douglas Vollrath[18], Bo Wang[7], Bruce Wang[45], Sheng Wang[61], Irving L. Weissman[14], Patricia Wright[27], Tony Wyss-Coray[8, 41], Albert Y. Wu[39], Angela Ruohao Wu[21, 22, 23], Can Yang[10], Anne D. Yoder[28]

Writing

Camille Ezran[1, 2,] Shixuan Liu[1, 2, 3], Stephen Chang[1, 2, 4], Jingsi Ming[5], Antoine de Morree[8, 9], Lubert Stryer[17], Angela Ruohao Wu[21, 22, 23], Stephen R. Quake[6, 7, 24], Mark A. Krasnow[1, 2]

Supplementary Figures

Supplementary Figure 1. Full dendrogram of cell type designations in mouse lemur atlas. Expansion of Fig. 1e showing full dendrogram of the 256 assigned designations for the 768 molecular cell type across the atlas. Designations are arranged by compartment (epithelial, endothelial, stromal, immune (lymphoid/hematopoietic/myeloid), neural, germ) and then ordered by tissue system (epithelial compartment) or biological relatedness (other compartments). Full name for designations in Fig. 1e. Unknown or previously uncharacterized molecular types in bold. PF, proliferative cell state; H1-12, hybrid cell types; M1-7, mixed clusters of distinct cell types too few to assign separately; (), tissue abbreviation (full name in box) indicated for cell types present in ≤3 tissues; *, molecular types identified in only one individual; †, tumor cells[1].

Supplementary Figure 2. Dendrogram of cell types displayed across each organs separately.
a-z, aa. Dendrograms of atlas molecular cell types as in Supplementary Fig. 1, separated by organ: **a**, brain cortex; **b**, brainstem; **c**, hypothalamus/pituitary gland; **d**, eye retina; **e**, skin; **f**, fat; **g**, mammary gland; **h**, limb muscle; **i**, diaphragm; **j**, bone; **k**, bone marrow; **l**, blood; **m**, spleen; **n**, thymus; **o**, heart; **p**, aorta; **q**, lung; **r**, trachea; **s**, tongue; **t**, small intestine; **u**, colon; **v**, liver; **w**, pancreas; **x**, kidney; **y**, bladder; **z**, uterus; **aa**, testes. Cell designations in gray indicate possible tissue contaminants. Full name for designations in Fig. 1e.

Supplementary Figure 3. UMAP and differential expressed genes of additional previously unknown cell types.
UMAPs **(a, c, f)** of indicated tissue compartments in parentheses with unknown molecular type highlighted (dashed circles), and dot plots (**b, d, g, h**) of genes differentially expressed in the unknown cell type (dashed box) vs. other cell types in the dot plot, formatted as in Extended Data Fig. 4.
a. Kidney stromal cells (L2, L3, and L4, 10x) with unknown stromal cell type (*ST6GAL2+,* #143). Corresponding dot plot in Fig. 3f.
b. Dot plot showing expression of indicated marker genes and DEGs in the unknown population (*ST6GAL2+,* #143) plus kidney epithelial, endothelial and other stromal cell types for comparison.
c,d. Gonadal fat adipocyte tissue (GAT) epithelial and stromal cells (L2, 10x) with unknown epithelial cell type (*CRISP3+,* #15).
e. Dot plot as above showing expression of indicated marker genes and DEGs in the unknown population (*CRISP3+*, #15) in GAT, plus urothelial and mesothelial cells from all tissues (L1-L4, 10x), which share expression of some DEGs from #15. Fibroblasts included for comparison.
f,g. Blood immune cells (L2 and L4, SS2) with unknown epithelial cell type (*PGAP1+,* #16).
h. Dot plot as above showing expression of indicated marker genes and DEGs in the unknown epithelial population (*PGAP1+*, #16) in blood, plus astrocytes, oligodendrocyte precursor cells (OPC), oligodendrocytes, and ependymal cells from all tissues (L1-L4, SS2), which express high levels of the DEGs from #16.
[], description of genes identified by NCBI as a loci: *[uncharacterized 1], LOC109730246; [uncharacterized 2], LOC105861777; [TTC39B], LOC105884518.*

Supplementary Figure 4. Expression patterns of example genes showing evolutionarily conserved or divergent expression patterns across human, lemur, and mouse.
a-f. Dot plots formatted as in Fig. 5i showing expression across human, lemur and mouse in the 63 orthologous cell types of genes highly conserved across the three species (**a**), HL genes (**b**) with expression patterns similar in the two primates but divergent in mouse, which may underlie primate-specific biology, HM (**c**) and LM (**d**) genes which may underlie lemur- and human-specific biology, respectively, as well as genes that show overall moderately-conserved (**e**) and lowly-conserved (**f**) expression patterns across the species. Note HL genes do not show enrichment in specific GO terms, and their diverse functions span immune regulation (e.g., *CXCL16*, *CX3CR1*, *LBP*, *SH2B3*, *VSIG4*, *ISG20*, *CD82*), cell growth and proliferation (e.g., *CDK6*, *ABL1*, *POLE3*, *CCNY*, *S100A14*, *HAUS1*, *ARPP19*), protein synthesis and degradation (e.g., *ASXL1*, *ZNF467*, *ATOH8*, *MSI2*, *CSDE1*, *SIAH2*, *ASB9*, *CTSF*), and cell and hormonal signaling (e.g., *HHIP*, *DLK1*, *SFRP5*, *NKD1*, *NKD2*, *FGFR3*, *GFRA1*, *PRKCZ*, *VIPR1*; see also hormone atlas[2]), whereas some remain poorly characterized (e.g., *C19orf47*, *C11orf24*, *C10orf53*, *WDR38*, *RNF150*, *SH3YL1*, *FIBIN*, *TMEM51*, *AMMECR1*, *RUNDC3A*, *VMO1*, *HRCT1*, *FAM110A*) (Supplementary Table 7).

Supplementary Figure 5. Fluorescent Activated Cell Sorting (FACS) gating strategy for SS2 library preparation.
FACS plot showing gating strategy used to sort lemur blood single-cell suspension into individual wells for SS2 library preparation. The same strategy was used for all other tissues processed for SS2 (except for cardiomyocytes which were hand-picked). Left to right: forward scatter area vs. side scatter area (to exclude debris), Sytox blue dead stain vs. side scatter area (to exclude dead cells), forward scatter area vs. forward scatter height (to exclude doublets).

Supplementary Fig. 1

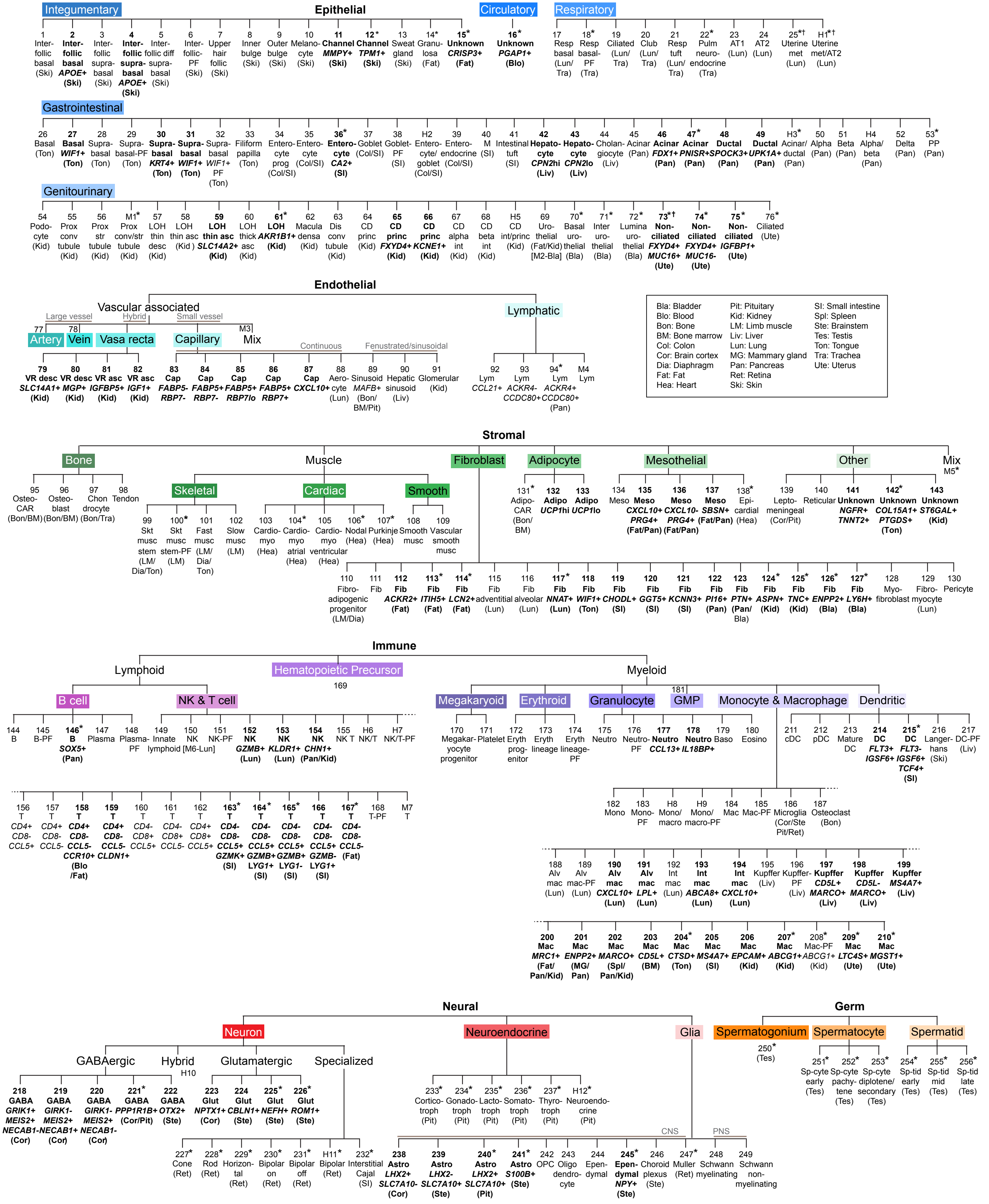

Supplementary Fig. 2

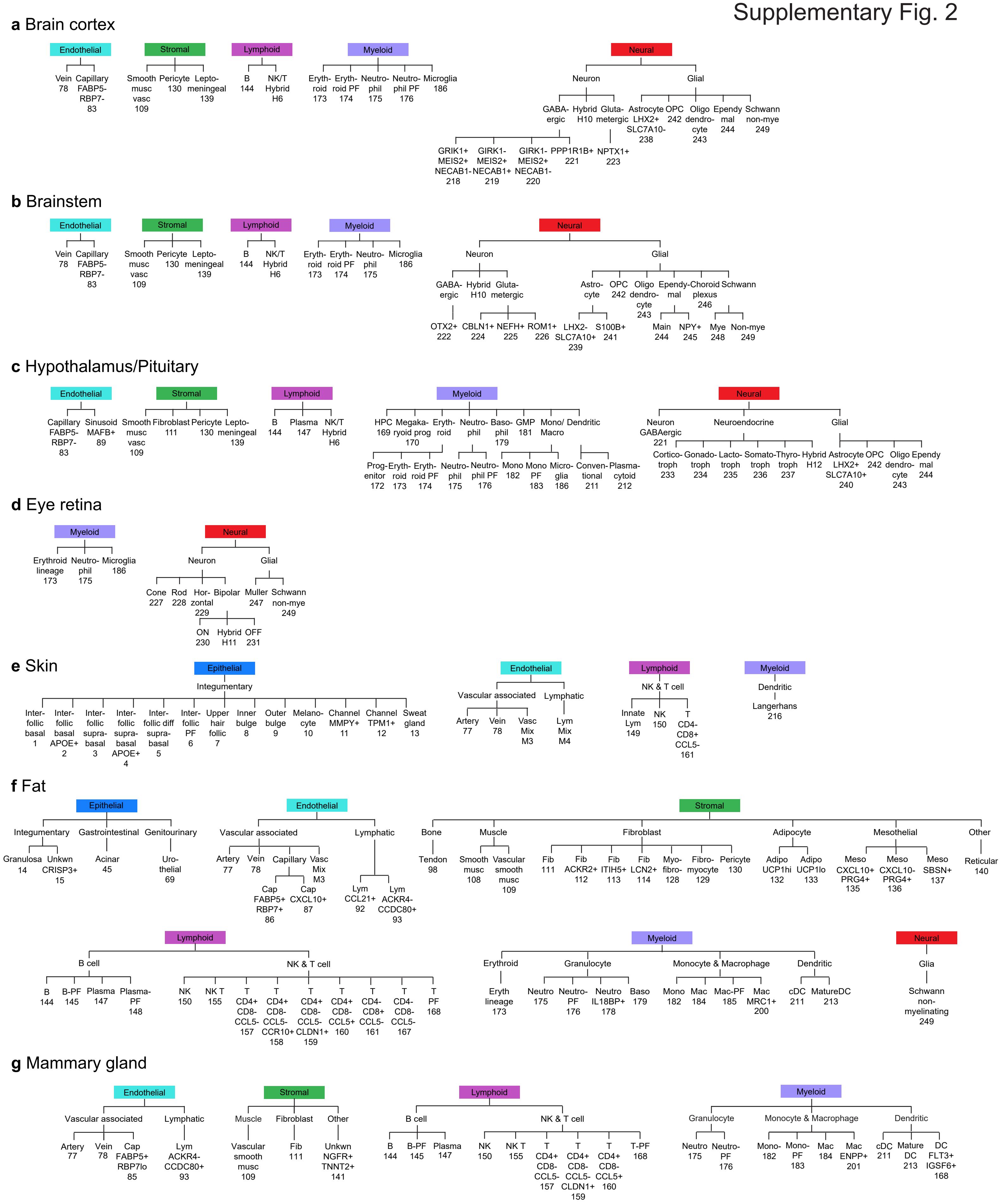

Supplementary Fig. 2
(p. 2)

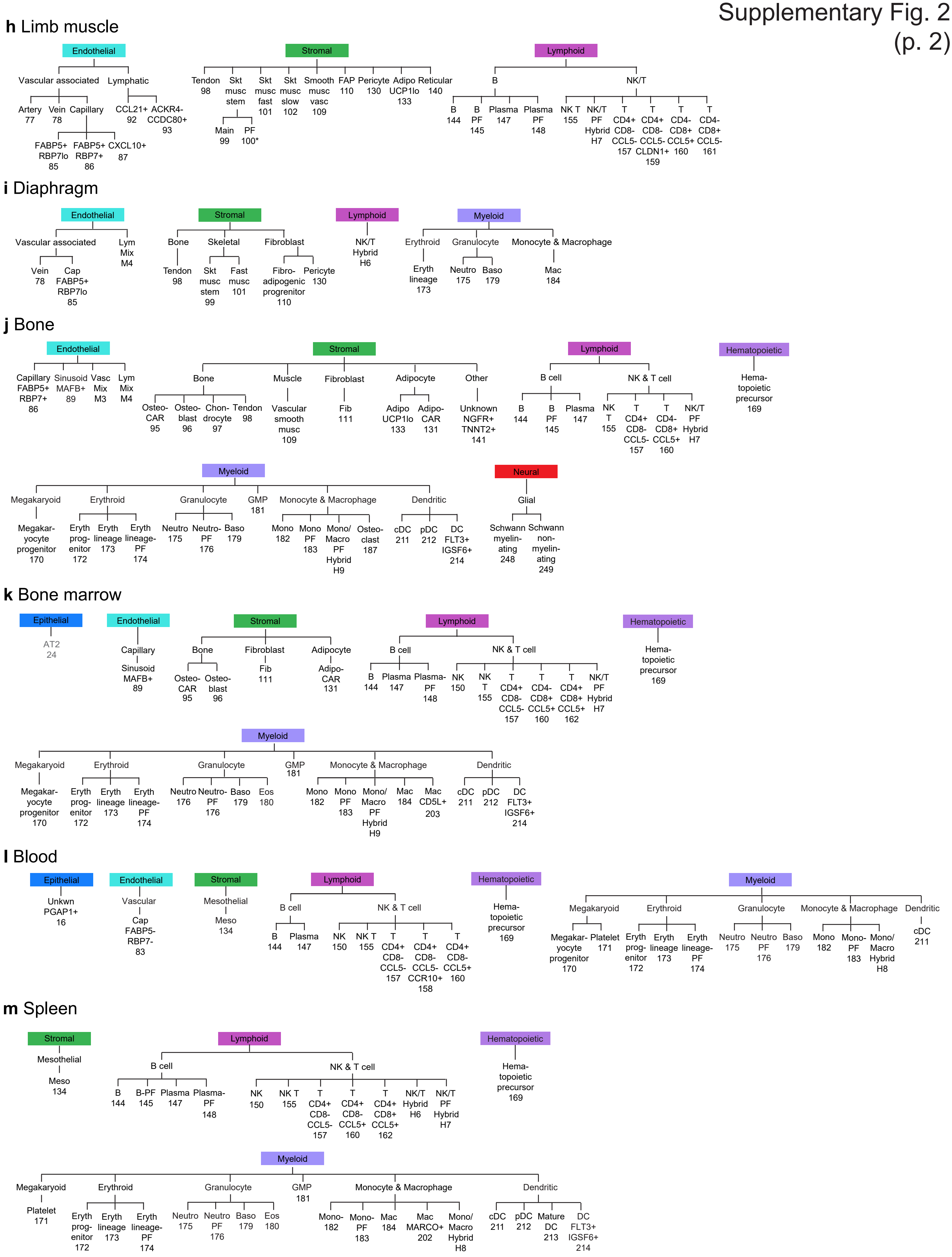

Supplementary Fig. 2
(p. 3)

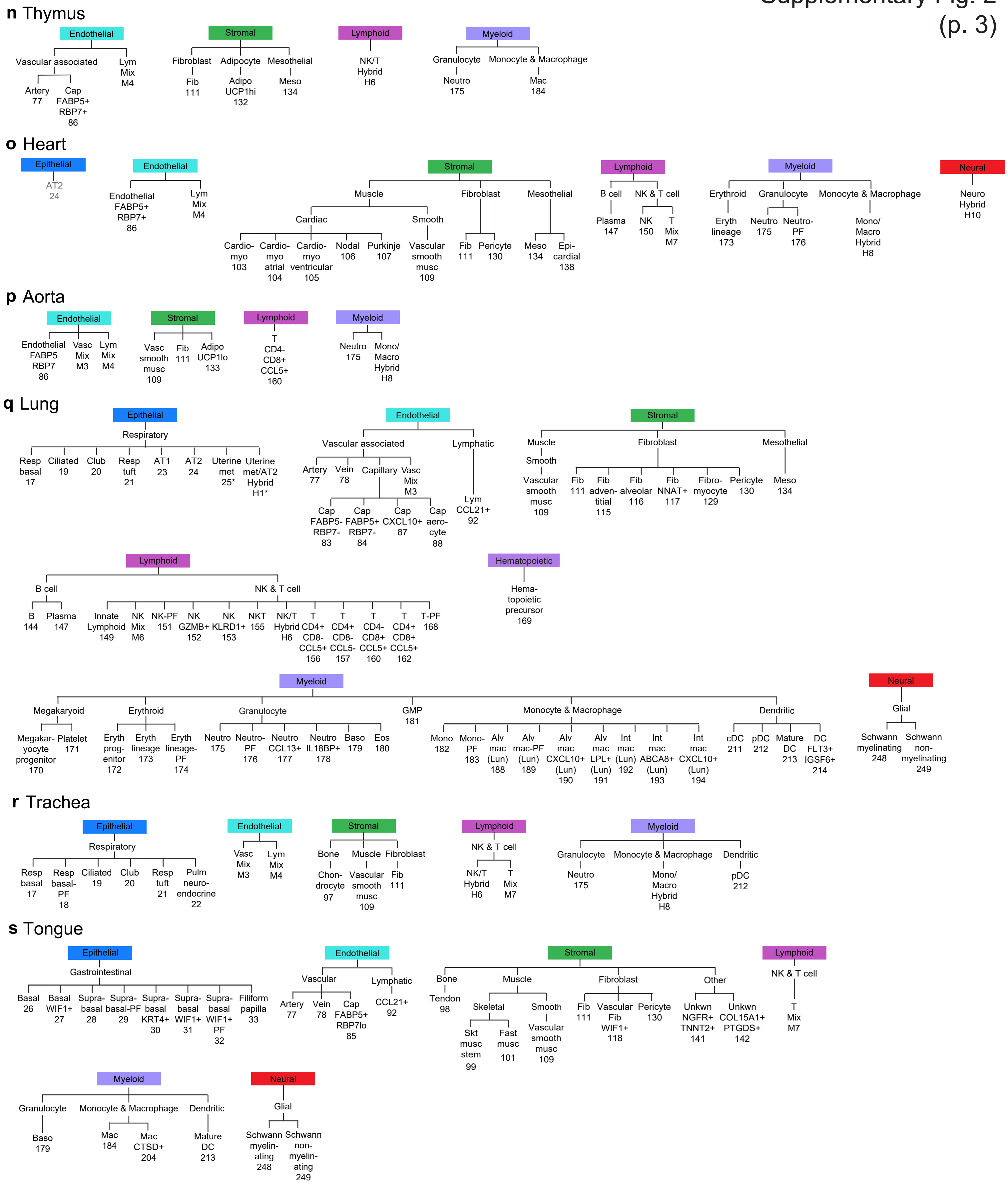

Supplementary Fig. 2
(p. 4)

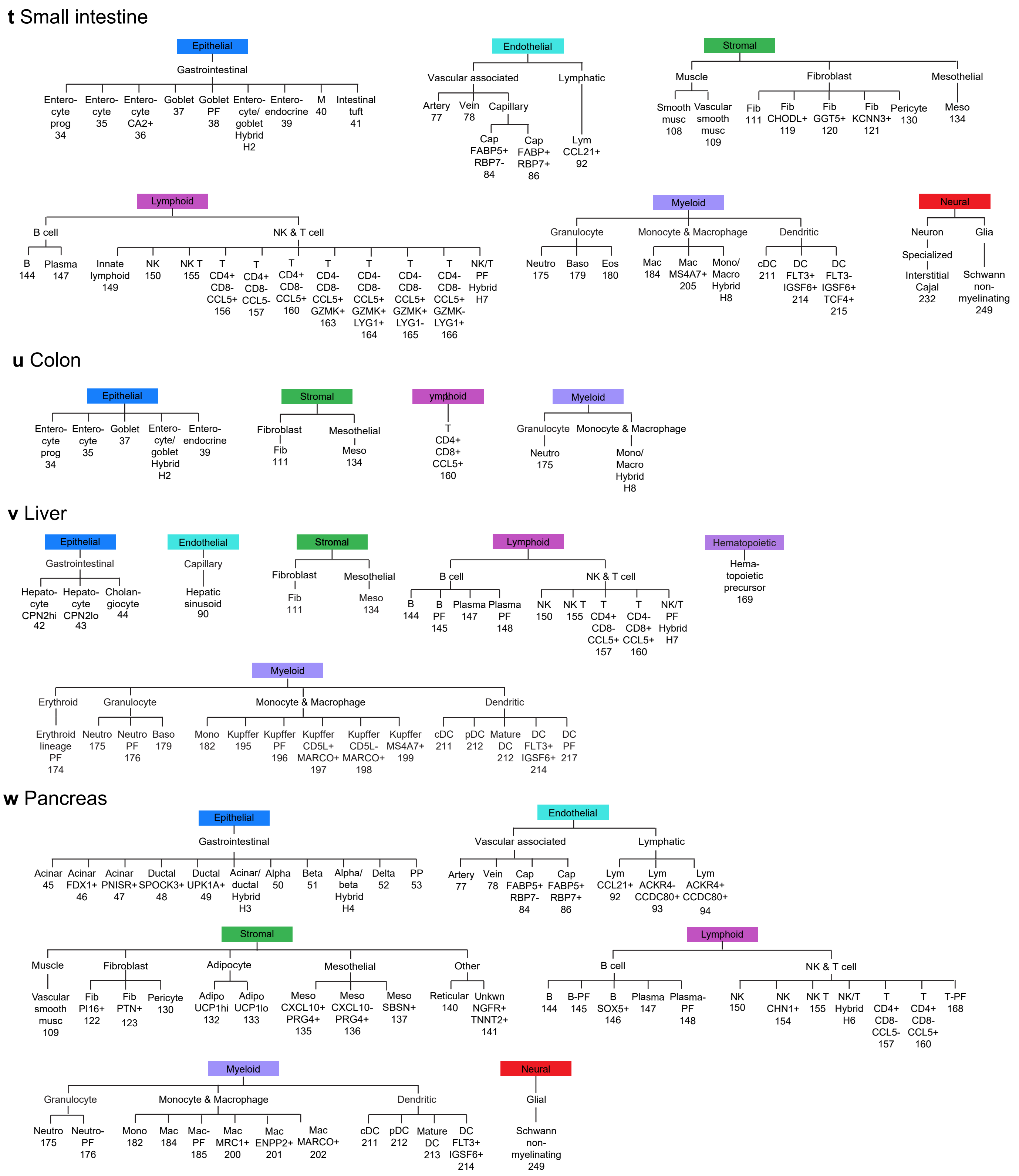

Supplementary Fig. 2
(p. 5)

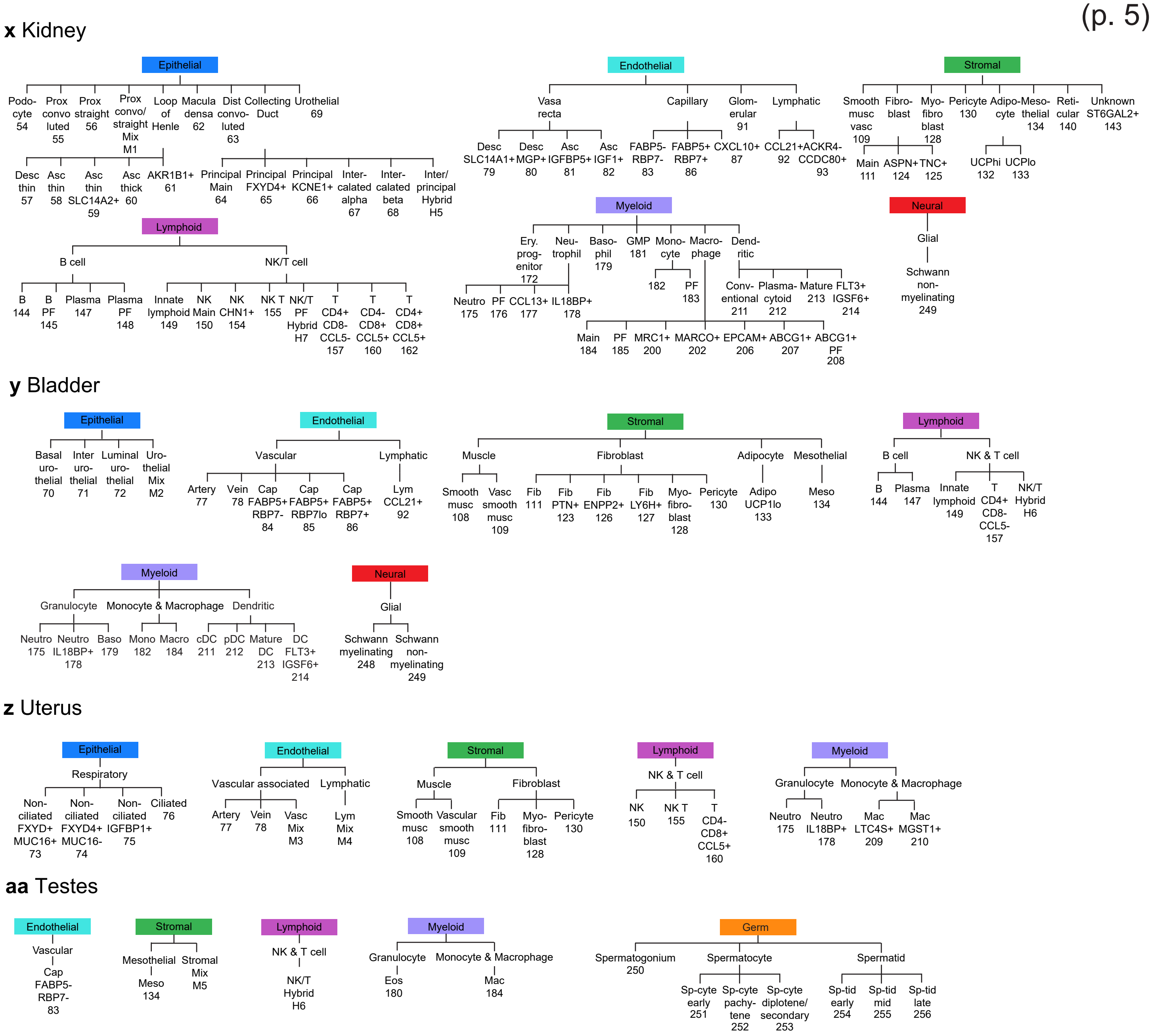

Supplementary Fig. 3

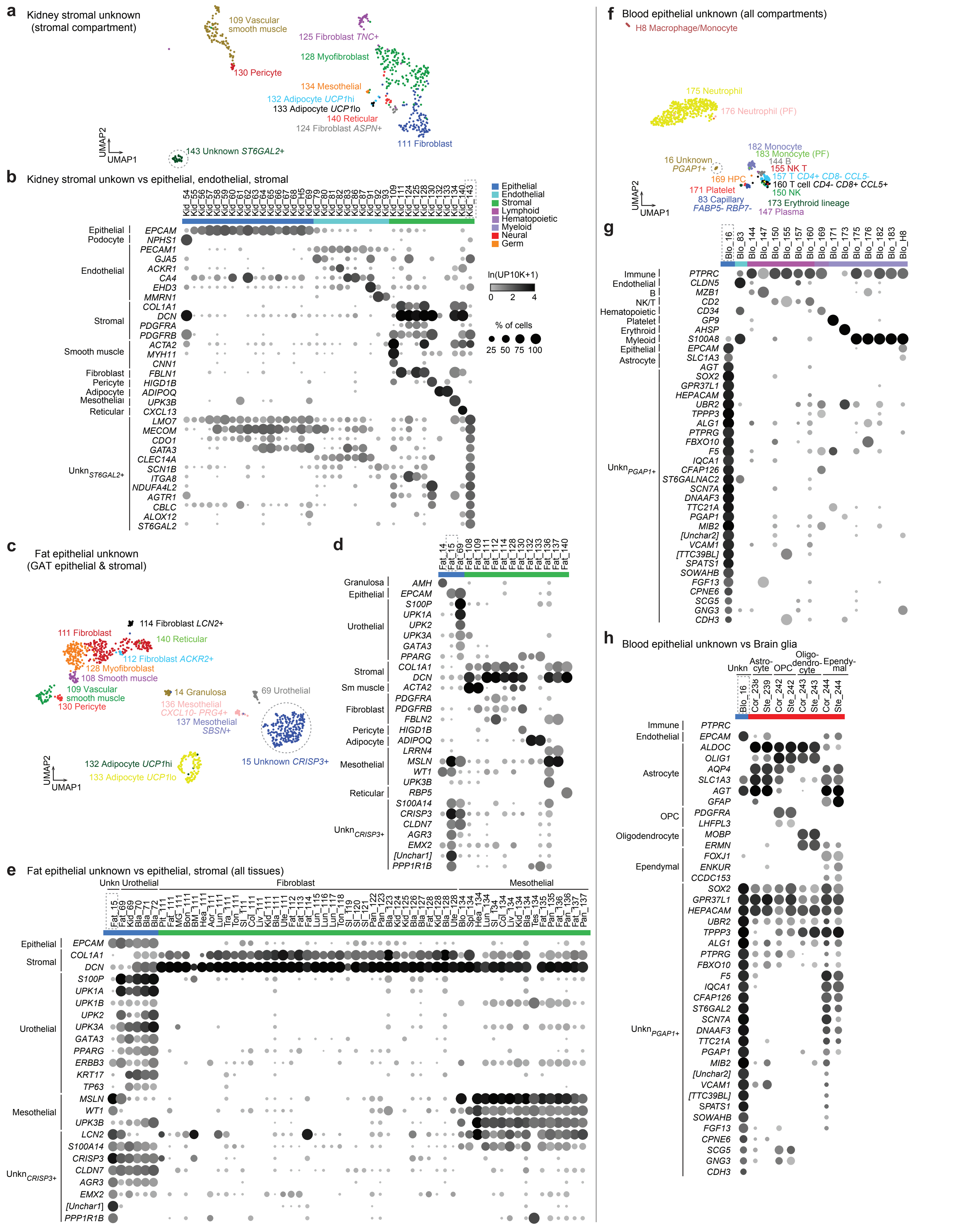

Supplementary Fig. 4

a Highly-conserved

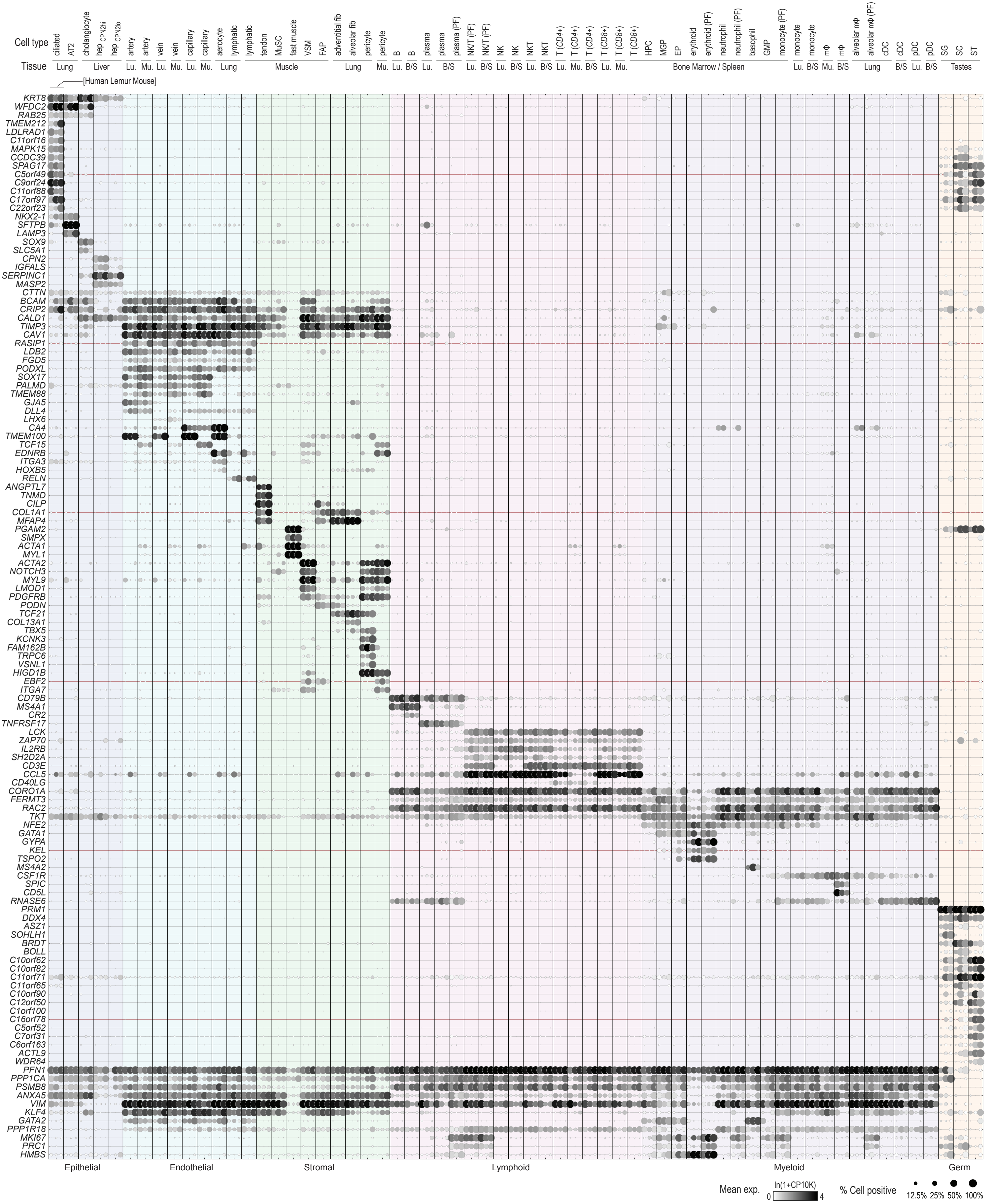

b HL-conserved

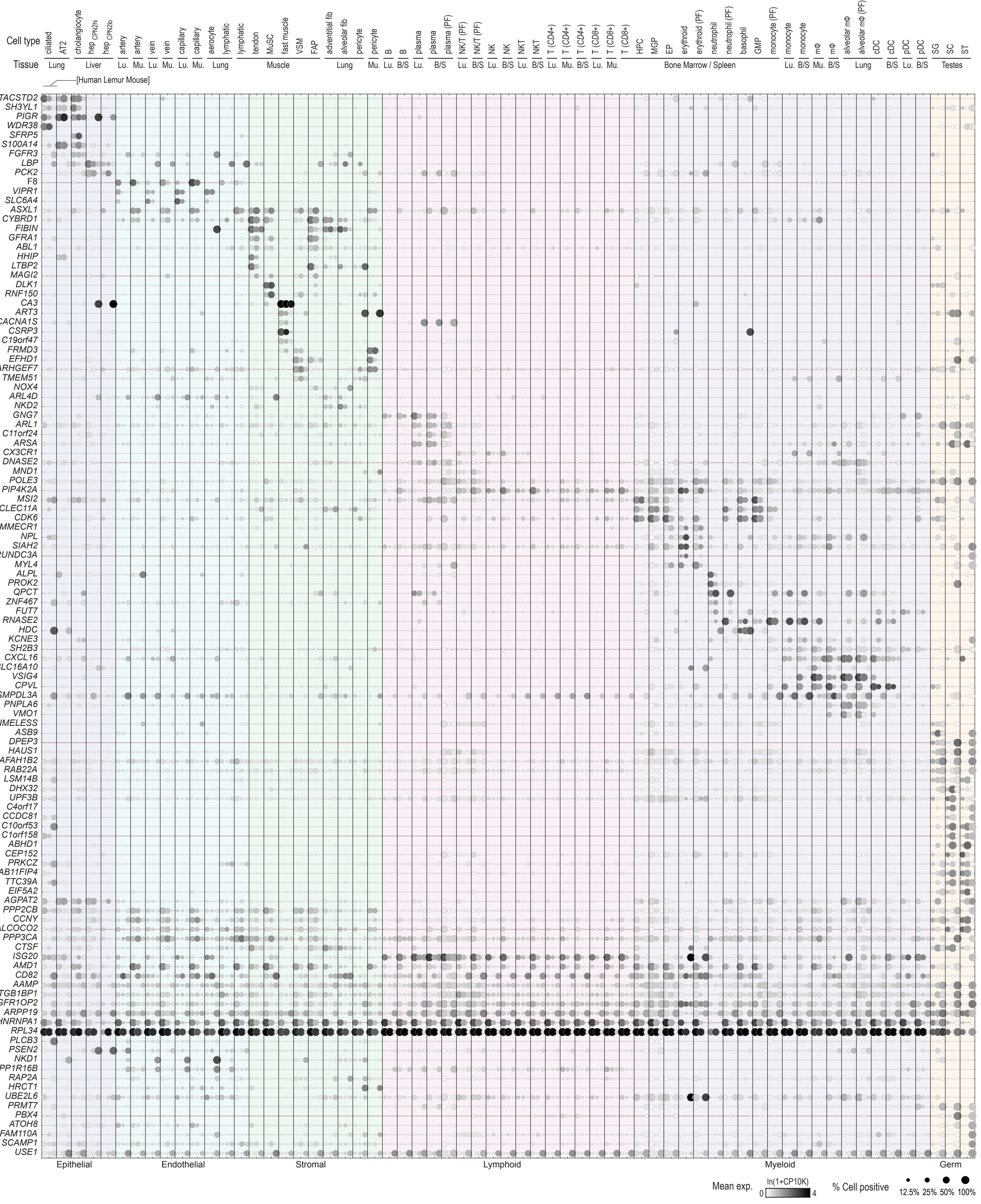

Supplementary Fig. 4
(p. 3)

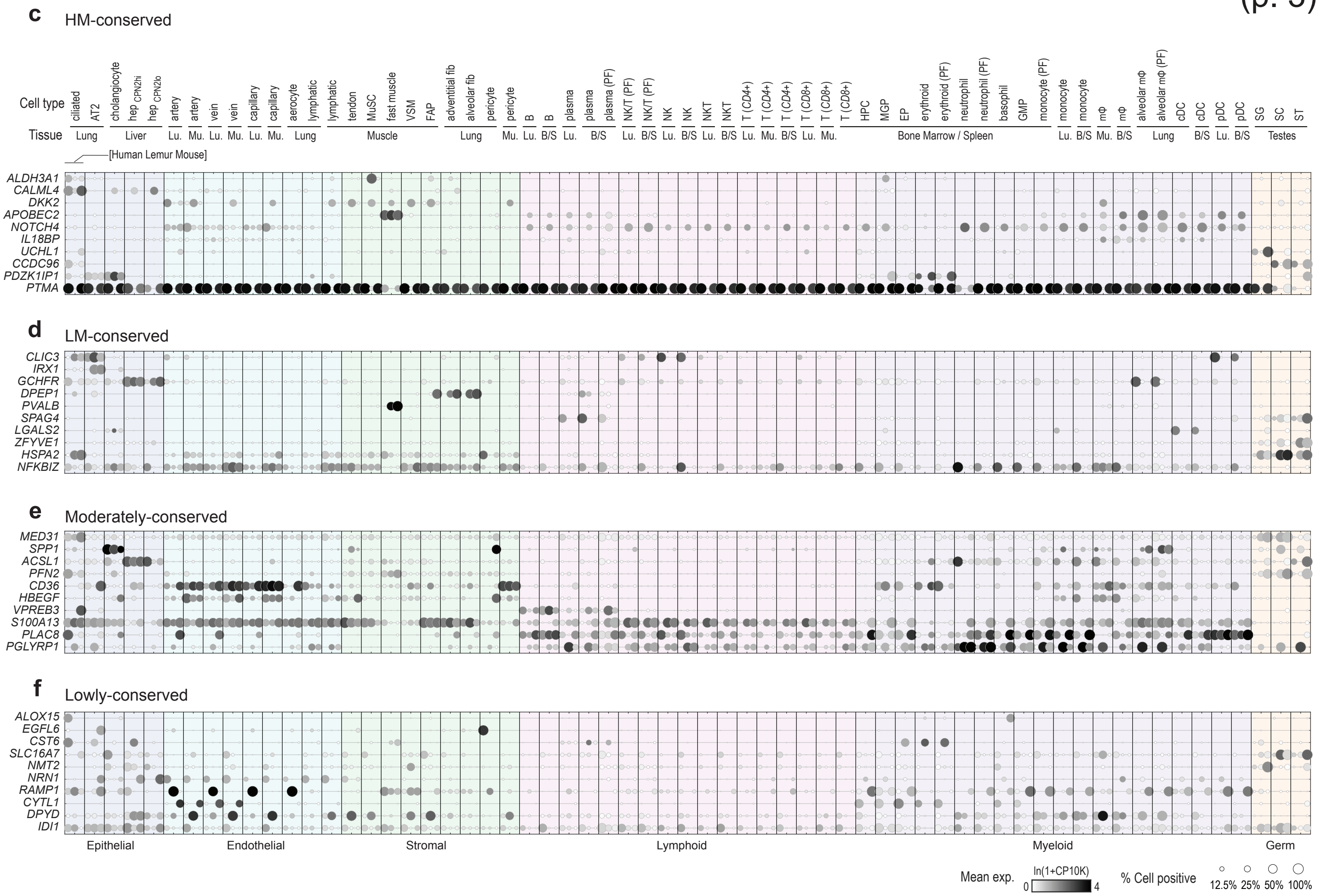

Supplementary Fig. 5

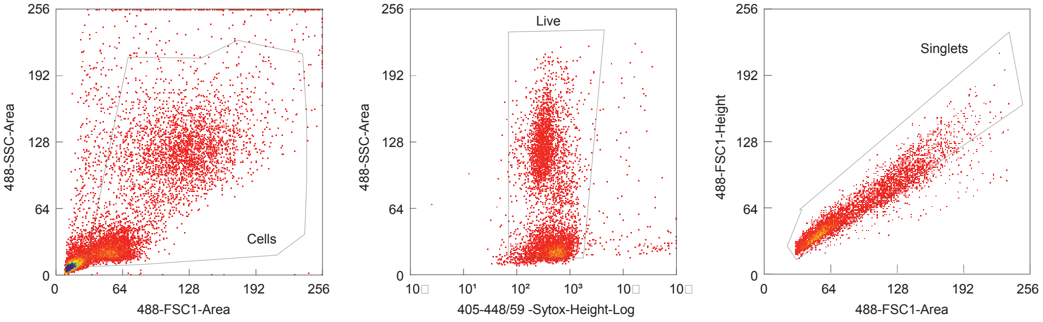

Region	Count	% All
Total	16478	100.00
Cells	6959	42.23

Region	Count	% All
Total	6959	42.23
Live	6417	38.94

Region	Count	% All
Total	6417	38.94
Singlet	5940	36.05

Supplementary Table Legends

Supplementary Table 1. Marker genes from human and/or mouse used to annotate the lemur cells from the atlas.
The table lists for each classically defined histological cell type (rows) the known marker genes from corresponding human and/or mouse cell types and the corresponding references. Marker genes are listed by their lemur symbols with notes in brackets (see "Marker gene key"). Cell types are ordered by source tissue with cell types that exist in multiple tissues on the top. For each cell type, the table also shows the corresponding cell ontology assignments, tissue source, compartment assignment, and whether it is identified in the atlas from this study. The "comments" column also gives details regarding the annotation of the cell type. LCA, lemur cell atlas.

Supplementary Table 2. Number of cells and cell types sequenced per individual and tissue.
The first sheet ("Summary") provides for each tissue (rows) and for all tissues combined (last row) the total number of cells, number of high quality cells (excluding cells labeled as doublets or low quality) in each individual separately and combined, mean gene count per high quality cell and standard deviation, mean sequencing read (Smart-seq2) or UMI count (10x) per high quality cell and standard deviation, and number of cell types (excluding cells labeled as mix, doublets or low quality). This information is provided for the 10x and SS2 datasets separately and combined. The subsequent sheets are labeled by tissue and provide a list of the cell types annotated in that tissue (including cells labeled as doublets, mix, hybrid, and low quality) with the corresponding assigned tissue system (organized as in Fig. 1e), compartment, and cell ontology designation, dendrogram_annotation_number, order__compartment_freeannotation_tissue, order__tissue_compartment_freeannotation (variables are explained online at the Tabula Microcebus portal, see "Data" tab). The number of cells in each cell type are provided for the 10x and SS2 datasets separately and combined, as well as for each individual separately and combined.

Supplementary Table 3. Differentially-expressed genes for each mouse lemur molecular cell type.
Lists of the top 300 significant (adjusted *p*-value<0.05) differentially-expressed genes (DEGs) detected for each cell type in the 10x dataset (≥5 cells) using four different comparisons: vs. all other cell types in the atlas ("Table 3a DEG_atlas-wide"), vs. all other cell types from the same compartment across the atlas ("Table 3b DEG_atlas-compartment-wide"), vs. all other cell types from the same tissue (sheet "Table 3c DEG_tissue-wide"), or vs. all other cell types from the same compartment of that tissue (sheet "Table 3d DEG_tissue-compartment-wide"). For each cell type, three columns are included: gene symbol (ordered with ascending *p*-values), adjusted *p*-value, and log2-transformed fold-change of the DEGs. Columns of cell types with fewer than 5 10x cells or with no significant DEGs are left blank.

Supplementary Table 4. Datasets and molecular cell types analyzed across species.
The first sheet ("datasets") lists the datasets used in the cross-species comparison, separately for each species and tissue type, as well as metadata showing the individuals and number of high-quality cells analyzed.

The second sheet ("cell types") lists the molecular cell types analyzed in the cross-species comparisons with columns indicating their tissue group, compartment, unified cell type annotations, as well as number of cells from each species, and whether the cell types were analyzed in the 3-species (human, lemur, mouse) or 4-species (additionally including macaque comparison).

Supplementary Table 5. One-to-one orthologous genes across human, lemur, mouse.
Rows are individual genes with one-to-one orthologues across the three species, with columns showing gene type, description, gene ID, symbol, and homology type assigned by the NCBI and Ensembl orthology databases for each of the species. The table also includes columns showing the orthologues in rhesus and crab-eating macaques as assigned in the Ensembl orthology database. Genes without a macaque orthologue, or with non one-to-one mapping, were left blank in the corresponding macaque column. Note, the homology relationship is between human and indicated species, except for mouse which is between mouse and lemur. A small number of one-to-one orthologues were curated manually as indicated in column 'isManualCuration'.

Supplementary Table 6. Genes with a species conserved, primate-enriched, or primate-depleted expression for each molecular cell type across human, lemur, and mouse.
The first sheet ("conserved") lists the genes enriched in each cell type, regardless of species. Columns indicate the gene (human symbol shown), information on the respective cell type (tissue, compartment, and annotation), as well as *p*-value and fold-change comparing expression in the cell type to that of the rest of the cells in the dataset.
The second ("primate high") and third ("primate low") sheets list the genes with a primate-enriched or primate-depleted expression, respectively, for each of the cell types. Columns indicate the gene (human symbol shown), information on the respective cell type (tissue, compartment, and annotation), as well as *p*-values and fold-changes comparing both the human and lemur cells of the cell type vs. mouse cells of the orthologous cell type.

Supplementary Table 7. Global patterns of gene expression conservation across human, lemur, and mouse.
Rows are individual gene orthologues and columns show the human gene symbol, the expression similarity scores between human and lemur (r_gHL), human and mouse (r_gHM), and lemur and mouse (r_gLM), as well as three pairwise differences of these scores and their significance (*p*-value) (see Methods). Additional columns include human diseases the genes are associated with (Online Mendelian Inheritance in Man (OMIM) database[3]), and the categorization of the gene (e.g., "highly_conserved": expression conserved across human/lemur/mouse; "HL_conserved": expression across human/lemur and divergent in mouse).

Supplementary Table 8. Gene set enrichment analysis of the genes showing a highly-conserved expression pattern.
Table exported from g:Profiler with rows listing statistically enriched gene sets and columns showing information about the gene set, enrichment statistics, and related highly-conserved genes.

Supplementary Table 9. Complete blood count (CBC) and other parameters of lemurs at time of euthanasia.

	Parameter	Unit	Lemur ID			
			L1	L2	L3	L4
At euthanasia	Age	Years	9.8	10.1	11.8	11.8
	Season (Date of euthanasia)		LD (06/01/17)	LD (09/06/17)	LD (07/24/18)	LD (08/29/18)
	Number of months in LD prior to euthanasia		3	6	5	6
CBC	White blood cells	K/ul	11.09	32.07 ↑	-	1.37 ↓
	Red blood cells	M/ul	9.14	4.42 ↓	-	7.24 ↓
	Hemoglobin	gm/dL	14.0	7.4	-	11.3
	Hematocrit	%	38.1	23.2	-	37.6
	Platelet	K/ul	735	237	-	39
	Neutrophils	%	56 ↑	91 ↑	-	43 ↑
	Lymphocytes	%	34 ↓	4 ↓	-	48 ↓
	Monocytes	%	7	5	-	6
	Eosinophils	%	3	0	-	3
	Basophils	%	0	0	-	0

↑/ ↓ = higher/lower than reference value according to [4]; SD = short-day season (10:14-h light:dark); LD = long-day season (14:10-h light:dark).
Note the significant leukocytosis and neutrophil left-shift observed in L2 which likely reflects the high inflammatory state of the animal due to uterine cancer metastatic to the lung, Klebsiella pneumonia and metritis with resultant septicemia (more detail in accompanying paper[1]). The relative thrombocytopenia seen in L4 is the likely cause for the spontaneous pulmonary and renal hemorrhages found on necropsy. CBC values were not obtained for L3 given limited sample that was prioritized for chemistry panel and sequencing.

Supplementary Methods

Tissue procurement

At the time of euthanasia, blood was immediately collected from each individual via cardiocentesis for sequencing of leukocytes, biobanking, and plasma blood tests, including a complete blood count with cell differential (CBC) (Supplementary Table 9) and a complete metabolic panel (CMP). See (Casey et al., 2021)[5] for CMP values and the detailed histopathology report of the four lemurs.

Organs and tissues were then sequentially collected by a veterinary pathologist. The following delineates the sequential order of organ and tissue harvest for scRNA-seq for each individual:

Lemur #1 (male): blood, lung, frontal cortex of brain (left hemisphere)

Lemur #2 (female): blood, lung, heart and aorta, tongue, trachea, spleen, liver, diaphragm, pancreas, small intestine, colon, kidney, uterus, bladder, brain cortex (right hemisphere) and brainstem, bone and bone marrow, adipose tissue, skin, limb muscle

Lemur #3 (female): blood, heart, spleen, trachea, lung, liver, kidney, uterus, mammary gland, bone marrow. Ovaries and an abdominal mass (presumed mesenteric lymph node tumor) were also harvested for scRNA-seq, however the dissociation protocols were unsuccessful with too few viable cells to proceed with sequencing.

Lemur #4 (male): blood, thymus, heart, pancreas, liver, brain cortex (right hemisphere) and brainstem, hypothalamus and pituitary gland, lung, aorta, tongue, trachea, spleen, diaphragm, adipose tissue, small intestine, colon, kidney, testis, bladder, eye, skin, limb muscle, bone and bone marrow

Organs were processed immediately after collection by tissue experts, except L3's which were processed one to two days post-necropsy due to an unforeseen sudden decline in the animal's physical condition. L3 was found unconscious and unresponsive in her cage during routine daily monitoring, and therefore was urgently euthanized. Necropsied tissues were preserved immediately in HypoThermosol FRS solution (Biolife Solutions #101102) at 4°C overnight. However, because of an unexpected refrigerator malfunction, samples were exposed to 4-16°C for up to 10 hours before downstream processing (i.e., dissociation into single cell suspensions, followed by cDNA and library preparation using the 10x protocol). Despite this prolonged ischemic exposure, expression of immediate early genes (e.g., FOS, FOSB, JUNB, JUBD, EGR1, ZFP36) and those encoding heat shock proteins (e.g., HSPA8, HSPB1) were not significantly higher in L3. In fact, the overall transcriptomic profiles of L3 were similar to that of L1, L2, and L4, with the same cell types clustering together across all animals regardless of individual.

Histopathological image repository

Slides from all animals in this study were scanned in collaboration with the Human Pathology and Histology Service Center of the Stanford University School of Medicine Department of

Pathology. A plugin (https://github.com/czbiohub/svs-polygon-cropping) for Napari, a multi-dimensional image viewer was used to crop out candidate single images of organs and tissues from each histology slide. The slides can be viewed online at https://tabula-microcebus.ds.czbiohub.org/. See (Casey et al., 2021)[5] for the detailed histopathology report of the four lemurs. The following is a breakdown (in alphabetical order) of all organs and tissues harvested and imaged for each individual:

Lemur #1 (male): adrenal glands, cecum, esophagus, eye, gallbladder, kidney, large intestine, larynx, liver (left stifle joint), lungs, radius/ulna (right-mid) fracture, reproductive tract, salivary gland, scapulohumeral joint (right), skull/nasal cavity, small intestine, spleen, stifle joint (right), stomach, thyroid, tibia/fibula, tongue, trachea, vertebral column

Lemur #2 (female): adrenal glands, aorta, carpus, cecum, eye, heart, kidney, large intestine, larynx, liver, lung, lymph node (tracheobronchial), pancreas, scapula/elbow (sagittal), skeletal muscle, skin, skull/nasal, small intestine, spine, spleen, sternum, stifle joint (sagittal), stomach, tarsus, thymus, thyroid, tongue, trachea, urinary bladder, uterus

Lemur #3 (female): abdominal mass, adrenal glands, aorta, cecum, cervix, colon (proximal), ears, esophagus, eyes, heart, kidney, liver, lungs, nasal cavity, nasal/mandible/maxilla, ovary, rectum, ribs, salivary glands, small intestine, spine, spleen, stifle joint (right and left), stomach, thyroid, tibia (right and left), tongue, trachea, uterus, vagina

Lemur #4 (male): aorta, brain (left hemisphere), cecum, colon (proximal and distal), duodenum, esophagus, eye, heart, ileum, jejunum, kidney, larynx, liver, lumbar, lung, male reproductive tract, nose, pancreas, pericardial tissue, sacral vertebrae), skull, spine (cervical, spleen, stifle joint, stomach, thoracic, thymus, thyroid, tongue

Preparation of single cell suspensions and FACS-sorting for scRNA-seq

Lysis plates for flow sorted cells were prepared as previously described [6,7] by dispensing lysis buffer into each well (0.4 ul and 4 ul for 384-well and 96-well plates, respectively) using a Mantis liquid handler (Formulatrix). Plates were then sealed, centrifuged, flash frozen on dry ice, and stored at -80°C until used for FACS.

FACS sorting with Sony (SH800s) or Becton Dickinson (Aria III) sorters was performed on cell suspensions to isolate single cells into 384- or 96-well plates. Tissue dissociation and single cell suspension protocols are detailed below for each tissue separately. The majority of tissues were sorted into 384-well plates, except the following were sorted into 96-well plates: blood, brain, and lung for L1; hand-picked cardiomyocytes for L2; blood for L4 (the latter of which was also sorted into 384-well plates). No sorting was done for L3.

Sorters were calibrated for dispensing accuracy before cell loading and repeated after every 8-10 sorted plates. Up to 5 mL of single cell suspension was filtered, gently vortexed and then loaded onto the FACS machine. Pressure adjustments were employed to check cell concentration and suspensions diluted with FACS buffer (2% FBS in PBS) or plain PBS as needed. Single cells were selected by forward scatter, with initial gating set to exclude cell debris and platelet

aggregates. Cells were sorted using the highest purity setting to enrich for single cells. Sytox blue nucleic acid stain (ThermoFisher #S34857) was used for all samples to identify and exclude dead cells (Supplementary Fig. 5).

After FACS, plates were immediately sealed and labeled, spun down, and snap frozen on dry ice. Sorting was typically completed for each 384-well plate within 6-10 minutes, and for each 96-well plate in <5 minutes. FACS-sorting was performed only for tissues in the SS2 pipeline, and not for any samples in the 10x pipeline (see directly below).

Library preparation, quality control, and sequencing

10x Genomics. Single cells were isolated and analyzed with the 10x Genomics single cell RNA-sequencing pipeline (Chromium Single Cell 3' Library and Gel Bead v2 Chemistry kit) as previously described [6–8]. Based on the concentration of the cell suspension (determined after tissue dissociation by manual counting using a hemocytometer or by automated cell counter), samples were concentrated or diluted with 2% FBS in PBS to a target concentration of about 10^6 cells/mL. Then, cell suspensions were loaded directly into microfluidic chips (~5000 cells per sample) on a 10x Chromium Controller, which facilitated single cell capture, followed by reverse transcription to cDNA and barcoding within droplet emulsions. For cDNA amplification, 12 PCR cycles were used (Biorad C1000 Touch Thermal-cycler). Quality of the amplified cDNA was determined on a 12 or 96 capillary Fragment Analyzer using the High Sensitivity NGS Analysis Kit (Advanced Analytical). Similarly, the quality and average fragment length of cDNA libraries were quantified by capillary-based fragment analysis. After this, library concentration was determined by qPCR using the KAPA Library Quantification Kit (Roche) optimized for Illumina sequencing. Each library was normalized to 2 nM and then equal volumes of up to 16 libraries were combined together to constitute the final sequencing sample pool. A PhiX control library was spiked in at 0.2% to 1%. Library pools were sequenced on S2 flow cells of the NovaSeq 6000 System (Illumina) using 100 cycle reagent kits (Illumina, 20012862) with the following Read and Index barcode lengths: Read 1 (26 bp), Read 2 (90 bp), Index (8 bp).

Smart-seq2 (SS2). As previously performed in the Tabula Muris [6,7], the Smart-seq2 protocol [9,10] was employed for cDNA synthesis and library construction. FACS plates were thawed on ice, spun down, and then used for first strand synthesis. Master mixes were prepared with identical reagents at the same concentrations and dispensed into individual wells (0.6 ul for 384-well, 6 ul for 96-well plates) using a Mantis liquid handler (Formulatrix). The reverse transcription step was carried out at 42°C for 90 min and terminated by heating at 72°C for 5 min on a Biorad C1000 Touch Thermal-cycler. For second strand synthesis, PCR master mixes were prepared in the same manner as our prior Tabula projects. 1.5 ul and 15 ul of master mix were robotically added into each well of a 384-well and 96-well plate, respectively. DNA preamplification was performed on a Biorad thermal-cycler utilizing the following program: Step 1 (37°C for 30 min), Step 2 (95°C for 3 min), Step 3 (93°C for 20 sec, 67°C for 15 sec, 72°C for 4 min), Step 4 (72°C for 5 min). Step 3 was repeated for 21 cycles (96-well plates) or 23 cycles (384-well plates).

cDNA purification for 384-well plates was identical to our prior work [6,7]. For 96-well plates, cDNA purification was performed with AMPure XP beads (Beckman Coulter #A63881) in the following manner. 18 ul of beads (0.7x) were added to each well by multichannel pipetting.

Plates were sealed, gently vortexed, spun down, and then incubated at room temperature for 10 min. Beads were sequestered using a magnet until the supernatant was clear followed by decantation. Two rounds of washing were then performed (170 ul of 80% ethanol for each well). After removal of ethanol, plates were allowed to dry at room temperature for 5 to 15 min. Subsequently, beads were resuspended in 16 ul of EB elution buffer (Qiagen #19086), incubated for 5 min, and finally eluted.

cDNA quality was assessed by capillary electrophoresis on a Fragment Analyzer (AATI) for all 384- and 96-well plates. Candidate wells were selected for downstream processing if their cDNA concentrations were at least one standard deviation beyond the mean concentration of the blanks and the size range of their cDNA was consistent with the size spectrum of RNAs expected for a primate species (500 – 5000 bp). Samples from 96-well plates were reformatted into 384-well plates at a target concentration between 0.05 ng – 0.16 ng/ul using a Mosquito X1 single channel pipetting robot (TTP Labtech), with dilutions in EB buffer performed using a Mantis liquid handler.

SS2 sequencing libraries were prepared for the Illumina platform as described previously [6,7,11] using a starting cDNA volume of 0.4 ul. For L1 and L2, tagmentation, neutralization, and indexed PCR reactions (with dual unique i5 and i7 indexing primers) were performed using the Nextera XT Library Sample Preparation Kit (Illumina #FC-131-1096). Tagmentation was carried out at 55°C for 10 min followed by neutralization at room temperature for 5 min. Thermo-cycler settings for PCR amplification entailed: Step 1 (72°C for 3 min), Step 2 (95°C for 30 sec), Step 3 (95°C for 10 sec, 55°C for 30 sec, 72°C for 1 min; repeated 12x), Step 4 (72°C for 5 min). For L4, an in-house library preparation protocol was employed and optimized, using a different source of Tn5 enzyme (Quantitative Biosciences QB3 MacroLab core facility, UC Berkeley). No significant differences in tagmentation size, library quality, and sequencing read depth were seen between libraries made using our in-house protocol versus the Nextera kit.

Library pooling and quality control were performed as previously detailed [6,7]. After library construction, wells of each library plate were pooled initially using a Mosquito liquid handler (TTP Labtech), followed by hand pipetting into a single microcentrifuge tube (one tube per library plate). Two rounds of purification using AMPure XP beads (0.8x followed by 0.7x) were then performed involving two washes with 80% ethanol for each purification, followed by elution (100 ul elution buffer for the first round and 40 ul for the final elution). Library quality and the average fragment size were then measured by capillary electrophoresis on a Fragment Analyzer (AATI) and library concentrations subsequently quantified by qPCR (Kapa Biosystems #KK4923) on a CFX06 Touch Real-Time PCR Detection System (Biorad). Each library plate pool was normalized to 2 nM (L1 and L2) or 4 nM (L4) and equal volumes for up to 20 plates were mixed together to make the final sequencing sample pool. A PhiX control library was spiked in at 0.2% prior to sequencing.

Libraries were sequenced on a NovaSeq 6000 System (Illumina) using 2 x 100 bp paired-end reads and 2 x 12 bp dual unique index reads with 200 or 300 cycle kits designed for either S2 or S4 flow cell configurations (Illumina, 20012861, 20012860, 20027466, 20012866).

Genome alignment

Individual chromosome scaffolds of the *Microcebus murinus* genome assembly (Mmur 3.0, Refseq assembly accession: GCF_000165445.2 available from NCBI https://www.ncbi.nlm.nih.gov/assembly/GCF_000165445.2/) were concatenated together in Linux to create a whole genome FASTA file. The gene annotation file was retrieved from the NCBI Microcebus murinus Annotation Release 101 FTP site. For tRNA genes, we summed all identically named tRNA gene loci (e.g., 'TRNAA-AGC-1', 'TRNAA-AGC-2', etc. into one 'gene', 'TRNAA-AGC'). This resulted in a total of 31,509 genes.

10x alignment as described in Main Methods. For SS2 alignment, the following options were used for STAR mapping: --alignIntronMin 20, --alignIntronMax 1000000, --alignMatesGapMax 1000000, --alignSJoverhangMin 8, --alignSJDBoverhangMin 1, --outBAMcompression 10, --outFilterMultimapNmax 20, --outFilterMismatchNmax 999, --outFilterMismatchNoverReadLmax 0.04, --outFilterType BySJout, --outSAMattributes NH HI AS NM MD, --outSAMstrandField intronMotif, --outSAMtype BAM SortedByCoordinate, --outSAMunmapped Within, --outSJfilterReads Unique, --quantMode TranscriptomeSAM GeneCounts, --quantTranscriptomeBAMcompression 10, --sjdbOverhang 99, --sjdbScore 1

SS2 reads were first pre-processed with skewer (version 0.2.2), an adapter trimmer optimized for Illumina paired-end reads. Then, STAR alignment was performed, with first pass mapping to identify splice junctions. These junctions were subsequently incorporated to generate a new genome, after which second pass mapping was initiated. A remapped BAM file with second pass mapping statistics was created using samtools. FPKM was calculated with cuffquant, and gene expression estimated using RSEM (version 1.3.1). Read quality was then assessed with HTSEQ, and the count tool used to sum up for each gene the total number of aligned reads overlapping its exons, generating a gene counts table.

Cell filtering

For all tissues except the heart the final cutoff was 500 genes, 1000 UMI (10X), 5000 reads (SS2). For the heart the cutoff was 50 genes, 100 UMI (10X), and 5000 reads (SS2).

FIRM integration

The FIRM algorithm can integrate multiple scRNA-seq datasets by iteratively integrating two datasets together. The tissue-level integrated datasets (https://tabula-microcebus.ds.czbiohub.org/organs) were constructed by first integrating the tissue datasets of the same individual that were sequenced by different methods (10x or SS2), and then further integrating datasets from each lemur individual. This created a single object for each tissue that combined scRNA-seq data from each individual and sequencing method.

The atlas-wide integrated dataset was created by first integrating datasets across individuals, then tissues, and lastly sequencing methods. Typically, tissues with more overlapped highly variable genes were integrated first. Because testes cells (predominantly germ cells) showed the most distinctive transcriptional profiles compared with cells of the other tissues, the testes dataset was

first separately integrated across different sequencing methods and then integrated with the rest of the tissues.

When applying FIRM, the top 4000 highly variable genes for each of the two datasets to be intergrated were identified using the "FindVariableFeatures" function in Seurat. FIRM then used the intersection, union, or the top ranked genes of the detected highly variable genes of the two datasets for principal component analysis (method chosen according to best performance). For dimensionality reduction, the number of principal components was chosen as the larger number of principal components used for dimensionality reduction of the two pre-integration datasets.

Comparison of expression profiles among lemur cell types: calculating differentially expressed genes (Fig. 4b-d; Extended Data Fig. 5j)

The Wilcoxon rank-sum test was used to identify differentially expressed genes between groups of cell types. To search for genes that were preferentially expressed in the sperm/sperm progenitor cells and immune progenitor/proliferating cells in comparison to cells of other compartments (epithelial, endothelial, stromal), we first applied a right-tailed hypothesis rank-sum test to genes that were expressed at a moderate level (>0.3 in relative expression level, natural log transformed and normalized to the cell type maximal expression level across the atlas) in at least one cell type in the sperm/sperm progenitor cells and in immune progenitors. These genes were then tested to remove the genes that were not differentially expressed between sperm/sperm progenitor cells versus immune progenitor/proliferating cells. The Bonferroni method was used for multiple testing correction of all p-values. Genes that passed all criteria in the top 1% of p-values were manually examined to confirm consistency of expression. The same workflow was performed to search for genes preferentially expressed in spermatogonia and immune progenitor/precursor cells in comparison to proliferating cells of other compartments (i.e., epithelial, stromal, neural), in Schwann cells and stromal cells in comparison to cells of the neural compartment, as well as in glial cells of the central nervous system in comparison to Schwann cells and stromal cells.

Tissue Dissociation Protocols

Nervous System

Brain cortex, brainstem, hypothalamus, pituitary gland

After removal from the skull, cerebral tissue was placed directly in ice cold Hibernate A medium (Gibco #A1247501). The relevant brain regions were microdissected for the following animals: L1, left frontal cortex; L2, entire right cortex and entire right brainstem; and L4, entire right cortex and entire right brainstem with the hypothalamus and pituitary gland dissected apart from the brain. Each region was mechanically minced into ~1 mm^3 pieces with a scalpel, and then digested under gentle agitation in Hibernate A minus Calcium (BrainBits #HACA) containing 20 U/mL Papain (Worthington #LS003126) at 34°C for 1 hour (except for the pituitary gland which was dissociated for 10 min). The digested homogenate was subsequently washed with 10% FBS and 10 mM HEPES in cold L-15 medium (Gibco #11415), and triturated 10 times using a P1000 pipette, and then 10 more times with a 500 µm sterile glass pipette. Next, the cell suspension was

overlaid on the top surface of a Percoll solution containing 1 mL of Percoll (Sigma #P1644) and 4 mL of L-15 and 10 mM HEPES. The gradient was centrifuged at 400g, 4°C for 9 minutes and the solution decanted except for a 1 mL layer at the bottom. This layer was spun down at 400g, 4°C for 4 min, and the resulting cell pellet resuspended in buffer consisting of phenol-red free L-15 (Gibco #21083027), 10 mM HEPES, 0.5% BSA, and 0.01% DNase (Sigma #DN25). The cell suspension was filtered through a sterile 100 µm strainer (Falcon #352360). Concentration was determined by manual cell counting on a hemocytometer and adjusted to 10^6 cells/mL. A portion of the sample was aliquoted for 10x processing with the remaining cells stained with Sytox blue (ThermoFisher #S34857) prior to FACS-sorting for SS2 processing. Due to a shortage of reagent during tissue processing of L4, Hibernate A minus Calcium was used in place of L-15 for all the protocol steps detailed above.

Eye retina

Extracted eye tissue was placed in cold Dulbecco's Modified Eagle Medium (DMEM, Gibco #11054020). The sclera, choroid, and retinal pigment epithelial (RPE) layers were separated from the retina manually and mechanically using microsurgical instruments. The retinal sample was further dissociated in 0.25% trypsin-EDTA (Gibco #12605010) at 37 °C for 30 minutes, and detached cells collected every 5 mins in cold DMEM containing 10% FBS. Cell suspensions were resuspended in PBS containing 10% FBS, then filtered through a 100 µm nylon cell strainer (Falcon #352360). Concentration was determined by manual cell counting on a hemocytometer and adjusted to 10^6 cells/mL. A portion of the sample was aliquoted for 10x processing with the remaining cells stained with Sytox blue (ThermoFisher #S34857) prior to FACS-sorting for SS2 processing. Dissection of RPE cells was performed as previously described [12], however the sample was not sequenced due to low viable cell count.

Integumentary System

Skin

A similar dissociation protocol was employed in the Tabula Muris [6,7]. After extracting the skin, the underlying subcutaneous fat was removed with a scalpel. The remaining skin layer was then incubated with gentle agitation in digestion buffer consisting of 0.25% trypsin (Thermo #15050057) and 15% FBS in DMEM (Gibco #11054020) at 37°C for 30 min. The top layer of epidermal cells was scraped away from the dermis, releasing keratinocytes in a single cell suspension. After an additional digestion in trypsin for 5 minutes, cells were filtered in succession through 70 µm and 40 µm cell strainers (Falcon #352350, #352340, respectively) on ice, then washed with cold 5% FBS in PBS. Cells were spun down at 500g, 4°C for 5 min and the final pellet resuspended in cold 5% FBS in PBS. Concentration was determined by manual cell counting on a hemocytometer and adjusted to 10^6 cells/mL. A portion of the sample was aliquoted for 10x processing with the remaining cells stained with Sytox blue (ThermoFisher #S34857) prior to FACS-sorting for SS2 processing.

Fat

A similar dissociation protocol was employed in the Tabula Muris [6,7], aimed at extracting the stromal vascular fraction (SVF) from adipose tissue. After extraction, fat tissues including inguinal subcutaneous (SCAT, L2 and L4), perigonadal (GAT, L2 only), mesenteric (MAT, L2 and L4), and interscapular brown (BAT, L2 and L4) adipose tissues were individually dissected

out and minced using fine surgical instruments in a petri dish on ice until forming a slurry. Tissues were then digested under continuous gentle shaking at 37°C for 30 minutes in Ham's F-10 medium (Gibco #11550043) containing 760 U/mL collagenase II (Worthington #LS004177) and 1 U/mL Dispase II (Gibco #17105-041). After trituration, cells were filtered sequentially through 100 µm (Falcon #352360) and 40 µm (Falcon #352340) strainers on ice and then washed with cold Ham's F-10 medium containing 10% horse serum (Invitrogen #16050114). Concentration was determined by manual cell counting on a hemocytometer and adjusted to 10^6 cells/mL for 10x processing. Note that the perigonadal fat was collected from L2 (female) and surrounding tissue was likely sampled (including from the ovary), given that granulosa cells and mesothelial cells were found in the scRNA-seq dataset. L4's fat samples were combined for 10x sequencing due to limited space on the microfluidic chips.

Mammary gland
L3's mammary gland tissue was placed at 4°C in HypoThermosol FRS solution (Biolife solutions #101102) for 1 day. Subsequently, the gland tissue was placed in 10 mL of cold DMEM/12 medium (Gibco #12634010) and directly minced with a sterile razor blade into approximately 1 mm fractions. The samples were then incubated under gentle shaking in digestion buffer (Stem Cell Technology #07912) consisting of 150 U/mL collagenase and 50 U/mL hyaluronidase in DMEM at 37°C for 2 hrs, interspersed by mild pipetting every 30 minutes to promote further tissue dissociation. The digested homogenate was subsequently spun down at 1500 rpm, followed by treatment with 5 mL of ACK lysing buffer (Lonza #10-548E) on ice for 5 minutes to remove red blood cells. Two additional digestion steps were then performed in succession, first with 5 mL of 0.25% trypsin-EDTA (Invitrogen #252001144) for up to 5 min, followed by a shorter digestion with DNase I (Worthington #LS002139). Dissociated cells were then filtered through a sterile 40 µm strainer (Falcon #352340) and washed with buffer containing 1% BSA and 2% FBS in HBSS. Concentration was determined by manual cell counting on a hemocytometer and adjusted to 10^6 cells/mL for 10x processing.

Musculoskeletal System

Limb muscle & Diaphragm
Muscle was extracted from the hind limb below the knee. Diaphragm was isolated in one piece by cutting along the inner side of the ribcage. After extraction, each sample was washed in cold wash media containing Ham's F-10 (HyClone #SH30025.01), 10% horse serum (Invitrogen #16050114), and 1% pen/strep (Omega Scientific #PS-20), and then gently dried with Kimwipes. The samples were further mechanically dissociated with surgical scissors, and subsequently digested under gentle continuous shaking at 37°C for 35 minutes in dissociation buffer (0.2% (w/v) collagenase II (Worthington #LS004177) in wash media). Cells were spun down at 4°C, 1600g for 5 minutes, the supernatant decanted and the pellet further digested in PBS containing 1% Dispase (Gibco #17105-041) and 0.5% collagenase II. After vortexing, the samples were incubated at 37°C for 20 min under gentle continuous shaking. Post-digestion, cell suspensions were pulled five times through a 10 mL syringe (BD #309604) using a 20-gauge needle (Fisher #305175), and then filtered through a 40 µm nylon cell strainer (Falcon #352340). The samples were washed and spun down for 5 min at 1600g, decanted, and resuspended in wash media and filtered in 5 mL filter-cap tubes (Falcon #352235). Concentration was determined by manual cell counting on a hemocytometer and adjusted to 10^6 cells/mL. A portion of the sample was

aliquoted for 10x processing with the remaining cells stained with Sytox blue (ThermoFisher #S34857) prior to FACS-sorting for SS2 processing. Some cells were also stained with anti-human antibodies, NCAM1/CD56 (Biolegend #304628), THY1/CD90 (BioLegend #328114), ITGB1/CD29 (Biolegend #303007), and Sytox blue, and immediately FACS-sorted for functional experiments (see Kang et al.[13]).

Bone
Bones (humerus and spine) were first processed to collect bone marrow (see below). The unfiltered residual bone tissue was further minced with a razor blade and resuspended in digestion buffer supplemented with 3000 U/mL collagenase II (Sigma-Aldrich #C6885) and 100 U/mL DNase I (Worthington #NC9199796). The sample was incubated at 37°C for 1 hour under gentle agitation, then filtered through a 70 µm nylon mesh strainer (Falcon #352350) and the digestion reaction quenched with 2% FBS in PBS (Gibco #C14190500BT). The skeletal cells were separated from red blood cells and bone dust by density gradient separation using 1:1 of room temperature Ficoll Histopaque 1.119 g/mL density gradient media (Sigma-Aldrich #11191). The buffy coat was collected, washed with 2% FBS in PBS. Concentration was determined by manual cell counting on a hemocytometer and adjusted to 10^6 cells/mL. A portion of the sample was aliquoted for 10x processing with the remaining cells stained with Sytox blue (ThermoFisher #S34857) prior to FACS-sorting for SS2 processing.

Circulatory System

Bone marrow
After bone harvesting (humerus and spine), a paper towel was used to remove any residual fibers and muscle. A mortar and pestle were used to crush the bone and extract the marrow. Several mLs of 2% FBS in PBS containing 1:100 DNase I (Worthington #LS006343) were added to the mortar to keep cells alive and prevent clumping of cells. Crushing of bone continued until no further red fluid leaked from the dissociated tissue. The sample was then filtered through a 40 µm strainer (Falcon #352340) into a 50 mL Falcon conical tube containing 2% FBS in PBS and DNA I placed on ice. The unfiltered residue was collected and processed for bone cells (see above). The filtrate was spun down at 4°C, 300g for 5 minutes, and resuspended in 2% FBS in PBS and filtered through a 70 µm strainer (Fisherbrand #22363548). Concentration was determined by manual cell counting on a hemocytometer and adjusted to 10^6 cells/mL. A portion of the sample was aliquoted for 10x processing with the remaining cells stained with Sytox blue (ThermoFisher #S34857) prior to FACS-sorting for SS2 processing. Note, L3's bone marrow was processed on the day of the euthanasia using protocol above, but the single cell solution was suspended in Bambanker solution (Fisher Scientific #NC9582225) and frozen at -80°C using a cell freezing container for adequate cryopreservation. On the day of 10x processing, cells were thawed in 37°C water bath and then resuspended in 2% FBS in PBS solution at the appropriate cell concentration.

Blood
After cardiocentesis of the left ventricle, blood was gently aspirated through a fine surgical needle and collected in EDTA-coated tubes. The sample was diluted 1:10 with 2% FBS in PBS, and additional DNase I (Worthington #LS006343) supplemented if blood started to coagulate. Diluted room temperature blood was then gently layered, without mixing, into a 15 mL sterile

Falcon conical tube containing half of the diluted sample volume of room temperature Ficoll Histopaque 1.119 g/mL density gradient media (Sigma-Aldrich #11191), forming 1 part Ficoll solution at the bottom and 2 parts sample at the top. Using the higher density Ficoll mix compared to the standard Ficoll density (1.077 g/ml) allows for polymorphonuclear cells to be separated, in addition to mononuclear cells. The sample was spun down at room temperature, 400g for 30 minutes with the brake setting off. The leukocyte-rich buffy coat was then transferred with use of a pipette to a new 15 mL Falcon tube. Cold 2% FBS in PBS was added to fill up the tube and the solution was mixed by inverting. The sample was then spun down at 4°C, 500g for 5 minutes (prior to this, all steps had been carried out at room temperature). After removing the supernatant, cells were resuspended in 1 mL cold 2% FBS in PBS. The single cell solution was gently pipetted up and down to further dissociate any cell clumps. If cells persisted to clump, 0.1 mg/mL of DNase I was added to the sample, incubated on ice for 10 min, and then washed (spun down at 4°C, 500g for 5 minutes). If the sample remained red/pink colored after Ficoll (indicating presence of red blood cells), it was diluted 1:10 with 1X BD Pharm Lyse, a red blood cell lysing buffer (BD Biosciences #555899), incubated on ice for 5-10 min until the cloudy solution turned transparent red, and then washed (spun down at 4°C, 500g for 5 minutes). Cells were filtered through a 70 µm strainer (Fisherbrand #22363548). Concentration was determined by manual cell counting on a hemocytometer and adjusted to 10^6 cells/mL. A portion of the sample was aliquoted for 10x processing with the remaining cells stained with Sytox blue (ThermoFisher #S34857) prior to FACS-sorting for SS2 processing. Note, L3's blood was processed on the day of the euthanasia using protocol above, but the single cell solution was suspended in Bambanker solution (Fisher Scientific #NC9582225) and frozen at -80°C using a cell freezing container for adequate cryopreservation. On the day of 10x processing, cells were thawed in 37°C water bath and then resuspended in 2% FBS in PBS solution at the appropriate cell concentration. L4's blood clotted after collection, and so the red blood cell lysis was done prior to the Ficoll separation, in addition to use of DNase I, in order to dissociate the clot as much as possible.

Spleen
After extraction, splenic tissue was washed in 1x HBSS (ThermoFisher #14175), minced with razor blades, and further dissociated mechanically using the flat-end of a plunger. The sample was then triturated sequentially through 100 µM, 70 µM, and 40 µM strainers (Falcon #352360, #352350, #352340, respectively). Cells were washed in 2% FBS in PBS followed by ACK treatment (Gibco #A10492-01) at room temperature for 5 minutes. Residual debris was removed using debris removal solution (Miltenyi Biotec #130-109-398) per factory instructions. Cells were resuspended in 2% FBS in PBS. Concentration was determined by manual cell counting on a hemocytometer and adjusted to 10^6 cells/mL. A portion of the sample was aliquoted for 10x processing with the remaining cells stained with Sytox blue (ThermoFisher #S34857) prior to FACS-sorting for SS2 processing. Note, L3's extracted splenic tissue was first preserved at 4°C for 2 days in HypoThermosol FRS solution (Biolife Solutions #101102) before continuing on with the above delineated steps.

Thymus
Thymic tissue was crushed with a 70 µm strainer (Falcon #352350) and centrifuged at 5°C, 270g for 5 minutes. The cell pellet was digested with 2.2 mg/mL collagenase II (Sigma #C6885) at 37°C for 10 min, then incubated under gentle agitation at 37°C for 30 more minutes. Digestion

was quenched with FACS buffer containing 2% FBS, 1% antibiotics (Gibco #15240-062), and 10% pluronics (ThermoFisher #24040032) in PBS. The cell suspension was pelleted at 5°C, 270g for 5 min, then washed, resuspended in FACS buffer, and stained with Sytox blue (ThermoFisher #S34857) prior to FACS-sorting for SS2 processing.

Heart
After extraction, cardiac tissue was placed on ice in a sterile 60-mm polystyrene culture dish (Thermofisher #174888) containing perfusion buffer (135 mM NaCl, 4 mM KCl, 1 mM $MgCl_2$, 10 mM HEPES, 0.33 mM NaH_2PO_4, 10 mM glucose, 10 mM 2,3-butanedione monoxime, 5 mM taurine; overall pH 7.2) to inhibit myosin activity and induce cardioplegia. The 4 cardiac chambers were further dissected apart from each other (left atrium, LA; left ventricle LV, right atrium, RA; right ventricle, RV) using fine surgical forceps and scissors. Each chamber fraction was then moved to individual 15 mL sterile Falcon conical tubes containing digestion buffer (0.3 mg/g body weight collagenase D, Roche #11088858001; 0.4 mg/g body weight collagenase B, Roche #11088807001; 0.05 mg/g body weight Protease type XIV, Sigma #P5147; in sterile water), and incubated with continuous gentle shaking at 37°C for 10-20 minutes. During this period, samples were gently pipetted up and down two times to facilitate dissociation. After trituration of the majority of tissue into a single cell suspension, the sample was filtered through a 70 µm nylon mesh strainer (Falcon #08-771-1). The concentration of the eluent (non-myocyte fraction) was determined by manual cell counting on a hemocytometer and adjusted to 10^6, and subsequently divided into 2 aliquots: one for the 10x processing and the second stained with Sytox blue (ThermoFisher #S34857) prior to FACS-sorting for SS2 processing. The fraction caught by the mesh strainer (cardiomyocyte fraction) was handled similarly, but divided into 3 aliquots (10x, SS2, and from the remaining fraction cardiomyocyte cells were hand-picked under light microscopy with a 20 µl pipette and deposited cell by cell into a hard-shell 96-well plate (BioRad #HSP9601) for further SS2 processing (without FACS-sorting). Note, L3's extracted cardiac tissue was first preserved at 4°C for 1 day in HypoThermosol FRS solution (Biolife Solutions #101102) before continuing on with the above delineated steps.

Aorta
After extraction, aortic tissue was minced using sterile microsurgery tools, then digested with 2.2 mg/mL collagenase II (Sigma #C6885) at 37°C for 10 min, and incubated under gentle agitation at 37°C for an additional 30 minutes. The digestion reaction was quenched with FACS buffer containing 2% FBS, 1% antibiotics (Gibco #15240-062), and 10% pluronics (ThermoFisher #24040032) in PBS. Cells were pelleted at 5°C, 270g for 5 min, and resuspended and washed in FACS buffer. Concentration was determined by manual cell counting on a hemocytometer and adjusted to 10^6 cells/mL for 10x processing.

Respiratory System

Lung
Tissue samples were processed into single cell suspensions in a manner similar to the protocol used in Tabula Muris [6,7]. After extraction of both right and left lungs from the thoracic cavity, the tissue was triturated using sterile microsurgery tools and placed in gentleMACS c-tubes (Miltenyi #130-096-334) with digestion buffer consisting of 400 µg/mL Liberase DL (Sigma #5466202001) in RPMI (Gibco #72400120). A partial dissociation was performed on a

gentleMACS Dissociator (Miltenyi #130-093-235) with the ‘m_lung_01’ program. The sample was then incubated at 37°C on a nutator set to gentle agitation for 30 minutes, followed by a final complete dissociation step on the gentleMACS using the ‘m_lung_02’ program. The resulting homogenate was washed with cold 5% FBS in PBS, spun down at 300g, 4°C for 5 minutes, resuspended in cold 5% FBS in PBS, and subsequently filtered through a 70 μm strainer (Fisherbrand #22363548). The suspension was centrifuged and resuspended in FACS buffer (2% FBS in PBS). Concentration was determined by manual cell counting on a hemocytometer and adjusted to 10^6 cells/mL. A portion of the sample was aliquoted for 10x processing with the remaining cells stained with Sytox blue (ThermoFisher #S34857) prior to FACS-sorting for SS2 processing. Note, L3’s extracted lung tissue was first preserved at 4°C for 1 day in HypoThermosol FRS solution (Biolife Solutions #101102) before continuing on with the above delineated steps.

Trachea

The dissected trachea was rinsed with cold PBS. The tissue was then cut longitudinally with a sterile scalpel to expose the epithelium and placed in 2 mL of digestion buffer containing 5 U/mL dispase (Gibco #17105041), 40 U/mL collagenase I (Gibco #17018029), 2% FBS, and 1x penicillin-streptomycin (Gibco #15140-122) in HBSS (Gibco #14175095). Tissue was minced with sterile dissecting tools in the digestion buffer and incubated at 37°C for 60 minutes, with gentle pipetting performed at regular intervals to assist dissociation. The resulting cell suspension was filtered through a 40 μm strainer (BD #08-771-1), pelleted at 21°C, 2000g for 5 mins and then washed twice with cold PBS. Subsequently, the pellet was resuspended in ACK lysis buffer at 21°C for 1 min and spun down at 21°C, 2000g for 5 minutes. Following two more wash steps in cold PBS with pelleting at 4°C, 2000g for 5 mins, the final pellet was resuspended in FACS buffer containing 2% FBS and 1x penicillin-streptomycin in cold PBS. Cells were filtered again through a 40 μm strainer. Concentration was determined by manual cell counting on a hemocytometer and adjusted to 10^6 cells/mL for 10x processing. Note, L3’s extracted tracheal tissue was first preserved at 4°C for 1 day in HypoThermosol FRS solution (Biolife Solutions #101102) before continuing on with the above delineated steps.

Gastrointestinal System

Tongue

The dissociation protocol used for L2 was the same as that in Tabula Muris [6,7]. The protocol was then adjusted for L4 to also capture mesenchymal tissues underneath the epithelium (stroma, muscle, and vessels were omitted in the Tabula Muris). After extraction, tongue tissue from L4 was immediately placed on ice. The tongue was further dissected to separate muscles and arteries. The remaining epithelium and underlying mesenchyme were minced with a razor blade, then digested at 37°C on an orbital shaker for 60 minutes in base media, M199-HEPES (ThermoFisher #12340030) supplemented with 2500 U/mL collagenase type IV (Worthington #LS004188) and 100 U/mL DNase I (Worthington #LS006343). A total of two sequential digestion steps were performed (using fresh digestion media each time). Cells were then filtered through a 70 μm strainer (Falcon #352340), pelleted (500g, 5°C, 5 min), and resuspended in PBS containing 1x penicillin-streptomycin (ThermoFisher #15140122), 1x pluronic F-68 (ThermoFisher #24040032), and 2% FBS (Atlanta Biologicals #S11550H). Concentration was determined by manual cell counting on a hemocytometer and adjusted to 10^6 cells/mL. A

portion of the sample was aliquoted for 10x processing with the remaining cells stained with Sytox blue (ThermoFisher #S34857) prior to FACS-sorting for SS2 processing.

Small intestine
After extraction, small bowel tissue was washed with cold PBS in a 50 mL sterile Falcon conical tube, then placed on a petri dish and minced with a razor blade. The sample was subsequently incubated at 37°C for 120 minutes in digestion buffer containing the following: 200 U/mL collagenase III (Worthington #LS004183), 100 U/mL DNase I (Worthington #LS002139), 1x antibiotic-antimycotic (ThermoFisher #15240062) dissolved in advanced DMEM/F12 (ThermoFisher #12634028) with 10 mM HEPES (ThermoFisher #15630080) and 1 mM sodium pyruvate (Lonza Switzerland #BW13-115E). Pipetting was performed every 15 min to facilitate complete dissociation. Cells were spun down at 1500 rpm, 4°C, for 5 min, decanted, and resuspended in FACS buffer (2% FBS in PBS) containing 100 U/mL DNase I. The sample was filtered through a 70 µm strainer (Falcon #352350). Concentration was determined by manual cell counting on a hemocytometer and adjusted to 10^6 cells/mL. A portion of the sample was aliquoted for 10x processing with the remaining cells stained with Sytox blue (ThermoFisher #S34857) prior to FACS-sorting for SS2 processing.

Colon
After extraction, colonic tissue was washed sequentially in PBS, then in PBS containing 5 mM EDTA. The tissue was then placed in fresh 5 mM EDTA/PBS and incubated at 37°C for 5-30 minutes with intermittent shaking (until complete dissociation of the crypt regions). The sample was spun down at 4°C, 1000 rpm for 2 min, decanted, and resuspended in serum-free media consisting of: advanced DMEM/F12 (ThermoFisher #12634028) with 1x antibiotic-antimycotic (ThermoFisher #15240062), 10 mM HEPES (ThermoFisher #15630080), and 1 mM sodium pyruvate (Lonza Switzerland #BW13-115E). This was followed by incubation at 37°C for 60 min, with pipetting performed every 15 minutes to ensure complete dissociation. Subsequently, the sample was spun down at 4°C, 1500 rpm for 5 min, decanted, and resuspended in digestion solution: 2% FBS in PBS with 100 U/mL DNase I (Worthington #LS002139). The sample was filtered through a 70 µm strainer (Falcon #352350). Concentration was determined by manual cell counting on a hemocytometer and adjusted to 10^6 cells/mL. A portion of the sample was aliquoted for 10x processing with the remaining cells stained with Sytox blue (ThermoFisher #S34857) prior to FACS-sorting for SS2 processing.

Liver
The dissected liver (~3 cm^3) was immediately placed on ice in a sterile 50 mL Falcon conical tube containing cold PBS. Tissue was transferred to a sterile 10 cm polystyrene culture dish (ThermoFisher #150350) containing perfusion buffer (Life Technology #17701-038) prewarmed to 37°C. Two 27G olive-tip regular bevel needles (BD #305109) were inserted into the tissue and a peristaltic pump (Watson-Marlow 120U) used to perfuse the liver for 15 minutes at 7 ml/min. The liver was then moved to a new 10 cm dish containing liver digestion media (Life Technology #17701-034) prewarmed to 37°C, and further perfused with this prewarmed media for 20 minutes at 10 ml/min. The digested media was collected in conical tubes and the softened liver tissue subsequently placed within the media, followed by incubation at 37°C for 20 min with gentle pipetting every 5 min to enhance dissociation. After additional trituration, the single cell suspension was filtered through a 100 µm nylon mesh strainer (BD #352360). The eluent

was centrifuged for 5 min, 500g, and resuspended in 2% FBS in PBS. Concentration was determined by manual cell counting on a hemocytometer and adjusted to 10^6 cells/mL. A portion of the sample was aliquoted for 10x processing with the remaining cells stained with Sytox blue (ThermoFisher #S34857) prior to FACS-sorting for SS2 processing. Note, L3's extracted liver tissue was first preserved at 4°C for 1 day in HypoThermosol FRS solution (Biolife Solutions #101102) before continuing on with the above delineated steps.

Pancreas
Following necropsy, pancreatic tissue was placed in digestion buffer consisting of 2 mg/mL collagenase VIII (Sigma #C2139) and 0.2 mg/mL trypsin inhibitor (Sigma #T6522) in PBS. Tissue was inflated within the digestion solution using a 27g needle and then incubated in a shaker at 37°C for 8-15 minutes. Following collapse of the tissue, 5 mL of stock FACS buffer (2% FBS in PBS) was added to inactivate collagenase and the cells spun down (all centrifugation steps performed for 5 min at 300 g, 4°C). The cell pellet was resuspended in FACS buffer and filtered through a 100 µm cell strainer (BD #352360). Filtered cells were washed 1x with FACS buffer and subsequently resuspended in 3-5 mL 1x red blood cell lysis buffer (Ebioscience #00-4300) to remove erythrocytes and incubated for 8-10 min at RT. After spinning down, cells were resuspended and washed twice using FACS buffer. After the final wash, cells were resuspended in FACS buffer containing 10 µg/mL DNase I (Roche #4716728001). Concentration was determined by manual cell counting on a hemocytometer and adjusted to 10^6 cells/mL. A portion of the sample was aliquoted for 10x processing with the remaining cells stained with Sytox blue (ThermoFisher #S34857) prior to FACS-sorting for SS2 processing. Note that the pancreas is surrounded by lymph nodes that are challenging to discriminate during dissection. Given that L4's 10x and SS2 pancreas datasets contain a relatively large proportion of immune cells, it is possible that a neighboring lymph node was sampled and processed with the pancreatic tissue.

Urinary System

Kidney
After extraction, renal tissue was first mechanically homogenized using a gentleMACS dissociator (Miltenyi #130-093-235) and then digested under gentle agitation at 37°C for 30 minutes in RPMI buffer containing 10 U liberase TM enzyme (Roche #5401119001), 2% FBS, and 1x antibiotic-antimycotic (Gibco #15240-062). Following trituration with a 5 mL serological pipette, cells were filtered sequentially through 100 µm, 70 µm, and 40 µm strainers (Falcon #352340 #352350 #352360, respectively) with a syringe plunger. Cells were spun down at 4°C, 400g for 10 minutes, then treated with ACK (Gibco #A10492-01) for 5 min at 21°C, and washed with 2% FBS and 1x antibiotic-antimycotic in RPMI. The final pellet was resuspended in 2% FBS in PBS and filtered into 35 µm polystyrene tubes (Falcon #352235). Concentration was determined by manual cell counting on a hemocytometer and adjusted to 10^6 cells/mL. A portion of the sample was aliquoted for 10x processing with the remaining cells stained with Sytox blue (ThermoFisher #S34857) prior to FACS-sorting for SS2 processing. Note, the protocol above was used for L2 and L4. L3's extracted kidney tissue was first preserved at 4°C for 1 day in HypoThermosol FRS solution (Biolife Solutions #101102). The dissociation protocol used for L3 was the same as that used in Tabula Muris [6,7].

Bladder

Bladder tissue was placed directly on ice after extraction, then minced. The sample was digested at 37°C on an orbital shaker for 1 hour in Medium 199 (Gibco #11150059) containing 1875 U/mL collagenase type IV (Worthington #LS004188) and 25 U/mL DNase I (Worthington #LS006343), followed by another digestion for 30 minutes in 1x trypLE (ThermoFisher #A1217701) with 25 U/mL DNase I. Cells were subsequently filtered through a 40 µm strainer (Falcon #352340), pelleted (500g, 4°C, 5 min), and resuspended in FACS buffer in PBS pH 7.4 (ThermoFisher #100100-23) containing 1x penicillin-streptomycin (ThermoFisher #15140122), 1x pluronic F-68 (ThermoFisher #24040032), and 2% FBS (Atlanta Biologicals #S11550H). Concentration was determined by manual cell counting on a hemocytometer and adjusted to 10^6 cells/mL. A portion of the sample was aliquoted for 10x processing with the remaining cells stained with Sytox blue (ThermoFisher #S34857) prior to FACS-sorting for SS2 processing.

Reproductive System

Uterus

L3's uterine tissue was placed at 4°C in HypoThermosol FRS solution (Biolife solutions #101102) for 2 days. Subsequently, tissue was washed in 1x HBSS (ThermoFisher #14175) and then minced with razor blades. The sample was digested at 37°C for 20 min in 10 U/mL liberase TM (Sigma #5401119001) in RPMI media (Thermofisher #11875119). The sample was then triturated sequentially through 100 µM, 70 µM, and 40 µM strainers (Falcon #352360, #352350, #352340, respectively). Cells were washed, spun down, and resuspended in 2% FBS in PBS. Concentration was determined by manual cell counting on a hemocytometer and adjusted to 10^6 cells/mL for 10x processing.

Testes

Immediately after extraction, the testes were detunicated, gently dissociated in PBS, and then incubated at 32°C for 10 min in PBS containing 1 mg/mL type I collagenase (Worthington, #LS004196). Cells were then pelleted via centrifugation for 5 minutes at 250g and the supernatant removed. Another round of digestion with collagenase I was employed, followed by further digestion in trypLE express enzyme (ThermoFisher #12605010) at 32°C for 15 min. During these digestion steps, the seminiferous tubules were mechanically fragmented by vigorous pipetting every 5 minutes. The sample was filtered sequentially through 70 µm and 40 µm strainers (Falcon #352350, #352340, respectively), and then resuspended in cold FACS buffer (2% FBS, 1mM EDTA in PBS). Concentration was determined by manual cell counting on a hemocytometer and adjusted to 10^6 cells/mL. A portion of the sample was aliquoted for 10x processing with the remaining cells stained with Sytox blue (ThermoFisher #S34857) prior to FACS-sorting for SS2 processing.

Supplementary References

1. Ezran, C., Liu, S. & Chang, S. Mouse lemur cell atlas informs primate genes, physiology, and disease.
2. Liu, S. et al. An organism-wide atlas of hormonal signaling based on the mouse lemur single-cell transcriptome. Nat. Commun. 15, 2188 (2024).
3. McKusick-Nathans Institute of Genetic Medicine, Johns Hopkins University (Baltimore, MD). Online Mendelian Inheritance in Man, OMIM®. https://omim.org/.
4. Der Goukassian, P. Hematology of the lesser mouse lemur (Microcebus murinus). A preliminary study. Folia Primatol. 41, 129–136 (1983).
5. Casey, K. M., Karanewsky, C. J., Pendleton, J. L., Krasnow, M. R. & Albertelli, M. A. Fibrous Osteodystrophy, Chronic Renal Disease, and Uterine Adenocarcinoma in Aged Gray Mouse Lemurs (Microcebus murinus). Comp. Med. 71, 256–266 (2021).
6. Tabula Muris Consortium et al. Single-cell transcriptomics of 20 mouse organs creates a Tabula Muris. Nature 562, 367–372 (2018).
7. Tabula Muris Consortium. A single-cell transcriptomic atlas characterizes ageing tissues in the mouse. Nature 583, 590–595 (2020).
8. Travaglini, K. J. et al. A molecular cell atlas of the human lung from single-cell RNA sequencing. Nature 587, 619–625 (2020).
9. Ramsköld, D. et al. Full-length mRNA-Seq from single-cell levels of RNA and individual circulating tumor cells. Nat. Biotechnol. 30, 777–782 (2012).
10. Wu, A. R. et al. Quantitative assessment of single-cell RNA-sequencing methods. Nat. Methods 11, 41–46 (2014).
11. Darmanis, S. et al. A survey of human brain transcriptome diversity at the single cell level. Proc. Natl. Acad. Sci. U. S. A. (2015) doi:10.1073/pnas.1507125112.
12. Fernandez-Godino, R., Garland, D. L. & Pierce, E. A. Isolation, culture and characterization of primary mouse RPE cells. Nat. Protoc. 11, 1206–1218 (2016).
13. Kang, J. et al. In vivo self-renewal and expansion of quiescent stem cells from a non-human primate. Nat. Commun. 16, 5370 (2025).
