# Supplementary material for: A molecular cell atlas of mouse lemur, an emerging model primate
Source: Nature. 2025 Jul 30;644(8075):173–84. doi: 10.1038/s41586-025-09113-9 (PMC12328211; doi:10.1038/s41586-025-09113-9)
Supplement: Supplementary file 2 — Reporting Summary [file 41586_2025_9113_MOESM2_ESM.pdf]

nature portfolio

Corresponding author(s): Mark Krasnow, Stephen Quake

Last updated by author(s): 1/28/2025

Reporting Summary

Nature Portfolio wishes to improve the reproducibility of the work that we publish. This form provides structure for consistency and transparency in reporting. For further information on Nature Portfolio policies, see our Editorial Policies and the Editorial Policy Checklist.

Statistics

For all statistical analyses, confirm that the following items are present in the figure legend, table legend, main text, or Methods section.

n/a	Confirmed	
☐	☒	The exact sample size (*n*) for each experimental group/condition, given as a discrete number and unit of measurement
☐	☒	A statement on whether measurements were taken from distinct samples or whether the same sample was measured repeatedly
☐	☒	The statistical test(s) used AND whether they are one- or two-sided<br>*Only common tests should be described solely by name; describe more complex techniques in the Methods section.*
☒	☐	A description of all covariates tested
☐	☒	A description of any assumptions or corrections, such as tests of normality and adjustment for multiple comparisons
☐	☒	A full description of the statistical parameters including central tendency (e.g. means) or other basic estimates (e.g. regression coefficient) AND variation (e.g. standard deviation) or associated estimates of uncertainty (e.g. confidence intervals)
☐	☒	For null hypothesis testing, the test statistic (e.g. *F*, *t*, *r*) with confidence intervals, effect sizes, degrees of freedom and *P* value noted<br>*Give P values as exact values whenever suitable.*
☐	☒	For Bayesian analysis, information on the choice of priors and Markov chain Monte Carlo settings
☐	☒	For hierarchical and complex designs, identification of the appropriate level for tests and full reporting of outcomes
☐	☒	Estimates of effect sizes (e.g. Cohen's *d*, Pearson's *r*), indicating how they were calculated

Our web collection on statistics for biologists contains articles on many of the points above.

Software and code

Policy information about availability of computer code

Data collection	Illumina NovaSeq 6000 Sequencing System was used to collect sequencing data.
Data analysis	Custom computer codes are available on Globus (https://app.globus.org/file-manager?origin_id=c9fc0a15-54a0-4182-8d64-fd8afc12f1fc&origin_path=%2F). Additional softwares and packages used are described below. Raw sequencing data were processed by Cell Ranger (v2.2, 10x Genomics) for 10x data and by STAR aligner (v2.6.1a), skewer (v0.2.2), RSEM (v1.3.1), and HTSEQ (v2.0) for smartseq2 data. Downstream analyses were performed using R (v4.3.0), Python (v3.6 and 3.9), and Matlab (v2020b). Seurat (R package, v2.3.0), Scanpy (v1.8), and cellxgene (v1.0.1) were used for cell clustering and annotation. Cell gradients were generated using Slingshot (v2.14.0) and a custom program developed in Matlab (Trajectory analysis: https://github.com/Shixuan1/scRNAseq_trajectory_analysis) using Matlab built-in functions (e.g., 'pca'), the Image Processing ToolboxTM (Matlab v2020b), and a Matlab umap package (https://www.mathworks.com/matlabcentral/fileexchange/71902). scRNA-seq data integration used custom programs developed by co-authors, including FIRM (https://github.com/mingjingsi/FIRM) and Portal (https://github.com/YangLabHKUST/Portal). Gene set enrichment analysis used gprofiler2 in R (v0.2.1). For data visualization, dot plots, sina plots, violin plots, line plots, bar plots, box plots, heatmaps, pie charts, error bars and contour figures were generated using Python packages 'pandas' (v1.1.5), 'numpy' (v1.19.3), 'anndata' (v0.7.4), 'scanpy' (v1.6.0), 'matplotlib' (v3.3.2), 'igraph' (v0.7.1), 'seaborn' (v0.9.0), and 'louvain' (v0.6.1); R packages 'ggplot2' (v3.4.4), 'gplots' (v3.1.3), 'readr' (v2.1.4), 'dplyr' (v1.1.2), 'reshape2' (v1.4.4), 'patchwork' (v1.1.3), 'RColorBrewer' (v1.1.3), 'ggrepel' (v0.9.4), 'aplot'(v0.1.10), 'ggdendro' (v0.1.23), 'Matrix' (v1.6.4), 'here' (v1.0.1), 'pheatmap' (v1.0.12), tidyr (v1.3.0), and 'cowplot (v1.1.1); and Matlab built-in functions 'plot', 'scatter', 'violinplot', 'imagesc', 'contour', 'bar', 'box', 'errorbar' and 'pie'.

For manuscripts utilizing custom algorithms or software that are central to the research but not yet described in published literature, software must be made available to editors and reviewers. We strongly encourage code deposition in a community repository (e.g. GitHub). See the Nature Portfolio guidelines for submitting code & software for further information.

Data

Policy information about availability of data

All manuscripts must include a data availability statement. This statement should provide the following information, where applicable:

- Accession codes, unique identifiers, or web links for publicly available datasets
- A description of any restrictions on data availability
- For clinical datasets or third party data, please ensure that the statement adheres to our policy

Tabula Microcebus mouse lemur scRNA-seq gene expression counts/UMI tables, and cellular metadata used in this study are available on Figshare (https://figshare.com/projects/Tabula_Microcebus/112227), and can be explored interactively using the UCSC Cell Browser on the Tabula Microcebus portal (https://tabula-microcebus.ds.czbiohub.org/). Histological atlas of all tissues analyzed is also available on the portal. Raw sequencing data (fastq files) are available on Globus (https://app.globus.org/file-manager?origin_id=c9fc0a15-54a0-4182-8d64-fd8afc12f1fc&origin_path=%2F).
For sequence alignment, Microcebus murinus genome assembly (Mmur 3.0, NCBI accession: GCF_000165445.2) and gene annotation file (NCBI Refseq Annotation Release 101) were obtained from NCBI's FTP sites (https://www.ncbi.nlm.nih.gov/datasets/genome/GCF_000165445.2/; https://ftp.ncbi.nlm.nih.gov/genomes/all/annotation_releases/30608/101/).
For cross-species analysis, human 10x data were from the Tabula Sapiens for the liver, spleen, and bone marrow (https://figshare.com/projects/Tabula_Sapiens/100973) and the Human Lung Cell Atlas for the lung (https://www.synapse.org/#!Synapse:syn21041850/wiki/600865). Human and rhesus macaque testis drop-seq data were from Shami et al. (https://www.ncbi.nlm.nih.gov/geo/query/acc.cgi?acc=GSE142585). Crab-eating macaque lung 10x data were from Qu et al. (https://zenodo.org/record/5881495#.ZERMCnbMKUk). Mouse data were all from 10x data of the Tabula Muris Senis (https://figshare.com/articles/dataset/Processed_files_to_use_with_scanpy_/8273102/2), except for the testis which was based on 10x data from Ernst et al. (https://www.ebi.ac.uk/biostudies/arrayexpress/studies/E-MTAB-6946).
For orthologous genes compilation, mouse lemur genes and corresponding human/mouse orthologs were obtained from NCBI (gene_info.gz and gene_orthologs.gz from https://ftp.ncbi.nlm.nih.gov/gene/DATA/) and Ensembl Biomart (Ensembl Genes version 99). List of human genes with associated genetic disorders was obtained from Online Mendelian Inheritance in Man: (genemap2.txt from https://www.omim.org/downloads).
Source data for figures are provided with this paper.

Human research participants

Policy information about studies involving human research participants and Sex and Gender in Research.

Reporting on sex and gender	N/A
Population characteristics	N/A
Recruitment	N/A
Ethics oversight	N/A

Note that full information on the approval of the study protocol must also be provided in the manuscript.

Field-specific reporting

Please select the one below that is the best fit for your research. If you are not sure, read the appropriate sections before making your selection.

☒ Life sciences ☐ Behavioural & social sciences ☐ Ecological, evolutionary & environmental sciences

For a reference copy of the document with all sections, see nature.com/documents/nr-reporting-summary-flat.pdf

Life sciences study design

All studies must disclose on these points even when the disclosure is negative.

Sample size	A total of 4 mouse lemurs were used in this study. The sample size is determined by the availability of the animals in accordance with the approved animal protocol.
Data exclusions	During pre-processing of single-cell RNAseq, some cells were identified as low quality, doublets, and/or sequencing contaminates (see description in Methods). In follow-up analysis, such data were excluded and indicated in the corresponding Methods and/or Figure Legend.
Replication	4 mouse lemur individuals were used as biological replicates in this study. The number of individuals profiled for each tissue is indicated in Fig. 1c (tissues without a biological replicate are indicated with an asterisk). To ensure consistency across replicates, all scRNA-seq data were integrated together into the same UMAP embedded space (Extended Data Fig. 1c). We confirmed that the same cell types from different individuals clustered together. Cell types that were found in only one individual and clustered separately were assigned a unique cell type designation (indicated with asterisk in Supplementary Fig. 1). Downstream analyses were done with the combined dataset across all individuals. Exception included: cell trajectory analyses which were separated by individual (Fig. 2b-c,e) but with results consistent across replicates (Extended Data Fig. 2f-k); spermatogenesis analyses (Fig. 2d, 5b-c) with testis data available for only one individual; and unknown cell type analysis (#15, #16 and #142, Extended Data Fig. 4c-e, Supplementary Fig. 3c-h) found in only one individual.

Randomization	Animals were not randomized in this study as no hypothesis was being tested; this study focuses on data mining and analysis.
Blinding	This is not applicable as the study does not involve allocation of participants/samples.

Reporting for specific materials, systems and methods

We require information from authors about some types of materials, experimental systems and methods used in many studies. Here, indicate whether each material, system or method listed is relevant to your study. If you are not sure if a list item applies to your research, read the appropriate section before selecting a response.

Materials & experimental systems

n/a	Involved in the study	
☒	☐	Antibodies
☒	☐	Eukaryotic cell lines
☒	☐	Palaeontology and archaeology
☐	☒	Animals and other organisms
☒	☐	Clinical data
☒	☐	Dual use research of concern

Methods

n/a	Involved in the study	
☒	☐	ChIP-seq
☐	☒	Flow cytometry
☒	☐	MRI-based neuroimaging

Animals and other research organisms

Policy information about studies involving animals; ARRIVE guidelines recommended for reporting animal research, and Sex and Gender in Research

Laboratory animals	Experimental species is gray mouse lemur (Microcebus murinus). Lemur 1: male, age 9.8 yr; Lemur 2: female, age 10.1 yr; Lemur 3: female, age 11.8 yr; Lemur 4: male, age 11.8 yr.
Wild animals	The study did not involve wild animal.
Reporting on sex	Two female and two male animals were sampled in this study. No sex-based analysis were performed given small sample size.
Field-collected samples	The study did not involve field collected samples.
Ethics oversight	The study was performed with approval by the Stanford University Administrative Panel on Laboratory Animal Care (APLAC #27439) and in accordance with the Guide for the Care and Use of Laboratory Animals.

Note that full information on the approval of the study protocol must also be provided in the manuscript.

Flow Cytometry

Plots

Confirm that:

☒ The axis labels state the marker and fluorochrome used (e.g. CD4-FITC).

☒ The axis scales are clearly visible. Include numbers along axes only for bottom left plot of group (a 'group' is an analysis of identical markers).

☒ All plots are contour plots with outliers or pseudocolor plots.

☒ A numerical value for number of cells or percentage (with statistics) is provided.

Methodology

Sample preparation	Tissues were processed into single cell suspension as described in Methods. Prior to sorting, cells were stained with SYTOX dye as a dead-cell indicator. Note that flow cytometry used in this study is only for the purpose of sorting single cells to 96- or 384-well plates for Smartseq2 single-cell RNAseq, without further selection.
Instrument	Sony SH800S
Software	Sony SH800S build-in software
Cell population abundance	Cells were only sorted for viability and singlets and specific populations were not selected.

Gating strategy	Cells were only gated for viability and singlets.

☒ Tick this box to confirm that a figure exemplifying the gating strategy is provided in the Supplementary Information.
